# Management of maternal depression: Qualitative exploration of perceptions of healthcare professionals from a public tertiary care hospital, Karachi, Pakistan

**DOI:** 10.1371/journal.pone.0254212

**Published:** 2021-07-07

**Authors:** Makkiya Jawed, Nousheen Akber Pradhan, Rozina Mistry, Amirah Nazir, Sualeha Shekhani, Tazeen Saeed Ali

**Affiliations:** 1 Sehat Kahani Community Innovation Hub, Karachi, Sindh, Pakistan; 2 Department of Community Health Sciences, Aga Khan University, Karachi, Sindh, Pakistan; 3 School of Nursing and Midwifery, Aga Khan University, Karachi, Sindh, Pakistan; 4 International Internship Programme, Aga Khan University, Karachi, Sindh, Pakistan; 5 Center of Biomedical Ethics and Culture, Sindh Institute of Urology & Transplantation, Karachi, Sindh, Pakistan; Marie Stopes International, PAKISTAN

## Abstract

The lack of implementation and routine screening of management techniques at tertiary care hospitals leads to an increased burden of maternal depression. The consequences are borne emotionally, physically, and mentally by the mother, the child, the overall family, and society. Hence, it is vital to contextualize this mental disorder to design and implement effective healthcare interventions. The study is aimed to assess the knowledge and practices of healthcare professionals, in a tertiary care setting, who deal with depressive symptoms amongst mothers. It gauges whether a psychological screening criterion is being implemented by the clinical staff during prenatal and postnatal visits to recommend steps that can help develop a service framework. A qualitative, exploratory study design was implemented for this research. With purposive sampling, eight in-depth interviews (three nurses and five doctors) at a single tertiary care hospital were conducted categorically using a semi-structured (open and close-ended questions) interview toolkit. Content Analysis was carried out using information gathered from the unit of analysis. The study provided evidence of the existing gaps in one particular tertiary healthcare system, within Pakistan, concerning diagnosis and management of maternal depression. Results highlighted that providers were well-versed with explanations of maternal depression, the aftermath of it, and the current status of healthcare; however, they were minimally educated about the specifics and levels of treatment. The gathered information assisted in recommending steps to develop a service framework.

## 1. Background

Maternal depression (MD) represents a world-wide public health problem. Globally, ten percent of expectant mothers and 13% of new mothers experience symptoms of a mental ailment, primarily depression. MD is associated with a major reduction in the quality of life and functional capacity for a woman as well as for her infant physically and psychologically [1, 2] Despite the high prevalence of depression among mothers, there are widespread stigma and lack of awareness regarding the management of MD in low- and middle-income countries [3]. Therefore, many instances of MD go un-noticed or are dealt with poorly when treatment is sought. This raises important questions about the ability of these countries’ existing health systems to treat and manage MD.

According to recent literature, some low- & middle-income countries are moving towards the integration of MD into primary care systems and existing maternal health framework [4]. A randomized control trial was carried out in Chile, in 2007, to assess the benefits of stepped care (a healthcare model focused on delivering/monitoring treatments by ensuring the provision of the least resource-intensive and the most effective treatment in chronological order). Thus, using a multi-component intervention approach, psychoeducational peer-groups, adherence support groups, and pharmacotherapy were integrated into the maternal care plan provided to mothers. The Edinburgh postnatal scale was used to assess the significance of the intervention. Results highlighted a significant decrease in depression levels in the intervention group [5].

In Pakistan, prenatal MD has a prevalence of 27% to 62% [6], while postpartum depression sits at a prevalence rate ranging from 28% to 63%, placing it as the highest in Asia [7]. Much like Chile, similar steps have been taken to integrating MD into the Pakistani health framework. In 2008, a cluster randomized control trial was conducted in Rawalpindi, a low-income, rural community of Pakistan. The research supported the integration of a cognitive behavior change, therapy-based intervention into existing health protocol for primary health workers. In the last month of pregnancy, an intervention session was conducted each week. After delivery, three sessions were conducted during the first month and one session each for the next nine months. The aim was to assess the impact of counseling amongst mothers in their third trimester of pregnancy via the Lady Health Worker program’s doorstep visits. The intervention sessions resulted in a reduction of depression by half in mothers [8]. This showcases the dire need for the employing of adequately skilled staff in managing MD in the existing health framework. Moreover, the mothers provided with treatment were more socially and emotionally functional and had lesser chances of suffering from MD/other mental impairments. The overall effect on the child was also statistically significant. It was found that parents spent more quality time with the infant and, therefore, fewer episodes of diarrhea and better immunizations were recorded [8].

Also, in 2017, a nested qualitative study was carried out as the first step within an outpatient unit in a tertiary care setup in Pakistan. The study aimed to assess the experiences of mothers facing perinatal depression, hindrances in seeking healthcare, and factors affecting the acceptability rate for psychosocial interventions. The intervention group was provided with a pictorial calendar, highlighting eight successive stages within a child’s life during the first three years. Moreover, they were provided with CBT to help deal with their negative thoughts by transforming them into positive ones. The environment of their homes transformed positively and they developed strong bonds with their children, showing significant impact [9].

Despite the small push towards an integration of some intervention strategies, evidence is lacking regarding fully-equipped and numerous interventions within tertiary care in Pakistan. This follows the idea that, for a country like Pakistan, where universal health coverage is still not available and more than 75% of healthcare expenditures come out of the patient’s pocket, MD is an added burden on family economics [10]. Hence, indicating a social aspect in seeking MD treatment that does not seem like a priority for policy-makers and planners in healthcare, which will be discussed later. To tackle these predicaments, and others surrounding proper care for MD, it is important to look to those directly involved in the MD treatment process, specifically, healthcare professionals (HCPs). HCPs provide valuable perspectives on the medical parameters surrounding MD and on the degree of care administered while providing a stepping-stone to enact effective MD interventions tailored to patients in the region. Thus, through this study, the aim is to understand the attitudes and practices of HCPs, who are front-line to encountering MD, to help reduce its prevalence and create a basis for improving Pakistan’s mental healthcare protocols, leading to better maternal mental health.

## 2. Research objective

1. To understand the perception of HCPs at a public tertiary care hospital in Karachi towards mothers suffering from depressive symptoms and the subsequent care available
2. To assess whether a psychological screening criterion is used and implemented by the clinical staff during prenatal and postnatal visits

## 3. Methodology

“Ethics Review Committee, Aga Khan University. 4809-CHS-ERC-17, The study was given approval for a period of one year with effect from June 20, 2017. Written consent.”

### 3.1. Study design

The research was conducted using a qualitative exploratory design. This design evaluates the relationship between HCPs and their ability to assess and manage MD. Hence, this evaluation allows for a starting point to improving healthcare systems that deal with mothers suffering from mental ailments. Identifying key issues and variables not already highlighted by literature is important to understand any phenomenon. As stated by the above literature, little contextual information can be found on the management of MD by HCPs in Pakistan [11].

### 3.2. Study setting

The study was conducted in a Gynecology Ward of Jinnah Postgraduate Medical Center (JMPC), located in Karachi. The tertiary care hospital mostly caters to low socio-economic classes. It was chosen as the study setting because of its fairly large Gynecology OPD ward that caters to a high volume of patients (400–500 mothers daily). On average, eight to ten healthcare providers are always available.

### 3.3. Study participants and sampling strategy

The target population was interviewed using the purposive sampling technique. Doctors and nurses working in the Gynecology OPD of JPMC during the daytime were recruited for the study. This sample reflects the number available of HCPs that could potentially attend to a patient and reflects the work volume on a normal day. Exhibited by Tables 1 and 2, key informant interviews were conducted with eight stakeholders who were available during the data collection period (July to September 2017) and met the eligibility criteria:

**Table 1:** Grid for key informant interviews*.

Positions	Number of participants interviewed	Codes (for identification)	Years employed at JPMC (years)
**Head of Department**	1	D1	22
**Nurse Supervisor**	1	N1	4
**R4 Residents**	2	D2, D3	2 and 4
**R3 Residents**	1	D4	3
**R2 Residents**	1	D5	4
**Nurses**	2	N2, N3	7 and 8–10

*All participants were female with ages from 26 to 48 years.

**Table 2:** Eligibility criteria of study participants.

Inclusion Criteria*	Exclusion Criteria*
• Doctors and nurses with more than a year’s experience in the Gynecology ward at JPMC	• Part-Time employees with less than a cumulative one-year experience
• Residents (year 2,3 & 4) and Gynecologists	

*Criteria is based on medical experience and on-the-job experience in dealing with the patient population. Higher professional education and more than a year’s worth of gynecology experience was preferred.

### 3.4. Data collection instrument

Based on the WHO’s Health System Service Framework, parameters around leadership and governance, service delivery, and the health workforce will be explored [12]. Hence, this conceptual framework is used as a foundation for the study.

As per literature, interpersonal communication between researchers and participants can be easily achieved via exploratory research using in-depth interviews [13]. Semi-structured in-depth interviews (a detailed interview toolkit) were conducted with the participants to obtain data pertaining to research aims [Refer to Appendix 5.1.3]. The interview mainly focused on open-ended and closed-ended questions to understanding perceptions and practices of MD (i.e. what is your understanding of health problems facing mothers with MD?, were there cases that impacted you?, etc.); the barriers which lead to poor diagnosis (i.e. are the proper tools used?); hospital management and societal awareness (i.e. does stigma play a role in treatment-seeking?, what kind of treatment is given to mothers?, etc.); and self-efficacy in diagnosing/treating MD (i.e. are doctors capable of diagnosing MD?, what kind of education is given to HCPs?, etc.).

### 3.5. Data collection method

Literature was thoroughly searched and a toolkit was designed to help understand the perceptions of HCPs towards diagnosis and management of MD. After a dry run, the toolkit was modified. Based on convenience, ease, and permissibility, this public hospital was chosen. The Ethical Review Committee’s approvals of both organizations were obtained.

The in-house supervisor was briefed in detail and in-depth interviews were conducted with participants in a closed room. Verbal and non-verbal cues of participants were noted to help data analysis. Observation, written notes, and recorded transcripts were used for transcriptions. Interviews lasted between 20 to 45 minutes.

### 3.6. Ethical considerations

Guidelines ensured participant’s safety on different levels of the research. Thus, informed consent was obtained and all necessary steps were taken to ensure confidentiality, anonymity, and privacy.

### 3.7. Data analysis procedure

Interviews were conducted in English and Urdu. In cases where the local language was used, the transcriptions were coded in English. Data was analyzed manually by re-reading the transcripts multiple times to develop a comprehensive/well-represented picture of the data.

Qualitative Content analysis was carried out to ascertain latent and manifest meanings of transcripts. Different codes represent different meaningful units [14]. The codes were clustered into sub-categories, categories, and themes based on similarity. To reduce the researcher’s bias, the coding, categorization, and thematic formation of the data were conducted by the primary researcher and re-evaluated by the research supervisor and research committee.

To improve the credibility of the study and to avoid irregularities, data source and investigator triangulation were carried out based on the perspectives obtained by doctors and nurses.

## 4. Results

All participants were females aged between 26–50 years, working in the field of Gynecology for a minimum of two and a maximum of 18 years, having no training/experience within the mental health domain. Five of the participants were doctors, whilst three of them were nurses. Testimonials by HCPs gave insight into the different aspects of treating and recognizing MD. They have been characterized as three themes mentioned in Table 3. These are then detailed into categories and sub-categories that help understand the nature of MD through doctors and nurses.

**Table 3:** Codes developed for data analysis.

THEMES	CATEGORIES*	SUB-CATEGORIES*
**4.1-Multifaceted Nature of Maternal Depression**	4.1.1-Defining Maternal Depression	
	4.1.2-Factors leading to Maternal Depression	
	4.1.3-Consequences of Depression	a) Ramifications for Mothers
		b) Repercussions for Infants
		c) Effects on Caregivers & Society
**4.2-Barriers to Treatment and Management of MD**	4.2.1-Hindrances from Demand Side	a) Stigma & Societal Myths
		b) Levels of Awareness
		4.2.2-Hindrances from Supply Side	a) Lack of Facility preparedness to manage MD
	b) Limited Skills and Abilities of HCPs	
	**4.3-Proposed Interventions for Managing MD**	4.3.1-Treatment Modules	
4.3.2-Need for Capacity Building		

*Each category and sub-category has been split into subsections reporting the doctors’ and nurses’ perspectives separately. This is due to differences in education level between the professions.

The following three key themes were identified after completion of the data analysis:

### 4.1. Theme one: Multifaceted nature of maternal depression

#### 4.1.1. a–Doctors’ perspective—defining maternal depression

All doctors primarily viewed MD as “*a condition*, *which has medical reasons behind it*” (D1), which ranged from baby blues to full-blown depression to depression associated with psychosis, in severe cases (D1,D3,D4). Regarding this, a biological model is usually employed in this tertiary care hospital, emphasizing the role of genes in MD, making certain women prone to experiencing it during the perinatal period. Another biological factor stressed was the physical and hormonal changes affecting women during pregnancy and after pregnancy. This ranged from diabetes and hypertension to chlamydia-like infections (D4, D5). Common symptoms seen by all doctors include “*crying behavior*”, “*keeping quiet*” and disturbances in sleep. The mother may “*not taking interest in herself as well as in her baby and also not paying attention towards the family*” (D2). Participants also reflected on the rates of this condition, with D4 stating her own experience, “*it usually occurs in all females or at least in 90 percent or 80 percent*”.

Physicians agreed that prenatal depression denotes the pre-birth timeline, and postnatal refers to the period after birth. Doctors believed postnatal depression to be more common compared to prenatal depression, based on their experience. D1 emphasized, *“we don’t come across prenatal depression*, *but a lot of anxieties in prenatal cases—as far as I know in my specialty*, *but we do come across a lot of postnatal depression”*. This indicates the presence of more doctors involved in the mother’s post-natal period compared to the pre-natal.

The timeline of MD was also explored, and according to participants, it was varied. For prenatal depression, participants commented that this can last anywhere between a “*week to seven weeks”*. For postpartum psychosis, few stated that it can “*last up to a year”*. However, others stated that “*in cases who are very resistant all who end up in psychoses might end up to a longer period*” (D1), meaning up to a year.

#### 4.1.1. b–Nurses’ perspective—defining maternal depression

In comparison, to the perspective of the doctors, the nurses viewed MD as defined as social and economic stresses that come with raising a child, rather than as a medical dysfunction. This was highlighted based on the nurses’ experiences with mothers seeking ways to give away their children or expressing their desire to stay in the hospital because their homes were not safe (N2, N3). However, nurses also cited similar physical symptoms including raised blood pressure (N1, N2, N3), poor diet (N2, N3), psychosis, sleeping issues, and irritability (N3) which would concern HCPs to suspect MD.

Nurses also differed from doctors in their experience with pre-natal and post-natal depression, leading them to view prenatal depression as more common than postnatal. N1 stated, “*when she relaxes and the baby is delivered*, *half her depression is cured*. *As soon as she sees the baby*, *her mood improves*”. In terms of timeline, N3 noted that pre-natal depression started in the last trimester, however, the other nurses were unclear about timing. This suggests that nurses are more present in the pre-natal periods, and much like doctors, have little knowledge of the full definition of MD.

#### 4.1.2. a–Doctors’ perspective—factors leading to MD

All participants mentioned variability that came with the factors that contribute to MD. Existing health conditions were considered risk factors for MD by all doctors, which included previous instances of depression (D2, D3). However, participants stressed extensively on the pregnancy itself, the added responsibility of a new child, and socio-economic factors that are not in the mother’s favor. In terms of pregnancy, many mothers struggle to conceive, as stated by D3, “*it would be time to conceive for some time*, *it could be due to infertility*”, which would trigger MD. Additionally, the process of pregnancy and birth was viewed to influence the occurrence of depression, as stated by D4, *“sometimes it starts before you go into labor because you are worried of what will happen”*, since mothers must take on new responsibility. HCPs also believed that in the situation of an accidental and often “*unwanted pregnancy*” (D2), mothers experienced these problems quite commonly. Experiencing motherhood for the first time, especially concerning responsibilities attached to this role (D5), was also cited as a reason for experiencing depression.

Socio-economic status was also considered. Financial issues contribute to a woman’s worry, particularly concerning child-rearing and providing for the child’s future needs. Economic conditions combined with lack of social support were also perceived to be a major influencer “*especially if they are from a very lower social economic status*” (D3). A cultural aspect relates to this as HCPs believed that, within Pakistan, the pressure to give birth to a male offspring inculcated worry (highlighting vulnerability) in mothers. D2 explained that, *“many households [in Pakistan] have a concept that the child should be a male*. *Females go into depression fearing the reaction of the in-laws if she gives birth to a female child*”. The husband’s behavior towards his wife, with respect to financial and emotional support, was also considered an important reason, as he is forced to take on her role which can disrupt the dynamics of a household (D1, D3).

#### 4.1.2. b–Nurses’ perspective—factors leading to MD

The nurses reiterated similar views to the doctors by describing medical commonalities seen concerning mothers with pre-natal MD. N2 reiterated the idea of past depression due to previous miscarriages, in addition to hormonal changes in pregnancy, cardiac issues, and chlamydia, while N3 added, “*nutritional patterns change and mothers might become anemic or hypertensive…her labs might show abnormalities”*. Overall, this indicated that MD can be a result of variations of medical anomalies that occur during pregnancy along with lifestyle changes, such as not getting enough nutrition. Again, post-natal was not highlighted as much due to the lack of experience nurses had in dealing with it.

Much like the doctors, nurses stated the mother’s worry about socio-economic support (N1, N3) and needs of the family not being met (N1, N2) as stressors that contribute to MD.

#### 4.1.3. a–Doctors’ perspective—consequences of maternal depression

*a) Ramifications for mothers*. According to all interviewees, MD affects pregnant/new mothers physically and emotionally. Physically, D4 explained that “*they are unable to have proper meals*, *they are anemic*”, causing her overall well-being to decline, while also “*feeling of loneliness*, *tiredness*, *fatigue sometimes like crying for no reason”*. Therefore, she is unable to “*participate in the day-to-day activities*” (D3).

Emotionally, D1 emphasized, “*she’s suffering from symptoms like she’s not taking interest in herself as well as in her baby…not paying attention towards the family and she’s not taking proper medication or she’s lost her appetite and all and she shows signs of weakness and fatigue and tiredness and insufficient sleep”*. This contributes to her feeling that *“she can’t be cured and that she will suffer for eternity”* leading to *“feelings of helpless*, *loneliness and tiredness”*. Women suffering from MD are then caught in a cycle of *“low moods”* /*“mood swings”*. Apart from this, D1 and D3 stated that the mother feels alienated from her child and family, causing her to lose confidence and feel guilty about her ability to be a mother.

In the more extreme cases, women suffering from MD may experience suicidal ideation, or indulge in self-harm. D2 mentioned her personal experience with *“[her] friend…she was a victim of postpartum depression; her sister is now facing postpartum depression… she attempted suicide”*. A woman may also experience homicidal feelings towards her child where *“there is a tendency that a woman may want to kill her child*, *as well*” (D2).

*b) Repercussions for infant*. Interviews revealed consistent explanations from participants regarding the influence of the mother’s state on the child. Participants reported several instances *“where… the mother has been very abusive to the child*, *or she has been beating the child*, *or hurting the baby”* (D1). MD also contributes to short- and long-term consequences for the child, including *“poor growth or poor nutrition later and even death of such children”* (D1). Other reported effects include *“morbidities like diarrhea and pneumonia” that “may require multiple hospital visits”* (D3). It was also noted that with multiple children, mothers “*have the lack of bonding they have with their children so that’s [why] they feel alienated with there off springs*” (D3), also leading to the feeling of neglect on that part of the children.

*c) Effects on caregivers and society*. Doctors mentioned MD’s impact as emotionally and financially burdening on the family and caregivers. The family may feel aggressive towards the mother or they may try to confront their guilty conscience negatively “*because they feel so helpless*” (D2). From the perspective of D1, “*obviously*, *they would also be thinking that what wrong did they do to this lady… there may be depression in themselves* … *and this guilt that what did we do*”. However, other family members may also demonstrate support, especially *“if they are truly concerned and are loving*, *if they are sincere*, *… then they will obviously feel it [mother’s pain and suffering]*. *They will go through the same pain that she is going through”* (D5). In cases of mothers having other children, *“they will be affected first and foremost”* since *“they will not get proper time from the mother”* leading to *“lack of focus on their studies” (D5)*. In some severe cases, it may cause depression in other children, *“because they will not understand what has happened to their mother”* (D3).

They also noted that caregivers can feel emotionally strained, especially when a mother is not responding to care. As stated by D1, “*we do think that what did we do wrong to this lady that this happened to her was it something wrong in my behavior or my care*”. However, they focused on family life being affected more significantly.

Financial conditions of the family may be affected due to extra help needed with child-rearing and house responsibilities since the mother is “*not participating in the day-to-day activities*” (D3). D4 mentioned “*If the mother is not well she may not be able to take care of the baby*. *Ultimately*, *the family will need a maid*, *or they*, *you know*, *they will need to help her in doing things… if she is very sick*, *the husband may need to stay home and miss work*”.

Doctors highlighted some societal impacts when one participant responded, *“There have been cases where the kid has been abandoned by their parents at some orphanages due to that…*.*and it increases the society’s burden”*.

#### 4.1.3. b–Nurses’ perspective—consequences of maternal depression

*a) Ramifications for mothers*. Participants expressed ramifications as being physical and emotional. In the physical sense, nurses highlighted high blood pressures as a major development in MD, as N1 explained, “*health wise*, *[the mother’s] stress levels increase*, *her blood pressure increases…Internally it keeps on eating at her*. *She falls sick”*. N3 validated this by stating, “*when we assess the mother’s vitals*, *her blood pressure levels are high*”. They also all agreed that the mother’s health declines with MD, especially if she has a poor diet.

Emotionally, the mother becomes agitated with herself (N1) and can no longer take care of her child; essentially, disrupting her daily routine (N2, N3). As a result, she “*lacks health-seeking behavior”* (N1), which isolates her and can worsen MD symptoms.

*b) Repercussions for infant*. Nurses mentioned that the child would experience poor diet (N3), however, they focused on the personality aspect of the child. N1 stated, *“we say that the child learns when in the mother’s womb…This is natural…and the child will become irritable [due to mother’s irritability] when he grows up*.*”* This comes from the idea that the child fails in seeking attention from the mother and has no other source of comfort to turn to. Similar to the doctors, nurses also mentioned that older children are affected because they also lack attention from the mother, while attempting to take on responsibilities that she may be neglecting (N3).

*c) Effects on caregivers and society*. Participants similarly commented on the effect MD has on family life, especially when the family refuses to allow the woman to seek proper treatment. N3 cited the idea that families deal with mental health as purely a religious issue and essentially do not make accommodations for the mother.

In comparison, the doctors saw no connection to religious beliefs and deemed the family burden as a result of not fulfilling cultural responsibilities. This then falls on other members of the family. The nurses also indicated that they saw no burden to HCPs as a result of MD.

### 4.2. Barriers to treatment and management of MD

#### 4.2.1. a–Doctors’ perspective—hindrances from demand-side

*a) Stigma & societal myths*. Due to the social stigma within Pakistani society, the existence of MD is ignored and a woman’s ability to seek care for depression is limited. Furthermore, patients and family members do not acknowledge the ailment, and prefer to remain quiet about it because of fear of being frowned upon by society (degraded in social acceptability). D3 stated, *“because of no focus on mental health… there is also no focus on maternal depression”*, few people actively seek care for MD, so *“mostly people who seek help are those who are at a severe stage or because healthcare professionals have referred them to another specialist”*. D4 reiterated this by highlighting “*most of the families don’t believe that its depression and don’t care and so it is neglected*” (D4). D1 stated that it has gone so far as to family members taking children away from their mothers and labelling their condition as “*vision problems”* to avoid addressing the problem.

Superstition and cultural pressure also contribute to this reluctance to seek care for MD. *“Women do not go for treatment for depression because of myths around supernatural powers…they prefer a religious scholar for seeking treatment*,*”* thus pushing the woman to *“further go into depression”*, as mentioned by D2. Even if a mother understands MD as a medical problem they do not seek help because “*people might think that they will be labeled as mental people or psychologically ill people or weak people all this might be a hindrance*” (D1).

*b) Level of awareness*. Interviews revealed that awareness levels about MD (ie. prevalence, effects, etc.), among women and their families, contributed significantly to whether or not they would seek treatment. Treatment tends to be sought, *“when they’re suffering from a cardiac emergency*, *they will definitely take her to the hospital because that is life-threatening”* (D2). Then again, mental illnesses *“are very slow…in terms of progress*.*”* Mental illnesses are often viewed as a pretense by the women to *“avoid the daily household activities and she’s just making excuses and she’s just portraying it”* (D1). Family members often do not see that *“she [the mother] is actually suffering from a very devilish disease*, *which can affect her life and the baby’s as well as the family’s life”* (D2). However, if a mother does manage to seek treatment, she often complains about “*domestic abuse and some*, *they are like they have some fights and they’re suffering from bad outcomes*” (D2), and does not recognize that MD may be at the root of her problems.

The role of the mothers-in-law is also crucial. They may say, for example, *“that we have delivered 7 or 8 children…*.*we never experienced this”* and *“when we did this*, *we were taking care of the baby and doing household work”*, as mentioned by participants. Even amongst the more educated families, it is reported, mothers-in-law still exhibit negative remarks such as, *“this is self-created…”* and that *“is a new generation’s thing*, *because they have Google and they search things”* (D2).

#### 4.2.1. b–Nurses’ perspective—hindrances from demand-side

*a) Stigma and societal myths*. Similarly, to the perspective of the doctors, nurses reiterated that lack of family support especially from the husband or male guardian (N1, N3) plays a role. Many times, the husband is in charge of family dynamics, which includes how his wife may conduct her outings. N3 also mentioned that MD is considered a religious problem that can discourage a mother from seeking proper treatment. However, one a mother is not responding to religious advice she is blamed for not taking care of herself (N1).

*b) Level of awareness*. Due to high illiteracy levels and the marginalization of mental health, women themselves are not aware enough “to go to a doctor and seek treatment. They are not educated, they do not know where to go [even if they feel there is a problem]. They need someone to guide them” (N2). Therefore, they become dependent on others to help them with treatment appointments and other care. This perspective of the nurses differs from the doctors because the doctors did not cite education levels as a hindrance to treatment. Sometimes, when diagnosed, women are faced with remarks from their family members including “do not pay attention to it…act like this is not there”, further deviating them from seeking care.

N3 also highlighted the programs available to mothers in rural areas including, registering with local midwives and door-to-door care. However, since mothers have little knowledge about MD, they may not look for these programs.

#### 4.2.2. a–Doctors’ perspective—hindrances from supply-side

*a) Lack of facility preparedness to manage MD*. Interviewees highlighted the high influx of patients in public health settings, making it difficult to provide treatments for mental health. They argued that it was difficult enough to provide treatment for general physical health in such facilities, therefore, MD often goes undiagnosed. As D4 explained, *“increased flow of the patients leads to saturation…*. *Because we are checking 450 to 500 patients per day*, *we are not used to listening to patients”*. This leads to problems in care delivery where *“we are unable to properly look into this matter”* and instead say to *“patients who come in with pains and headaches*,*… that okay this normally happens in pregnancy”*. Although history taking is routine practice, *“we do not focus on depression”* because that *“requires time”* and *“we are short on people”*.

This overcrowding, not only in OB/GYN wards, crowd specialists who are called for consultation, *“the psychiatrist just comes and checks the patients and prescribe some antidepressants”* (D4). The responsibility of said patient experiencing mood difficulties lies not with psychiatrists, but with HCPs in gynecology because *“they are admitted under our care”*. However, three of the doctors mentioned not being able to follow up with patients, especially those who do not end up following prescribed treatments (D1, D2, D3). Participants also cited, not having the proper “*proactive monitoring tools*” (D1, D3, D4) to treat MD as a problem. This was reiterated by D1 who stated using a general Birmingham scale (used for non-MD) as compared to the more specific Edinburgh scale (as seen in MD framework in other countries). In general, apathy towards mental health was also problematic; *“they [healthcare system] does not regard it as a serious entity in itself”* (D3).

*b) Limited abilities and skills of HCPs*. Participants revealed that most practitioners were not well-versed with different treatments available for MD. This is prevalent because current medical and nursing education is not well-equipped to emphasize the importance of emotional/mental health. This occurs at the level of postgraduate training, emphasized by D3, *“there is lack of awareness of this since our college education…then also during our residencies*. *There is no framework for depression to be taught anywhere in medical department or the surgical department”*. All other doctors expressed a similar sentiment. The refinement of skills through refresher training, workshops, and CMEs are also not available, which compounds the issue, as some in the study emphasized. Sometimes the neglect from HCPs is such that *“we are often forced to initiate mental health treatment”* because some patients are *“aware enough to it and they come to us” (D2*, *D3)*. The initiative to diagnose/treat is not often undertaken by mental healthcare professionals because they are not well-versed with treatment modules (D3).

There is also the idea that low-skilled HCPs, such as midwives/birth attendants are usually sought throughout the pregnancy and birth process because of their easy access, noted by participants. But they “*are not equipped to deal with these problems [MD]” (D3)* and since a majority of mothers get what they need out of these midwives/birth attendants they do not go to the hospital unless “*bleeding occurs*” (D3). Since mental healthcare is not vigorously sought in healthcare facilities, women who are *“fortunate enough to deliver in a hospital may get treatment” (D3)* for MD.

#### 4.2.2. b–Nurses’ perspective—hindrances from supply-side

*a) Lack of faculty preparedness to manage MD*. Nurses mentioned similar ideas to doctors citing a large patient-to-staff ratio which forces staff to spend less time with each case (N2, N3). Therefore, the burden of follow-up and care is put on the shoulders of nurses, who may not be familiar with the patient. This is why all participants noted that using compassion and empathy is a major part of their job, to make patients feel more comfortable.

The lack of preparedness in faculty was also expressed as not having the right health assessments to diagnose MD. Even though medical histories are taken, they are not always thorough (N1, N2). However, when MD is picked up, N1 stated that the mother is often sent to a psychiatry/psychology ward because of their lack of proper tools.

*b) Limited Abilities and Skills of HCPs*. Comparably, the perspectives of nurses cited poor education in the medical curriculum on MD. While nurses “are taught to talk to the patient politely, to explain things to the patient, to talk to the patient with love” (N1) (instances of empathy and compassion), more technical skills required to deal with MD are not part of curriculums. They also stated that a few workshops have been held, however, they are not well-attended nor do they have a specific focus (N1, N3). Rather, the information given on mental health is generally from general psychiatry workshops and does not focus on MD.

### 4.3. Proposed interventions for managing MD

#### 4.3.1. a–Doctors’ perspective—treatment models

All doctors expressed the need for communication and education as major treatment options. Employing peer support groups (D1), currently “*not practiced in our pregnant population*” helps in forming connections among people undergoing similar problems, physically and emotionally. This offers guidance to mothers and caregivers. D5 mentioned, “*to treat maternal depression*, *if it is diagnosed at a very low point*,*…we can try counseling because depression can be treated with counseling*.*…with family counseling of immediate caregivers and partners*, *we can treat this thing at a very early point and it can stop it from progressing*. *But if the thing increases a lot*, *patient is following-up very infrequently*, *patient is coming with a very bad case of depression*, *the patient should be referred to a specialist”*. Participants noted the need to involve families in counselling and treatments because, as highlighted by D2, “*she should have a supportive family*, *she should have a husband who can actually take care of her and again”*.

The idea of a ‘one-stop solution’ and collaboration between hospital wards came up. D3 mentioned that *“facilities should be available to her in one place (a single unit or ward)” allowing “easy access whenever at her will” since “going around the society will make it more difficult for her and she probably would not seek help”*. This would include more involvement on the side of psychiatrists and staff in the Gynecology wards, while also implementing educational programs for the mother in antenatal care. In situations where mothers cannot come to sessions frequently, brochures and hotlines should be made available (D3).

#### 4.3.1. b–Nurses’ perspective—treatment models

Nurses highlighted the need for awareness and counselling sessions. Sessions through media should be conducted, “*family planning*, *for example is told about on media*, *but depression should also be targeted*. *This would be helpful*”. This would include acknowledging the mother’s condition and “*speaking to them nicely*” (N1, N2) while supporting them with medication if needed. In the sessions, they noted that education would be needed for mothers during and after pregnancy (N3), that they must involve the family (N2), and that the use of role-playing highlighting MD existence and problems should be applied as it would be “*better recall for patients”* (N3).

#### 4.3.2. a–Doctors’ perspective—need for capacity building

According to a few participants, the system does not allow providers to develop proper relations with patients. Senior doctors are burdened with more administrative tasks, making it difficult for them to provide no more than 10 minutes per patient. This can be problematic for MD as it does not commonly manifest itself physically, unless severe. Doctors must be sensitized and a method of consistent follow-up should be developed since there is no prior screening for MD (D3). D1 explained, “*sensitization can occur all through your life and with any kind of session or any CME or any such activity can sensitize people… A mother has an emotional condition which does have some physical impacts… and one must be able to have a high indication… so that we can pick them early*”. She further explained that such procedures are followed in private practices but not government hospitals arguing; “*Gynecologists usually come across in their public practice obviously we come across such patients not very often but in private practices yes*, *they do come across such patients very often*”. Therefore, faculties need to communicate better and the hospital would need to employ working with non-governmental organizations to push MD framework in health policy.

Doctors also stressed the need for more action on the side of the general practitioner (GP). D3 reiterated this as the “*general practitioner system in our society is still not the way it has to be*. *People do see their general practitioners but even there they don’t come up with a lot of personal problems but still as compared to a tertiary care hospital they might be comfortable in discussing this with their general practitioner*”. Therefore, since more patients are comfortable with seeing their GP, there should be more emphasis on giving them proper tools to diagnose and treat MD.

#### 4.3.2. b–Nurses’ perspective—need for capacity building

Some participants perceived treatment as only possible if an HCP establishes a strong therapeutic relationship with the patient. This requires providing dedicated time to the patient. According to N3, “*when a mother cries*, *regardless of how strong-hearted a person is*, *sympathy comes into play and counseling can be done…Sometimes we are irritated*, *we cannot tell them our problems but we can support them enough that they come towards a healthy lifestyle…”*. This helps in providing patients *“confidence that I [health professional] is with them and I will help them” (N3)*.

Establishing medical relationships were noted as complex due to the little time HCPs have with patients. Nurses suggested that this would be solved by reviewing the medical curriculum. Therefore, “*more colleges and universities should focus on what happen with pregnant women*” to increase knowledge among future patients and incoming HCPs (N1, N2). The use of role-playing would also teach undergraduates and graduates because information “*memorized for the sake of exams*” (N3) will not go a long way in treating patients.

Similar to the doctors’ perspectives, the nurses also wished to see more involvement of gynecologists and general practitioners.

## 5. Discussion

The study highlights the breadth of knowledge HCPs at one tertiary care facility imply when dealing with MD in the context of their Pakistani population. It gives insight into a system in a low-income country with a low budget devoted to healthcare (only 0.14% devoted to mental healthcare) and a multitude of other healthcare problems [15]. This qualitative research explores perhaps representing the first research of this nature, from this part of the world to the best of the investigator’s knowledge.

The literature review conducted illustrated very few interventions with known effectiveness in real-world settings in the context of Pakistan. There has been, however, increasing recognition that this aspect of maternal health requires consideration. Efforts have nevertheless been isolated. Patients who visit a public tertiary care hospital for pregnancy and delivery, and HCPs who work there, represent a small segment of Pakistani society. This is ascribed to deliveries happening at home, performed by midwives (trained and not trained) in most areas of Pakistan, particularly in rural settings [16, 17]. However, in severe and complicated cases, women from rural areas may seek treatment at urban centers [18]. Results should be interpreted with this pertinent context in mind.

### 5.1. Gaps in governance and leadership

As stated earlier, there is room for improvement in terms of strengthening the skills/abilities of HCPs to manage MD. This research reflects gaps within the teaching curriculum for doctors and nurses that restrict mental health care. These practices are instead manifested through their knowledge about certain illnesses, such as hypertension, and their limited on-the-job experience concerning diagnosis and treatment [19]. Mental illness is treated as a symptom and indirectly addressed. All participants, in this study, agreed upon the high prevalence of MD and recognized that it was neglected within their day-to-day interactions with patients. However, the level of knowledge among nurses and physicians varied, as expected. This can be explained by the fact that nurses are available during pre-natal visits and at the time of the delivery and are not present during follow-up visits. Physicians, however, were somewhat present in the pre-natal periods, but majorly present in post-natal visits. HCPs in the study also did not know specific timelines and did not refer to any set diagnostic criteria. This reflects the non-existence of standardized protocols for diagnosing MD in either doctor or nursing practice. Given the context of Pakistan, according to WHO, a small portion of the undergraduate medical curriculum in most medical universities (27% for doctors & 3% for nurses) and postgraduate training programs is dedicated to mental health [20]. Therefore, gaps in governance and leadership, are emphasized [21]. Even when training is provided, as our participants pointed out, there are no Continuing Medical Education (CME) activities/refresher training, which is considered essential for service delivery. However, participants were able to elucidate upon the symptoms of depression, along with probable risk factors (ie. basic knowledge).

### 5.2. Healthcare service delivery

The setting of this study is an extremely busy tertiary hospital that faces staff shortages intermittently [22]. Postgraduate trainees and consulting physicians are overburdened with multiple tasks, high patient volumes, and time constraints. All levels of HCPs are also assigned a variety of administrative tasks, limiting their ability to build proper patient relationships. In this environment, often, priority is directed to the general physical health of the mother and the child, as opposed to mental health [23, 24]. As suggested in the study, participants highlighted the importance of capacity building with patients for better care delivery and outcomes. However, service delivery is known to vary from one setting to the other. Public healthcare facility practices may differ from private hospitals, which may not have staff shortages that significantly limit the role that HCPs play within public hospitals. Patients also tend to pay physician consultation fees in private facilities, perhaps motivating physicians to provide better services, and devoting more time to their patients.

Another drawback within service delivery, identified by participants of the study, included the lack of protocols for screening, treatment, and management of mental disorders. What was encouraging, however, was that many participants identified the problems existing within the system and proposed plausible solutions. Participants presented the importance of stepped care (not using this terminology); an approach gaining popularity within the chronic disease model [25]. As mentioned in the introduction, patients with less severe symptoms are managed by non-specialist healthcare staff, whereas those with more severe symptoms can be referred to a specialist. This saves time and resources through medical collaborations; an important consideration within the resource-constrained settings of public hospitals. This model seemingly works well in LMICs, such as South Africa which recently implemented a stepped care intervention to address maternal mental health in their Mowbray Maternity Hospital. They have recently found more women wanting to attend their sessions with 91.7% reporting positive moods with attendance. More women have also reported lifestyle issues, such as abuse, leading to better care [26]. An implementation like this is encouraging because it shows that similar models can be administered in Pakistan with similar resource settings.

### 5.3. Health workforce

Pakistan lacks sufficient human resources to tackle issues within the mental health domain. Few tertiary care hospitals, all based within Karachi, offer community services. Last year, only 3 psychiatrists were able to graduate from renowned educational institutions. The city center houses nearly 2.29 times more psychiatrists as compared to the rest of the country. Only a handful of psychologists and social workers can be found working within the government sector [27]. This lack of qualified and dedicated staff in JPMC was identified as an issue by the participants. To counter this problem, participants proposed utilizing GPs in diagnosing and management. Since specialists (those within gynecology as well) face heavy patient loads, it has been recommended that GPs be trained to screen for these conditions, saving resources and time [28]. If patients visit their GPs for management of conditions such as depression, classified under Common Mental Disorders (CMDs), there is less strain upon the already burdened tertiary healthcare system. Literature has previously identified that CMDs, including MD, can be treated at the primary healthcare level [29]. Moreover, participants emphasized, in Pakistan, women often visit their GP during pregnancy and only refer to a hospital for delivery [30].

### 5.4. Gaps in contextual research

Participants emphasized preventive efforts, as opposed to reactionary efforts. MD, as identified by our study participants causes significant stress upon different stakeholders within the society, including mother and child, family members, the healthcare system, and society as a whole. Preventive efforts can include Information Education and Communication packages when mothers visit for the first time. However, according to a systematic review on the barriers and facilitators of seeking help for mental issues, the effectiveness of this approach varies according to the literacy rate of patients [31] and socio-economic status.

### 5.5. Social factors

From the perspective of participants, HCPs and the health system both need to be mindful of the barriers patients face when trying to access mental health treatment. Social factors are an important consideration that this research stressed upon. Help-seeking behavior, especially within mental health, is mediated by various socio-cultural factors [32]. This includes, as found in this study, the influence of family members, particularly mothers-in-law, who tend to dismiss MD. Depression may not be understood by family members as a ‘problem’, since childbearing is considered a ‘natural’ part of a woman’s life. A woman exhibiting feelings of dissatisfaction during pregnancy or after birth may be misconstrued by the family, causing MD to be overlooked [33]. Therefore, it is worthwhile for HCPs to involve family members in counselling sessions and peer support groups.

Another factor mediating help-seeking behavior is the stigma associated with mental illness. Depression, particularly associated with psychosis, may be attributed to a supernatural phenomenon and not treated as a mental condition [34]. Such myths should be considered by HCPs and dispelled through education. Furthermore, there is a reluctance to seek treatment from mental healthcare professionals because they do not want to be labelled as sick when they see themselves as physically healthy. They also fear being frowned upon by the community, as mentioned by participants. Therefore, MD recognition and treatment should happen within non-stigmatizing domains, such as within general healthcare, incorporated into maternal and child health programs, or during antenatal and follow-up visits to the gynecologist.

As our participants highlighted, mothers may report feelings of helplessness during the prenatal period due to the socio-cultural expectation of bearing a male child. This is true for many South Asian societies [35]. Mothers experiencing dissatisfaction with their new roles may battle feelings of guilt, especially if they did not meet cultural expectations, which require to be dealt with by HCPs; something participants in our study were mindful about. Protocols, such as multi-sectoral collaborations and one-stop solutions, as mentioned in our results, which provide timelines for diagnosis and discussing the symptomatology of MD, must include socio-cultural aspects.

As evidenced through literature from other countries, another barrier includes the cost of traveling to healthcare facilities and treatment [36]. However, despite our study suggesting this, it was not fully covered and may need further research.

### 5.6. Strengths and limitations

This research is a stepping stone for further research into the health framework that manages MD diagnosis and treatment. However, it is limited in sample size since results were only conducted at a single public tertiary care hospital with few participants. Therefore, not all data can be representative of all tertiary care facilities. It is also possible that the study participants were primed due to the briefing provided to the supervisor.

In contrast, the study also provides insight into the different levels of care potentially given to patients with MD by both doctors and nurses in this tertiary care hospital. Their differences in education level and experience express different perspectives to how MD care is conducted allowing others to create a holistic picture of treatment. However, it must be noted that the views of HCPs in this study, may not be representative of other HCPs who deal with different populations in the country. Also, the roles of nurses and doctors in MD care and their availability to those seeking care varies which can confound conclusions surrounding what streamlined care may look like; if any at all. To the best of the researcher’s knowledge, no study within this area has been conducted before within Pakistan in a tertiary care setup.

## 6. Conclusion

The prevalence of MD is high, leading to severe problems for the patient, the patient’s family, the healthcare system, and society. While HCPs demonstrate basic knowledge regarding symptoms of MD, the determinants and risk factors attached to MD, the unavailability of standardized treatment protocols for screening, and the diagnosing and treating MD; there is much contribution to their inability to deal with it in clinical encounters. Time constraints and patient overload were also significant contributors to this inability. Participants also demonstrated a willingness to manage MD in clinical settings but, cited ineptitude to deal with this task due to lack of training during undergraduate, postgraduate medical, and nursing curriculums. The health system should focus on this need for the supply-side to be managed. From the demand side, the healthcare system needs to address the barriers in help-seeking behavior which includes debunking myths and stigmas regarding mental illnesses, the guilt associated with MD, and costs attached in accessing treatment.

### 6.1. Policy recommendations

- Pre-service and in-service education curriculum for all HCPs should be modified to incorporate management tools for common mental health issues, specifically, MD.
- Policies need to be implemented to ensure that mothers and family supports are educated about MD. This can be done through means of prenatal and post-natal classes, public campaigns/different media forums, and family counselling/peer support groups. These should be inclusive to all mothers in different socio-economic strata.
- For service delivery, tools must be institutionalized to include multiple medical contributors (psychiatrists, GPs, gynecologists, etc.) and a variety of diagnostic tools. Overall improvement of health framework should include groups such as non-governmental organizations to push better health policy.

## Supporting information

S1 File Interview transcriptions for doctors and nurses. Files contain the full interviews for each participant. All interview have been translated from the local language used in the interview into English for the sake of this research.
(DOCX)

## References

[1] WHO | Maternal mental health [Internet]. Who.int. 2017 [cited 13 February 2017]. Available from: http://www.who.int/mental_health/maternal-child/maternal_mental_health/en/

[2] Maternal mental health and child health and development in resource-constrained settings. Report of a UNFPA/WHO international expert meeting: the interface between reproductive health and mental health. Report Number: WHO/RHR/09.24,2007.

[3] Patel V. Mental health in low-and middle-income countries. British Medical Bulletin. 2007 Jan 1;81(1):81–96. 17470476 10.1093/bmb/ldm010

[4] Hanlon C. Maternal depression in low-and middle-income countries. International health. 2012 Nov 20;5(1):4–5. doi: 10.1093/inthealth/ihs003 24029837

[5] Rojas G, Fritsch R, Solis J, Jadresic E, Castillo C, González M et al. Treatment of postnatal depression in low-income mothers in primary-care clinics in Santiago, Chile: a randomised controlled trial [Internet]. 2017 [cited 13 February 2017]. Available from: http://www.thelancet.com/journals/lancet/article/PIIS0140-6736(07)61685-7/10.1016/S0140-6736(07)61685-7 17993363

[6] Humayun A, Haider I.I, Imran N, Iqbal H, and Humayun N. Antenatal depression and its predictors in Lahore, Pakistan. Eastern Mediterranean Health Journal.2013;19(4):327–332. Available from: http://www.emro.who.int/emhj-vol-19-2013/4/antenatal-depression-pakistan.html 23882957

[7] Gulamani S, Shaikh K, Chagani J. Postpartum Depression in Pakistan. Nursing for Women’s Health. 2013;17(2):147–152. Available from: https://www.ncbi.nlm.nih.gov/pubmed/23594328 doi: 10.1111/1751-486X.12024 23594328

[8] Rahman A, Malik A, Sikander S, Roberts C, Creed F. Cognitive behaviour therapy-based intervention by community health workers for mothers with depression and their infants in rural Pakistan: a cluster-randomised controlled trial. The Lancet. 2008;372(9642):902–909. doi: 10.1016/S0140-6736(08)61400-2 18790313 PMC2603063

[9] Husain N, Chaudhry N, Furber C, Fayyaz H, Kiran T, Lunat F, et al. Group psychological intervention for maternal depression: A nested qualitative study from Karachi, Pakistan. World journal of psychiatry. 2017 Jun 22;7(2):98. doi: 10.5498/wjp.v7.i2.98 28713687 PMC5491481

[10] Gulamani S, Shaikh K, Chagani J. Postpartum Depression in Pakistan.Nursing for Women’s Health. 2013;17(2):147–152. Available from: https://www.ncbi.nlm.nih.gov/pubmed/23594328 doi: 10.1111/1751-486X.12024 23594328

[11] Creswell JW. Research design: Qualitative, quantitative, and mixed methods approaches. Sage publications; 2013 Mar 14.

[12] World Health Organization. Everybody Business: Strengthening Health Systems to Improve Health Outcomes: WHO’s Framework for Action [internet]. Geneva, Switzerland: World Health Organization. 2007 [cited 23 August 2020]. Available from: https://www.who.int/healthsystems/strategy/everybodys_business.pdf?ua=1

[13] Creswell J.W. Research design: qualitative, quantitative, and mixed methods approaches 2nd ed., CA: Sage: Thousand Oaks; 2003

[14] Graneheim U.H, Lundman B. Qualitative content analysis in nursing research: concepts, procedures and measures to achieve trustworthiness. Nursing Education Today. 2003 October 8, 24, 105–112. 10.1016/j.nedt.2003.10.001 14769454

[15] World Health Organization. WHO-AIMS report on mental health system in Pakistan. Geneva: World Health Organization. 2009.

[16] Jokhio AH, Winter HR, Cheng KK. An intervention involving traditional birth attendants and perinatal and maternal mortality in Pakistan. New England Journal of Medicine. 2005 May 19;352(20):2091–9. doi: 10.1056/NEJMsa042830 15901862

[17] Shaikh BT, Hatcher J. Health seeking behaviour and health service utilization in Pakistan: challenging the policy makers. Journal of public health. 2004 Dec 8;27(1):49–54. doi: 10.1093/pubmed/fdh207 15590705

[18] Qureshi RN, Sheikh S, Khowaja AR, Hoodbhoy Z, Zaidi S, Sawchuck D, et al. Health care seeking behaviours in pregnancy in rural Sindh, Pakistan: a qualitative study. Reproductive health. 2016 Jun 8;13(1):34. doi: 10.1186/s12978-016-0140-1 27356863 PMC4943512

[19] Connelly CD, Baker-Ericzen MJ, Hazen AL, Landsverk J, Horwitz SM. A model for maternal depression. Journal of Women’s Health. 2010 Sep 1;19(9):1747–57. doi: 10.1089/jwh.2009.1823 20718624 PMC2965697

[20] World Health Organization. WHO-AIMS report on mental health system in Pakistan. Geneva: World Health Organization. 2009.

[21] Mosadeghrad AM. Factors influencing healthcare service quality. International journal of health policy and management. 2014 Jul;3(2):77. doi: 10.15171/ijhpm.2014.65 25114946 PMC4122083

[22] Satyanarayana VA, Lukose A, Srinivasan K. Maternal mental health in pregnancy and child behavior. Indian journal of psychiatry. 2011 Oct;53(4):351. doi: 10.4103/0019-5545.91911 22303046 PMC3267349

[23] Lakdawala PD. Doctor-patient relationship in psychiatry. Mens sana monographs. 2015 Jan;13(1):82. doi: 10.4103/0973-1229.153308 25838726 PMC4381325

[24] Rojas G, Fritsch R, Solis J, Jadresic E, Castillo C, González M et al. Treatment of postnatal depression in low-income mothers in primary-care clinics in Santiago, Chile: a randomised controlled trial [Internet]. 2017 [cited 13 February 2017]. Available from: http://www.thelancet.com/journals/lancet/article/PIIS0140-6736(07)61685-7/10.1016/S0140-6736(07)61685-7 17993363

[25] Honikman S., Van Heyningen T., Field S., Baron E., Tomlinson M. Stepped Care for Maternal Mental Health: A Case Study of the Perinatal Mental Health Project in South Africa. PLOS Medicine. 2012 May 29. doi: 10.1371/journal.pmed.1001222 22666181 PMC3362637

[26] World Health Organization. WHO-AIMS report on mental health system in Pakistan. Geneva: World Health Organization. 2009.

[27] Wittchen HU, Mühlig S, Beesdo K. Mental disorders in primary care. Dialogues in clinical neuroscience. 2003 Jun;5(2):115. doi: 10.31887/DCNS.2003.5.2/huwittchen 22034245 PMC3181625

[28] Siriwardhana C, Adikari A, Van Bortel T, McCrone P, Sumathipala A. An intervention to improve mental health care for conflict-affected forced migrants in low-resource primary care settings: a WHO MhGAP-based pilot study in Sri Lanka (COM-GAP study). Trials. 2013 Dec 9;14(1):423. 24321171 10.1186/1745-6215-14-423 PMC3906999

[29] Finlayson K, Downe S. Why do women not use antenatal services in low-and middle-income countries? A meta-synthesis of qualitative studies. PLoS medicine. 2013 Jan 22;10(1):e1001373. doi: 10.1371/journal.pmed.1001373 23349622 PMC3551970

[30] Gulliver A, Griffiths KM, Christensen H. Perceived barriers and facilitators to mental health help-seeking in young people: a systematic review. BMC psychiatry. 2010 Dec 30;10(1):113. doi: 10.1186/1471-244X-10-113 21192795 PMC3022639

[31] Saraceno B, van Ommeren M, Batniji R, Cohen A, Gureje O, Mahoney J, et al. Barriers to improvement of mental health services in low-income and middle-income countries. The Lancet. 2007 Oct 5;370(9593):1164–74. doi: 10.1016/S0140-6736(07)61263-X 17804061

[32] Lafrance MN, Stoppard JM. Constructing a non-depressed self: Women’s accounts of recovery from depression. Feminism & Psychology. 2006 Aug;16(3):307–25.

[33] Burns JK, Tomita A. Traditional and religious healers in the pathway to care for people with mental disorders in Africa: a systematic review and meta-analysis. Social psychiatry and psychiatric epidemiology. 2015 Jun 1;50(6):867–77. doi: 10.1007/s00127-014-0989-7 25515608 PMC4442066

[34] Grech V. A global review of male offspring preference and gender bias in the caring of children.

[35] Patel V, Rodrigues M, DeSouza N. Gender, poverty, and postnatal depression: a study of mothers in Goa, India. American journal of Psychiatry. 2002 Jan 1;159(1):43–7. 10.1176/appi.ajp.159.1.43 11772688

[36] Mahmood N, Ali SM. The disease pattern and utilisation of health care services in Pakistan. The Pakistan Development Review. 2002 Dec 1:745–57.

