# Supplementary material for: Management of maternal depression: Qualitative exploration of perceptions of healthcare professionals from a public tertiary care hospital, Karachi, Pakistan
Source: PLoS One. 2021 Jul 7;16(7):e0254212. doi: 10.1371/journal.pone.0254212 (PMC8263250; doi:10.1371/journal.pone.0254212)
Supplement: S1 File — Files contain the full interviews for each participant. All interview have been translated from the local language used in the interview into English for the sake of this research. (DOCX) [file pone.0254212.s001.docx]

| Interview Transcriptions for Doctors and Nurses | |
| --- | --- |
| Dr Anum  *Briefing provided - verbal and written consent obtained* | |
| Interviewer: So doctor sahiba mujhy pehle ye bataiye ke what is your understanding of maternal depression? |  |
| Interviewee: I think maternal depression is when you carry a baby and when you go through physical, different physical changes and that is a biggest part of your life of a womanhood. To Kuch changes ese hote hain jo ap accept nahi kar pate ya kuch distressing cheezein hoti hain apki body ke sath ya apke environment me ya is tarah ka kuch that causes depression, that is not feasible for you and that causes pain and matlab bad feelings toh us waja se ap jo hai wo depress hojate hain, maybe, maybe that causes the depression. | Going through womanhood (giving birth) leads to depression  MD occurs during pregnancy  Distressing bodily and environmental changes can trigger depression |
| Interviewer: What is your understanding of the health problems that are usually faced by mothers during maternal depression? |  |
| Interviewee: Okay i wanted to clear my concept ke are we talking about the antenatal depression or the postpartum depression? |  |
| Interviewer: Maternal Depression overall. |  |
| Interviewee: Jee, theek hai. Can you repeat your question please? |  |
| Interviewer: So was asking what are the health problems that are faced by mothers during maternal depression? |  |
| Interviewee: Urmm basically health problems, ap ye puchna chah rhe ho ke what are the health problems that are causing depression to the patients? |  |
| Interviewer: It can be the problems jo unki waja se ho rhi hain or it can be the problems that occur after she has it. |  |
| Interviewee: Okay. Kuch health problems ho sakti hain patients ko jiski waja se they can go into depression like agar koi cheez smooth going hai aur wo out of the track ja rhi hai, koi complications patients ke sath hote hain jese ke postpartum patient ka wound kharab hojaye if they are having a c section ya koi bhi operation ho rha hai unka bleeding ho rhi hai ya jo bhi to agar unka wound kharab hojaye aur jaise ke i see the patients in the ward aur wo ooze krta rhe ke yar with burst abdomen, khula wa abdomen hai,  puss discharge ho rha hai to that is,  that bounds the patient to the bed, that causes pain and anything that causes pain will lead to depression. Patient recover nahi kar pate hain, ghar pe bache hote hain unke social issues bohot zada hote hain, patients apne ap ko trapped feel krti hain. Patients jab well being feel nahi kr rhi hongi they, they go to depression. Is ke ilawa antenatal depression ki agar baat ki jaye to antenatal ke bhi kuch issues ho skte hain jese ke take example of me, I'm having backache and I'm a working woman tou urmm, with urmm, with this kind of backache, severe backache and you're working,  you have to move all around,  you have to take rounds and all. Tou agar aap apni body urmmm bodily agar ap apne aap pe restrict hojayein and you have to do the work anyway, tou that causes,  not depression but that, that is bothersome for you. And eventually that may cause depression. | Patients with complications are more vulnerable  Anything that causes severe physical pain can lead to depression  Mothers feel trapped  Daily responsibilities add to mother’s burden, deteriorating her condition  Inability to continue daily routine due to physical symptoms can lead to feelings of helpless, then depression |
| Interviewer: So can you describe a case that left an impact on you? |  |
| Interviewee: Urrmm, not as such. I have seen cases but i haven't been in depth with them. Mene unko investigate nahi kiya ya meine properly follow nhi kiya but I've seen patients but i don't know what is,  what was the cause and what was the reason behind that and how they were treated and how did they recover. Tou i cannot give you an example particularly ke konsa case meine exactly pura, apko me bata sakun esa koi case. | Have not dealth with a case specifically |
| Interviewer: So urmm do you think maternal depression exists? |  |
| Interviewee: Uh, yes it does exist. Urm it does. Tou agar, Usually mene jo,  meine jo patients dekhe hain that comes with postpartum depression jab urmm delivery hojati hai, they are like, they have to go through sleepless nights. Ek jo labor pains, labor pains ka jo trauma hota hai that is, that is physical and mental both. Theek hai. Uske baad jab baby hojata hai usko sambhalne ka depression jo hota hai raat raat bhar, patient actually exhaust hoti hai. Usko urm, she needs rest. Kuch hospitals me nurseries hoti hain that take care of the baby, immediately after delivery, postpartum, kuch dino ke liye so that is a big help for the mothers. Tou unko relax krne me help krte hain. Lekin agar koi patient jese low socioeconomic background ke houn, jese government hospital set up like JPMC or any other city hospital ke, patients have to take care of the babies on their own. Unke sath koi support ho ya nahi ho to patient cannot recover through that phase, jo labor ka phase hota hai ya operation, agar operation huva wa hai to that is even worse than labor. Patient ki activity restrict huvi ho to patient ko feed karana hota hai to they have to take care of themselves and the babies as well. Tou ispo inko thora sa issue hota hai ke urmm, matlab wo well being feel nahi krtien hain aur unko irritation hoti hai ke kya mere pe museebat agayi hai aurr. Tou they feel kind of distressed. That can eventually become the depression | It exists  Labor pains can be a traumatic experience  Responsibility of a new child is not easy for a mother and can lead to depression  Lack of support in dealing with the new responsibility  Belonging to LES and not having sufficient help  Restrictions due to caesarean deliveries can result in MD  Mother needs to adapt to a completely new life style which may cause distress and eventually depression  Sleepless nights |
| Interviewer: And do you think this adds to the burden in the overall society that we live in? |  |
| Interviewee: Yes, it does but people should help. The family members,  they should understand, obviously they should, they should urm help the patient recovering and they should understand the condition ke har koi aurat pe hota hi hai, har kisi ne bache paida kiye we hote hi hain. Touu sabko understand karna chahiye,  even the hospital should take care of a, the patient, really well,  empathy show krni chahiye, to families. |  |
| Interviewer: What do you understand by prenatal and postnatal depression? |  |
| Interviewee: Urmm prenatal depression mene apko jese bataya ke it is because of the condition that is the, that is going on urmm urm antenataly. Koi bhi condition hai patient agar uske sath koi bhi complication hai agar blood pressure high hai to patient ka at peace aur urm agar sugar issue hai to she's actually concerned about the pregnancy, the outcome. Usko wo depress krta hai ke mere sath esa sa masla hai, how is the baby going to survive,  what is going to be the outcome of the baby, urmm kya hoga, kya hoga mera baby bachega nahi bachega ya meri pregnancy kis tarah ki chalegi, tou that may cause,  urm the depression,  maybe. If the patient is overthinking, kuch patients sensitive hote hain, actually kuch patients ka, urmm kuch logon ka andar built in cheez hoti hai ke they are sensitive and they overthink. Tou agar kisi patient ke sath koi bhi issue hota hai to they keep on thinking about it and urmm they don't take it strongly or if they, if they, if the people around,  they do not support, unko antenatal. Kuch husbands hote wo unko bolte hain ke ap khud jayein aur khud apna antenatal check up karayein ya jo bhi, if they are not supportive ke patient ko le ke na jayein, unke sath support na hou, tou isliye.  Tou us waja se jo hai unka urm, uska depression jo hai us waja se bhi ho skta hai, antenataly. If the patient is in pain, antenataly aur kisi bhi tarah ka distress hai unko,  physical ya mental, unko ghar me bohot kaam krna par rha hai, they need rest but they cannot rest, take rest, tou us waja se bhi unka depression ho skta hai ke mera is time pe sab logo ko khayal krna chahiye aur, because when a person is pregnant, they expect ke the society and the people around them and the doctor and every, everyone is going to take care of them really well kyunke wo thora special feel krti hain us waqt, Tou agar na ho to phir that can cause depression. Postanatal, postnatal jis tarah mene apko bataya ke if the patient is going through any sever condition ya koi bhi esa issue to uspe unko depression ho skta hai, koi complication hojaye to uspe unko depression ho skta hai, iske ilawa if the labor has gone,  not gone well, urm patient mentally aur physically traumatized feel krti hain to us se bhi depression ho skta hai. | Antenatal and postnatal depression can occur because of a complicated pregnancy  Continuous stress of the process of pregnancy may lead to depression  Mothers who overthink and are sensitive  Lack of support to deal with the process from husbands  Excesssive work burden and no rest  Mothers feel entitled to care when pregnant. Lack of love and care can result in depression  Postnatal depression can occur if labor doesnot go well and outcome is not favorable |
| Interviewer: Urmm Symptoms kya hote hain maternal depression ke? |  |
| Interviewee: Symptoms,  urmm, as i told you that i haven't seen much patients about,  urm matlab this category but i think, urmm if I'm talking about myself,  i have been urmm a,  i am a mother actually and I'm expecting a second baby In Sha Allah. Tou jab meri delivery huvi thi, I was urm, during labor i was in very much pain. Tou jab mein, delivery krdiya tha mene tou us waqt mein, i was in a kind of, in a,  in a state of shock kind of ke mere sath huva kya hai. Tou it was kind of urm, traumatizing, for me. Tou it was not lingering on, it didn't,  i didn't go into depression but i overcome urm,  the situation,  but us waqt jab ab bohot zada ek cheez se guzarte ho, jese just take the example of people who are in jail, like Pakistan ke border pe kisi ne India ke wale ko pakarliya, us ko torture kiya aur they, you know psychotic patient hojate hain us tarah se, unko mental ya physical trauma, agar ap kisi ko dein baar baar. Tou it was kind of that because it is a very big experience,  ek bohot bara milestone hota hai us waqt baby ko paida karna because patient goes into very much pain, tou that is a big thing, that can cause mental distress. | State of shock after delivery can be traumatizing and can result in excessive distress  Mother feels helpless and tortured. She feels that there is no way out |
| Interviewer: Tou what do you think is the time duration for prenatal and postpartum depression? |  |
| Interviewee: Urrm, no i don't know exactly, what is that. Maybe urmm, it, it can happen anytime. Urm Most, urmm mostly,  urm matlab exactly jab period apka forun se off hota hai kisi cheez ka tou thore time ke liye, maybe for few weeks, immediate weeks jo hote hain that is, that are most important. | MD can occur anytime  Immediate weeks are high risk periods |
| Interviewer: Jab ap prenatal depression ki baat krti hain to wo konsa time frame hai jispe ap focus kr rhi hain? |  |
| Interviewee: Urmm prenatal depression can start anytime, jab bhi patient jo hain wo symptoms apne ya jo bhi,  jo bhi koi factor jo stimulus hai jo unko, jo agar karega us cheez ko, kabhi bhi a skta, during anytime. | Prenatal can occur anytime during pregnancy |
| Interviewer: After conception or before conception of the baby? |  |
| Interviewee: Urm After conception. If we are talking about maternal depression, then it should happen after conception. | MD occurs after conception of baby at any time |
| Interviewer: Jee,  theek hai. And post partum jo hai wo forun ayega ya ruk ke ayega? |  |
| Interviewee: Forun bhi a skta hai, ruk ke bhi a skta hai. It depends upon the condition of the patient and how she tackles. | Postpartum depression can occur either immediately or after a few weeks after pregnancy |
| Interviewer: Among mothers who is most at-risk for developing maternal depression? |  |
| Interviewee: Urm I think the people who are of …., ek to risk factor patient ka apna bhi hota hai, ke patient ke apne andar, if she's sensitive,  if she is that kind of personality ke wo overthinking krti hai bohot zada ya sensitive hai, cheezoun ko bohot zada apne uper hawi krleti hai, choti choti cheezoun ko wo bhi. Tou she's prone to depression. Theek hai. Uske uper koi bhi cheez ayegi, choti cheez bhi ayegi to she'll take it as a big huge, mountain, type, hojayega usko ese. Ek tou ye baat hai uper, uske baat dusri, the stimulus that is creating the depression, theek hai, wo cheez ho skti hai. Tou ye cheezein hain. There are different factors | Patients who are sensitive and overthink  There are different factors  Certain stimulus are needed to trigger depression |
| Interviewer: Urm how can, urmm can you talk about any other factors. You spoke about, urm being over sensitive. What other factors do you think will put a mother at risk? |  |
| Interviewee: Urmm low socioeconomic background. Theek hai. If the people are not caring because of the financial issues maybe or agar people around her, if they are not caring jese hota hai ke, urmm, in our part of society, jese urm, in laws me reh rahe hain tou itna zada care nahi hoti. Usually hamare paas ye trend hota hai ke when the, when the patient has delivered, she goes to her mother's house. Tou isliye agar shayad wo waha pe reh rhi hain tou unko itna care nahi mile aur she, she thinks ke she is not taken care well. Tou uspe unko ho skta hai, this can cause the depression, and urmm what else. Yehi cheezein hain i think. | Low SES  Lack of care due to financial constraints  Women are taken better care of at their mother’s house than their inlaws - Lack of care from family |
| Interviewer: So how can we manage maternal depression? |  |
| Interviewee: Urmm, basically we have to be empathetic and sympathetic to the patient. Unko thora special feel krana chahiye, unki, unka khaas khayal rakhna chahiye.  Especially i think the husband and the immediate family member….The immediate family, they should take care of the patient, well. Agar kisi ko lagta bhi hai ke patient depression me ja rhi hai ya is tarah ka kuch hai tou they should urm understand that and if, urmm, in need, they should take the patient to the doctor, the psychiatrist, take cheezein jaldi resolve hojayein aur jis waja se,  urm, patient ko depression ho rha hai, they should urmm, wipe it out. | Empathy and sympathy  Patient needs to feel special  Mother expects husband and immediate family members to take care of her – Lack of care results in depression  Family should immediately consult a doctor if they think the mother is suffering from MD |
| ---PAUSE--- |  |
| Interviewer: So urmm, doctor sahiba what are the short term and long term implications of maternal depression? |  |
| Interviewee: Urmm short term implication yehi ho skta hai ke patient, if the patient is depressed tou jo bhi depression ke symptoms hote hain ke ap apna khayal karna bhul jatey ho,  apke sath jo bhi log hain ap unka khayal karna bhul jate ho. You do not take, you do not, you're carefree actually. Carefree bhi nahi kaheinge, use kaheinge ke you are negligent, you become negligent to the family, to your baby because iske baad sabse zada care ek ap ko hai, aur ek apke baby ko hai. Tou ap apni health compromise kroge, ap baby ki health compromise kro ge, theek hai. Tou ap kisi pe burdenize hojaoge ke ap uske uper bojh banjaoge ke ye ab hamara khayal rakheinge aur matlab, indirectly ap ye kr rhe hote ho. Touu urmm ye ye issues ho skte hain, because of the depression these are the issues. | Lack of self-care  Inability to care for others  Negligence towards family and baby  Mother compromises on her own health  Baby’s health is compromised  Burden on caregivers to take additional care of mother and baby |
| Interviewer: Mother pe personally kya effect hota hai short term aur long term me? |  |
| Interviewee: Mother pe urmm, i think mother, urmm, ke uper itna zada effect nahi ata hai. Mother, matlab agar depressed hai tou,  mene jese bataya ke wo apna khayal nahi rakhegi, khane peene ka khayal nahi rakhegi because postpartum ya antenataly bhi agar ao dekhein to mother ko nutrition ki bohot zarurat hoti hai, she needs rest, she needs, needs good diet urm to take care of the baby, ek baby andar pal rha hota hai,  usko agar, if they want the baby urm, if they want the baby to be born healthy and to stay healthy after urmm birth to unko achi diet rakhbi hoti hai. They should take care, good care of the diet and urmm they should take care of it, of their rest timing hours. Tou agar wo nahi kar pati hain, raat ko jaag rhi hain,  sochti ja rhi hain ulta seedha, ya khana nahi kha rhi hain because in depression you lost your appetite,  Usually. Tou this can cause matlab.. | Mother is not affected to extreme levels  Lack of self-care  Doesnot look after her diet which is needed to ensure safety of mother while carrying another life  Disturbed appetite can harm the unborn baby  Disturbed sleep cycle |
| ----PAUSE----- |  |
| Interviewer: So we were talking about the short-term and long term implications, so you were telling me about how a mother is not that affected but maybe the family and the child is so can you just….. |  |
| Interviewee: Not the family as such, the baby will be affected. If the baby is not taken care well, by the mother tou aur agar usko family me koi support nahi hai aur us tarah se agar dekha jaye to baby suffer karega. Because agar mother, urm meine, urm, I have seen maybe, urm, keh sakte hain ke if the mother is depressive throughout her pregnancy, tou wo kehte hain ke baby pe bhi asraat ate hain. Aur agar baby, us waqt to nahi, bache to bache hote hain, chote hote hain to wo, that is urm, they do not show the element of depression but wo jab bare hote hain tou, there are a few researches as well, ke jab wo bache bare hote hain, they show, urm, in their lives ke they have this urm, urmm this sense of depression in them. Tou wo depressed feel karte hain. Unke andar element hota hai yeh. | Family is affected to a certain extent  Baby is affected the most  The baby is not well cared for  If mother is depressed during pregnancy, the child may end up being depressed when he/she grows up |
| Interviewer: Theek hai, tou family members affect bilkul bhi nhi honge ya hounge? |  |
| Interviewee: Nahi, family members bilkul affect honge. Ek family member, apki puri family me, agar ek family member bhi theek na ho to puri family affect hoti hai obviously. | Family will be affected if any member is sick |
| Interviewer: Theek hai. |  |
| Interviewee: Tou agar patient, mother ko depression hai ya mother ke, kyunke ek ye cheez hoti hai ke ek bara event hota hai, the,  the baby has arrived in a family,  a new family member has been added. Tou agar wo cheezein theek se na jayein kyunke sab log ghar me khush hote hain, agar uske sath koi complications houn, koi masla ho, tou puri family affect hoti hai obviously,  because mother, urm, urmm jo hoti hai usko kisi na kisi ke khayal ki zarurat hoti hai, she cannot do everything by her own, tou uske sath jo log affiliate hote hain aur phir uske sath bound hojate hain wo apne kaam chordete hain tou obviously wo sara pura ghar ka set up disturb hota hai, ek cheez ki waja se. | The family is unable to celebrate the baby’s birth if mother is unwell  Family is bound by the mothers’ condition and are unable to complete their own daily tasks  The house environment remains disturbed |
| Interviewer: Theek hai. Aur apko lagta hai ke jitne bhi associated caregivers hote hain ya partners hote hain, jese ke apke husband hogaye, unko uper koi affect hota hai? |  |
| Interviewee: Unke uper bilkul affect hota hai. If they are caring and loving, and they care about you, tou unke uper bilkul affect hota hai because if you in pain, they feel pain, the pain as well. | If husband and family is loving and caring they are negatively affected |
| Interviewer: Theek hai |  |
| Interviewee: Tou agar wo concerned hai truly about yourself and if they are loving,  aur apke sath bilkul sincere hain to obviously they are going to feel,  go through the same pain that you are going through. | Family feels the pain of the mother if they are sincere and supportive |
| Interviewer: So in your opinion how is maternal depression managed in Pakistan? You claim that you know it's there, it's very well prevalent. |  |
| Interviewee: Haan it is there,it is prevalent because, we urmm, are accounted in third world country and we are not very socioeconomically strong, tou yaha pe zada tar society jo hai wo isi type ki hai ke wo low social economic background se belong karti hai. People have urm, do not have the awareness. Pehli baat to ye ke unko awareness nahi hai aur wo jo cheezein ho rhi hoti hain, they are like kyun ro rhi ho, kyun rona dhona daala wa hai, khayal karo bache ka. Uper se log samajhne ke bajaye unko aur daant dapat karte hain, aur is tarah se. Abhi me round krke ayi hun ward me se, ek patient,  she was crying.  Tou sab log usko thora,  urmm, sympathy show kr rhe they. Keh rhe they, puch rhe they kyu ro rhi ho, kya hai, lekin at the end of the day, they are going to neglect that. Ke tmhe kyu is tarah se huva, this is not normal. You should be, you should be treated well, ya urm they are not going to urm, go into the roots of that ke apke sath esa kyu ho rha hai, ap kyu ro rhe ho basically.  Ye baat ayi aur baat chali jayegi ke wo ro rhi thi bas. | It exists  It is prevalent  Low SES contributes to depression  Lack of awareness  Family scolds the mother and neglects her when her functionality is disturbed  Family doesnot focus on the root of the problem but rather wants the mother to fix herself |
| Interviewee: So do you think hospitals diagnose kartai hain maternal depression ko ya tools use hote hain? |  |
| Interviewer: Urmm, the hospitals who are, urmm following the protocols,  urmm jaha pe actually jab, urmm, basically jo government hospitals hain they are very much overloaded with work, unko, un, i think wo ye kaam krte hain ke apko agar sare mareezoun ko basic care hi provide kar payein to wo unko bohot bara achievement hota hai. Jese in our set up at JPMC agar wo basic support, proper sterilization, proper antenatal care jo hoti hai,  not the depression,  not the psychiatric issues, jo patient ki bodily sari cheezein hoti hain ya antenatal care aur sab kuch patient ko, urm, labs manage karna ya patient ke jo bhi issues hain, basic issues, solve krlein to that is a very big achievement for them. Because yaha pe patient ka load itna zada hota hai aur patients yaha pe bohot zada,  urm, jis quantity me a rhe hote hain, tou un sabko, urmm, sari cheezein unki, choti choti cheezein manage krna aur unko better pregnancy outcome dena, urmm wo unka bara achievement hai but if we're talking about bare hospitals, maybe private hospitals, aur jaha pe, urmm patient ki ek ek cheez jo international standards pe kaam krte hain, unke liye urmm, wo log shayad manage krte hain and they go through it, they evaluate things with urm, this perspective. | Government hospitals are overloaded with work  It is an achievement is Government hospitals are able to provide basic care to all mothers  Treatmnet for MD is not part of basic care needed for mother  Antenatal physical issues are more important than the emotional issues  International standards are followed by private hospitals and so depression can be evaluated there |
| Interviewer: So do you think that, urm maternal depression is part of the initial screening process that a patient goes through? |  |
| Interviewee: It should be, but not for everyone. Urmm, actually jab patient ke paas, urm doctor ke paas patient ata hai, wo patient urmm, pehle dusre visit me hi pata chaljata hai ke what is the kind urm, what is,  what the person, what urm, what personality does the patient have. Tou jese mene bataya ke patient,  kuch patients ki personality esi hoti hai ke ap us se cheez, matlab dekhlete ho ke the patient is sensitive ya, patient is overthinking,  wo agar baby ke bare me baar baar puchraha hai, acha mera baby theek hai na, ache meri labs theek hai na, baar baar patient ke sath, urm baar baar visits kr rha hai,  ya baar baar doctor ko bother kr rha hai, ek cheez baar baar puche ja rha hai, tou that means patient is very much, urmm, urm overthinking about her pregnancy and she's taking pregnancy very very seriously, tou doctor should urmm, treat the patient, urmm usi tarah se jis tarah patient jo hai wo chah rha hai. | Screening for depression should be provided  Currently patient is not counseled well when she visits the doctor  Sensitive patients are difficult to deal with and due to overcrowding they are not counseled appropriately |
| Interviewer: Theek hai. So am I right in assuming ke koi screening nahi hoti fil waqt? |  |
| Interviewee: Ah Nahi. Koi screening as such nahi hoti hai. I haven't seen, urm, anybody doing such screening ya hamein at student level bataya gaya ho ke urmm patient aye to apko urm, we know ke we have to do the baseline, ye ye test karane hain, blood test karane hain aur ultrasound karana hai and we have to deal with the patient in that way. But hame I think ye nahi bataya gaya ke patient ki jo hai depression ko bhi rule out krna hota hai patient can be, can go into depression. Ye cheez dekhni hoti hai, we have to screen tha patient in this, this aspect. | No screenings are done  Students are not taught to rule out depression while screening the patient for pregnancy related issues |
| Interviewer: Urmm and I, am I also right in assuming ke apke hisaab se har patient ko screening ki need nahi hai lekin ek do visits ke baad patient ko dekh ke ap screen karo agar need hai tou? |  |
| Interviewee: Urmm, I do not actually know the criteria how do we screen the patients, i do not know the ways how do we screen the patient lekin i think agar patient, agar doctor apna ye reflexes banaye ke patient se jab mil rha hai wo baat kr rha hai pehle dusre visit me, tou she should know ke how the patient, urm in the antenatal, urmm how is the patient going to be in her pregnancy. Ya ap jab antenatal visit kr rhe hote ho, you can go through the personality of the patient, through their visits. | No knowledge about screening criteria  No understanding of the process of screening  Doctor can maybe assess the patient’s personality during her pregnancy visits to help with diagnosis |
| Interviewer: Kya apne apne experience me kabhi bhi screening tool istemal kiya hai ya dekha? |  |
| Interviewee: Urm no, no. Kabhi suna bhi nahi. | Never heard of a screening tool |
| Interviewer: Acha. Do you think that maternal depression can be treated or managed? |  |
| Interviewee: Yes it can be treated,  urmm, bilkul treatment ho skta hai, maternal depression agar wo postpartum hota hai to that is written in our books as postpartum blues Tou that urmm, thay patient needs a psychiatric treatment. Wo worse bhi ho skta hai,  that can lead to postpartum psychosis as well jisme mother apne ap ko harm kar skti hai, baby ko harm kar sakti hai. Tou ye cheezein treat ho skti hain obviously and she needs proper care for that aur isko identify, urmm ye cheezein krni chahiyein aur patient should be referred to the medical practitioner ya jo bhi psychiatriat related hai is cheez se so she should be treated to that aspect. | It can be treated  Postpartum depression is termed as postpartum blues  If not taken care of postpartum depression can lead to psychosis  Mother can harm baby in psychosis  Patient should be referred to a psychiatrist immeditaly if diagnosed with MD |
| Interviewer: Konsi different kisam ki treatments hain apke hisaab se is waqt for maternal depression? |  |
| Interviewee: Urmm, no idea. I have no idea for this. Maybe some,  maybe some antidepressant tablets waghera ya jo bhi cheezein hain. Lifestyle modifications. | No information regarding the therapies  Antidepressants and lifestyle modifications |
| Interviewer: If effective treatments do exist, jese apne kaha hoti hain, right. Tou kya mothers aur families help lene jati hain? |  |
| Interviewee: Urmm,  mostly in our society,  no. Because identify hi nahi hota hai tou obviously help ka koi sawal hi nahi hota baad me bhi. Bohot hi kam percentage, bohot hi, bohot hi, bohot hi minority me hoti hogi ya jo patient, jo pay, jo log, jese mein, i am a doctor, tou maybe if something wrong is going on in my family tou i can diagnose that, that this patient is,  that urm, this family member of mine is not well, postpartum,  tou i can diagnose this. I can, urm, council them and refer them to, urm, a medical practitioner or, urmm, a psychiatrist for that. Tou bohot hi kam hota hai ke log pehle identify karein because pehla step tou identification hota hai ke identify kar skein. Uske baad phir treatment ka sawal ata hai ke patient ko manaya jaye aur uske andar ye insight ho ke mein theek nahi hun, i need help. Basically yehi Cheez hoti hai ke patient ko khud manaya jaye ke, ya khud maney patient, is Cheez ko accept kre ke I'm not well. Tou, urmm,  is tarah | No effective treatments exist in our society  Helping the mother is impossible since the condition goes undiagnosed  Very rarely is MD diagnosed  Treatment can only help if identification channels are properly followed  Mothers have no insight about their condition and so cannot seek help  Mothers deny their condition  Only minority is fortunate enough to be diagnosed |
| Interviewer: So, urmm, aisi kya approaches hain ya combinations of approaches hain ke jinkai through ham jo hai mothers ko engage kar skein jo ke depressed hain? |  |
| Interviewee: Engage in what sense? |  |
| Interviewer: You can get them to, urmm, unko ap wapis se productive bana sakte ho for the society? |  |
| Interviewee: Urmm basically, urmm, it depends upon the degree of depression they are having,  agar bohot minor sa depression hai, negligible type ka tou agar unko routine me busy kiya jaye, unke sath matlab,  urmm, log mile julein, hasein bolein aur urmm, unko agar acha well being feel karaya jaye aur unko special feel karaya jaye ke this is the baby that you have given to our baby and this is very precious and we are, we're very happy with this,  koi choti party ya kuch bhi arrange hota hai jis tarah se aur, urmm,  patient ko matlab relax kiya jata hai, unka acha khana peeney ka khayal rakha jata hai aur sab kuch. Patient ko basically, urmm, at home feel karaya jaye bohot ache tareeke se ke apki hospitality ho rhi hai aur ap is tarah se, tou obviously that this will help. Agar arrr, higher degree ka depression hai,  tou patient should be,  urmm, taken to.the doctor. | Minor depression can be cured through distarctions such as family time  Mild depression can be cured by making the mother feel special, relaxing her, providing her care and a healthy diet.  Mothers suffering from severe depression need to visit the doctor |
| Interviewer: Theek hai. So apki kya perceptions hain ke what are the barriers of seeking care for such a crucial issue. Why do women not seek care? |  |
| Interviewee: Because firstly, they're not identified and secondly, urmm, they are ignorant. And urmm, Agar, kyunke, because this is a big taboo ke agar ap psychiatrist ke paas jate ho hamari society me, ke is tarah se jo masla hai tou mein pagal,  doctor ke paas ja rhi hu ya mein pagal hogayi hun ya you know the people around you, they urm, they'll think ke. Patient ye soch rha hota hai ke people around me, they'll think ke mein theek nahi hu aur mere sath yeh stigma attach hone ja rha hai ke me psychiatrist ke paas ja rhi hu is tarah se. Tou ye, this,  his bounds the patients back to their home. Tou they do not go. | Identification remains an issue due to lack of awareness  Ignorance and social taboo  Mental health is stigmatized  Fear of being the society’s talk |
| Interviewer: Theek hai. |  |
| Interviewee: This could be the reason. |  |
| Interviewer: And what do you think cultural aspects kya hote hain? Jiski waja se patients ke sath ye issue ata hai? |  |
| Interviewee: Urmm, matlab cultural aspects se? |  |
| Interviewer: Urmm, you think hamare culture ka koi role hain in a mother being depressed? |  |
| Interviewee: No urmm, i don't think so. | Culture plays no role |
| Interviewer: Do you think ke religious beliefs ki waja se mother seek nahi karegi care? If she's depressed? |  |
| Interviewee: Urmm, no. Agar if she has the insight,  she knows I'm not well, i need treatment. Because ye cheez jo hai patient ke andar khudse ani chahiye ke, because religiously tou, urmm,  medical treatment lene me tou koi harj nahi hai. Na hi ham esa sochte hain yaha pe Pakistan mein ke, matlab ke agar, ke agar, urm,  mareez ko khud pata hona chahiye ke I'm not well. Agar wo theek nahi hai tou she should be going to the doctor. | No religious factors  Mother should be able to identify her problem for treatment to take place |
| Interviewer: Theek hai. Aur apko lagta hai ke supply side ki waja se koi issue hai? |  |
| Interviewee: What supply side? |  |
| Interviewer: Ke providers hamare paas kya sufficient hain ke hame dena ho, care seek krni bhi ho kisi ko tou do we have enough providers ya? |  |
| Interviewee: Nahi, we do not have specialized people who are treating the maternal depression exclusively.  Urmm, generally ek psychiatrist hota hai, pehle to ye cheez identify karta hai i think the doctor,  the gynecologist you are, urm, urm, doing your visits with. Uske baad they refer to the psychologist or the psychiatrist. Tou generally wo treat kr.rhe hote hain, they are not specialized for that, but i think they treat the people nicely. They can treat the people. | Lack of specialists for MD  All psychiatrists and psychologist cannot treat MD  A gynecologist is responsible for first identifying it |
| Interviewer: Theek.hai. So how do patients perceive maternal depression?  Jo khud apne aap patient hote hain, unko kya feel ho rha hota hai us waqt? |  |
| Interviewee: Urmm, maternal depression is not urm, a separate from the other kind of depression. Depression hai to wo tou depression ek hi tarah ka feel hoga,  i think ke the patient will feel low, baat krne ka dil nahi krega, bhook nahi lagegi,  urmm bache ka khayal karne ka dil nahi krega, urm, kaheen ane jane ka dil nahi krega, ya tou neend ayegi, ya tou nahi ayegi. Tou the symptoms are the same, i think. For all kinds of depressions. | All kinds of depression have the same symptoms  Feeling low  No motivation to interact with people  Lack of motivation to look after the child  Disturbed sleep  Isolation |
| Interviewer: Sahi. And what role do you think the medical framework of Pakistan plays in management of depression? |  |
| Interviewee: None. | Medical framework doesnot play a role in creating awareness |
| Interviewer: Matlab kya apke level of diagnosis apko sikhaya jata hai? |  |
| Interviewee: Urmm, No. General ek rule hai ke how do we urmm, urmm,  diagnose and treat depression, but not exactly ke maternal depression ko kis tarah, urm parha jata hai. Maybe i am not at this level ke meine bohot zada books parhi hain, maybe hoga but i haven't been through up to my level. | No knowledge is provided in curriculum pertaining to MD in early years of studies |
| Interviewer: Nursing ke ya apke medical curriculum me lagta hai apko ke included hota hai yeh? |  |
| Interviewee: Urmm, at the MBBS level? NO. Depression as general hai, but not the maternal depression, exclusively. | Not included at the MBBS level |
| Interviewer: Job pe kabhi koi training hui jab apne start ki ho tou? |  |
| Interviewee: Not at all. | No on job trainings |
| Interviewer: Koi workshops ya trainings jisme apko? |  |
| Interviewee: Urmm, i remember one i think but not exactly,  but workshops hoti hain i think,  yes hoti hain. Workshops nahi, maybe you can say lectures ya one day jo CME lectures hote hain, seminars.  Yes. |  |
| Interviewer: Tou kya ap kabhi unka hissa bani hain? |  |
| Interviewee: Urmm, i think once. |  |
| Interviewer: Kya mandatory hota hai ya nahi? |  |
| Interviewee: Nahi, nahi.  That's not mandatory. That is up to you if you want to take that. You are welcome,  you are invited | No mandatory workshops |
| Interviewer; So only the doctors who want to take it will ever have access to it? |  |
| Interviewee: Exactly.  Yes. | Only doctors are invited to workshops |
| Interviewer: Practical experience me kabhi kaam ayi ye cheez ya khud notice ke yar maternal depression ho rha mother ko? |  |
| Interviewee: Haan we all, i can, i, as a gynecologist, all i can do is, identity the cause,  council the patient, and be empathetic to the patient and urmm, tell the patient she is suffering from something. Patient ko ye razi karana ke you need help, you should go to the doctor. Tou bas we can do this. | Gynecologist can counsel the patient to visit a psychiatrist or psychologist  Gynecologist can help the patient understand her own condition |
| Interviewer: Tou apko lagta hai ke self-efficacy jo hai wo diagnosis aur management pe effect krti hai? |  |
| Interviewee: Self-efficacy matlab? |  |
| Interviewer: Matlab apki apni, how, how comfortable are you telling the patient about it ya how much responsibility,  ap kitne khud, matlab tayar ho. |  |
| Interviewee: As a gynecologist,  we should,  urm, treat the patient with every aspect because health ki definition hi yehi hai ke ap patient ki mental, physical,  social, well being matlab,  ek, kisi bhi insan ki mental, social aur physical well being ko healthy person kehte hain. Tou agar patient ko agar,  urm, if the patient is healthy, physically and not mentally,  then the patient is not fine, you have to treay the patient mentally as well. You have to make the patient well mentally too. Tou apko identify karna chahiye being a good doctor apko khud ye cheezein identify kar ke patient ko treat karna chahiye. With this aspect too. | Gynecologist should treat patient in all aspects – physical, social and psychological  Definition of health taught to doctors focuses not just on physical wellbeing but also emotional health  A good doctor is able to diagnose MD |
| Interviewer: Apko lagta hai ke gynecologist ki, apki personal responsibility hai patient ki taraf ke usko ap maternal depression ke liye screen karo? |  |
| Interviewee: Jee bilkul hai. Honi chahiye. | Gynecologist is responsible for all aspects of her patients life |
| Interviewer: Do you think that compassion or empathy ka isme koi role hota hai? |  |
| Interviewee: Bilkul hai, urmm, doctors ko, urmm mere walid sahab kehte hain ke patient agar doctor ke pas jata hai to doctor ke rawaiye se hi uski adhi bemari theek hojani chahiye.  Tou patient, doctor ka rawayya is tarah ka hona chahiye ke patient uske sath bohot acha feel kre aur wo uski batein maney aur urmm jo patient ki counselling doctor karta hai wo bohot bara help hoti hai depression ko even treat karne mein bhi. Tou agar patient depressed hai aur patient ki counseling doctor bohot ache se kar rha hai, empathetic ho ke kr rha hai aur patient ko wo cheezein dimagh pe lag rhi hain ja ke aur wo accept kr rha ha in cheezoun ko tou that is a very big help for the patient. | Half of the cure to a patient’s problems lies in the doctors attitude  A mother will listen to a doctor who treats her well  Counseling can only be successful if the mother is able to bond with the doctor  Doctors need to be empathetic |
| Interviewer: Theek hai. Apko lagta hai ke comfort ya confidence play a role in this? Will you be comfortable talking to a patient about maternal depression? |  |
| Interviewee: Yes obviously. Own the patient, if I urmm I'm seeing the patient, and if I urmm I urmm if I urmm, main agar patients ko own kar rahy hun aur urmm main chahty hun keh meray patient theek ho keh jayen meray paas se toh i woulf like to cover all the aspects. Keh patient mera har tarhan se theek ho, mentally bhi physically bhi, aur sab tarhan se | Ownership of the patient helps the doctor treat the patient in the best possible manner |
| Interviewer: Theek hai |  |
| Interviewee: Toh i would definitely put an effort |  |
| Interviewer: Do you think infrequent followups ki waja se weak realtionship hoty hai? |  |
| Interviewee: Bilkul hota hai. Patient bhi jo hai follow up nhi lerha hota hai, patient keh sath bhi bohat issues hotay hain. Doctor bhi thora sa gussay main ata hai keh patient why is patient not following up. Jab emergency horhy hai ya masla horha patient sari cheezen hamaray pas kharab kar keh larha hai. That they're coming to us aur phir they want us to treat, and treat them well. Toh thoray se issues hotay hain, barriers hotay hain jiski waja se cheezen khatab hoty hain | Follow-up remains an issue  Patients come when condition is severe |
| Interviewer: Okay, apko lagta hai patient agar gynecologist keh sath proper follow up rakhay, toh gynecologist apnay end se uska maternal depression treat karnay keh lye kuch kar sakty hai? |  |
| Interviewee: Urmmm maternal depression treat karnay keh lye, if it, if it is diagnosed at a very low point, to shayad koshish ki jaskty hai with the counseling because depression can be treated with counseling if it is not going to the full blown stage. Toh counseling keh sath, aur cheezon ki help keh sath, uski family ki counseling keh sath uski immediate partners ya caregivers ki counseling keh sath ye cheez treat hoskty hai at a very early point aur isko progression ko roka jaskta hai. Liken agar bohat zada cheez barhgyi ho, patient jese keh infrequent follow ups kar raha ho bohat kharab agar depression keh sath arha ho toh the patient should be referred to a specialist. | Mild depression can be treated by counseling the mother, the family and all caregivers  Severe cases can only be handled by specialists |
| Interviewer: Theek hai. Kia general practioners should be the first line of support for the patient? |  |
| Interviewee: Urmmm general practioners jo hain wo har kisi ka first line hotay hain I think, unkooo unkay paas itni abilities hoty hain aur I think unko they are, they have this JD keh unko choti choti cheezon ko identify kar keh urmm jab tk iss trhan na ho keh patient full blown, kharab stage main doctor keh pas aaye, toh they should identify the cause at a very early stage and should try to treat it. If they're not urmmm not treating it properly, ya toh agar na kar payen they have not tendancy to treat it then they should refer it to the specialist. Toh general practioner ka bohat bara role hai keh isko early identify karen aur isko sahi jaga refer karen | GP should be able to diagnose patient at a very early stage  GPs houdl refer the patient immediately if they are unable to teat the patient  GPs play a critical role in early identification |
| Interviewer: Gynecologist apnay end se kia kar sakte hain? |  |
| Interviewee: Urmmm gynecologist apnay end se ye kar sakty hain keh if the patient has, is having frequent follow ups, aur patient doctor ki bonding achi hai toh patient jo hai wo counsel hoskta hai by doctors aur treat hoskta hai aur jese hi doctor ko lagta hai the things are not in her or his hands the patient can be referred. | If patient doctor bond is strong and patient frequently visist, gynecologist can counsel the patient  Gynecologist should refer the patient instantly if she is unable to treat the patient |
| Interviewer: Theek hai. So what factors affect in the diagnosis and management of maternal depression in a hospital setting? |  |
| Interviewee: Sorry can you repeat the question? |  |
| Interviewer: Diagnosis ya managment of maternal depression aik hospital setup main kyun nhi hota? |  |
| Interviewee: Urmmm aisa nhi hai keh nhi hota, kuch hospitals main hota hai kuch hospitals main nhi hota. Jese mainay apko bataya jahan pe patients ka load bohat zada hai, keh we cannot cover all the aspects, urmm saray health aspects jo hai hum nhi cover kar saktay, ya patients urmm doctors bihat over burden hotay hain ya iss trhan ka toh basic health care hi provide kardi jaye physically, aur unko theek se rakha jaye aur agar wo theek rehtay hain physically maybe, not with the mental aspect kyunkay wo baad main cheez aty rehty hain hamari society main keh unko baad main dekha jata hai baad main treat kia jata hai. Toh ye cheezen agar hojayen to most of the societies comprises of this low, low soci economic background aur urmm saray log government hospitals ka hi wo kartay hain toh we are kind of over burdened so we cannot cover all the aspects | Only some hospitals are equipped to diagnose and manage MD  Government hopsitals are overloaded  More importance is given to physical aspects |
| Interviewer: So do you think keh hamara jo basic screening form hota hai gyne ka usme koi mental health ka aspect hai patients se poochnay k lie? |  |
| Interviewee: No, not at all | History form entails no questions pertaining to mental health |
| Interviewer: Koi proactive monitoring tools? |  |
| Interviewee: No | No proactive tools |
| Interviewer: Theek hai, referals? |  |
| Interviewee: Urmmm i havent seen a single referal in my two years of experience urmm maybe once case urmm I saw at AKU keh wahan pe aik patient thy urmm she had post partum psychosis toh wo she was trying to urmmm she used to urmmm roam around in the ward, aur wo chila rhy hoty thy apnay husband se lar rhy hoty keh mjhy bacha nhi chaiye aur ye bacha aisa hai aur iss trhan ka. I think she was in post partym psychosis aur she was referred to a psychiatrist. She didnt want any treatment because she didnt have an insight that main beemar hun ya meray sath koi masla hai | No referral encountered in two years of work  Due to lack of insight, mothers donot want to be treated  MD may make a mother hate her own child |
| Interviewer: Sahi. So kia factors hain jo help kar saktay hain mothers ko protect karnay main? From maternal depression? |  |
| Interviewee: Urmm, unki achi si care ki jaye, doctors agar iss cheez ko identify karlen shuru se keh the patient has the tendancy of being into depression, afterwards unki family ko counsel kia jaye keh the patient is, urmm is going through this pregnancy and they need this kind of care urmm because they are special to you urmm because they're going to urmm give a new family member to the urm family aur urm thora sa special ya thora sa urmm different urmm rawaya agar patient keh sath rakha jaye during pregnancy, maybe aur khush rakha jaye basically unko toh urmm shayad depression main na jayen. But people have the tendancy to go in the depression shayad agar acha bhi feel karaya jaye sab ho bhi unkay sath, if they're sensitive, and they're prone to depression, they can go in to depression any time | Care  Family counseling  Early identification  Making the mother feel special  Carring and joyous home environment  Sensitive people can get depressed even when family supports them |
| Interviewer: Sahi. And do you think keh screening se koi fark parega? |  |
| Interviewee: Uhhh, screen se bilkul fark parega because urmm kyunkay aap agar kisi cheez keh lye pehle se tayar ho keh bethengay toh you will be treating it nicely, aap ussay theek se saray aspect treat kar sakengay agar aap pehle se tayar hongay iss cheez keh lye. Aur shuru keh aspects se agar aap urmm shuru keh point se dekhengay agar toh cheezen kharab hojayegy akhar main to phir cater karna shuru karengay toh wo obviously mushkil hoga | Screening leads to early identification and so it can help outline the best treatment procedure |
| Interviewer: Koi therapies ya treatments jo apko lagta hai kaam asakty hain for mothers? |  |
| Interviewee: Urmm |  |
| Interviewer: Jinkay baray main apnay suna ho for maternal depression? |  |
| Interviewee: Nhi mainay nhi suna | Never heard of treatments |
| Interviewer: Koi peer support ya support groups kaam asaktay hain? |  |
| Interviewee: Bilkul asaktay hain. Support groups hotay hain but I donot know if they exist in our society toh urmm I will not have any idea about this | Support groups can help  But support groups maybe donot exist in Pakistan |
| Interviewer: Any other comments that you'd like to give? |  |
| Interviewee: About? |  |
| Interviewer: Generally, anything urmm after discussing this entire interview. Anything you'd like to add. |  |
| Interviewee: Urmm I think in the start, I've talked much about it. Mainay sara introduction aur sab bataya jo bhi I, I think I've seen in my experience, urmm related to the medical practice of two years urmm patient kyunkay urmm different cheez se guzar raha hota hai, its basically urmm for the mothers urmm or going to be the mothers for the first time basically shayad unme depression ka element zada prevail karta hai, not the mothers who are second or third jo do teen bachay paida kar chuki hain maybe, toh unme itnaa depression ka element shayad na ho because history matters alot. The patient who is delivering for the first time is more prone to depression I think and for the screening as well. | New mothers are more prone to develop depression than mothers giving birth a second or third time |
| Interviewer: Alright |  |
| Interviewee: Theek hai, toh ye, ye aik element hai jo main mention karna chahungy. That's it. |  |
| Interviewer: Alright. Thankyou so much ma'am. Thankyouu. |  |

| Dr.Halima  *Briefing provided - verbal and written consent obtained* | |
| --- | --- |
| Interviewer: Can you please share your understanding of the health problems faced by mothers during maternal depression? |  |
| Interviewee:  It’s umm problems of coping I think which are very important, which they face and umm most of all I think its coping with the new arrival and the new responsibility which might create this problem and umm other problems would be that people don't understand what they are suffering from and they can't explain themselves because they are already suffering with a condition that they might not be very expressive at that time so its umm less chances to pick up and less chances to acknowledge this problem and moreover they already have a problem with coping so all these can be a reason for poor health of a mother while she is suffering with maternal depression. | Weak coping mechanisms  Added responsibility of child  Lack of understanding of their own suffering  Unable to express themselves  Less chances of diagnosis  No one acknowledges the problem  Adds to mother’s poor health |
| Interviewer: So ma’am ap ne kaha less chances to cope less chances to pick up why do they not have a lot of chances to cope or pick up? What is missing? |  |
| Interviewee:  They might not be having that will to cope up and umm moreover they might not be set mechanisms in the health care systems who are there already or the tools may be missing to diagnose such cases and these cases already happen in a place where there is a lot of work volume of healthy mothers otherwise prostate mothers so that’s why I think they might not cope and they might not be picked up earlier. | Willingness to cope is questionable  No set mechanisms in healthcare system  No tools to diagnose the issue  Increased work volume |
| Interviewer: so, have you ever witnessed a case that left an impact on you related to maternal depression? |  |
| Interviewee: We do come across a lot of cases umm and yes, I do have a case when the mother was not ready to take care of that child and the in-laws were constantly rather than reassuring her they were taking the child away saying that she has some vision problem or something like that and the child was not handed over to her. It even complicated and complexed the issue rather than solving it up.  So, that is what left an impact. | Mother was unable to look after child  In-laws tried to keep child away from her  Lack of support from in-laws complicated issue |
| Interviewer: so where you able to do something for the mother? |  |
| Interviewee: Yes, umm we had a consultation then first of all we interviewed her and we found out that she really is suffering from this problem and the she was referred for an expert help. The family was also counseled about this problem and the problem got solved later on. | Referral was used  Family counseling provided |
| Interviewer: All right. So what is exactly on the basis of all this - what is exactly your umm understanding of maternal depression? |  |
| Interviewee:  It’s a condition umm which has medical reason behind it and umm it happens in mothers who have recently given birth or ummm who have recently suffered an abortion umm so and a mother has an emotional condition which does have some physical impacts as well and one must be able to have a high indication - a high indictment - suspicion in such cases so that we can pick them early. | Medical condition  Recent birth givers suffer  Recent aborted women suffer  Events involving intense emotions have a higher chance of effecting mother negatively  Emotional condition should manifest physically for quick diagnosis |
| Interviewer: Right. So you said that you know it has a lot of emotions related to it? |  |
| Interviewee: Not only emotional but physical as well. |  |
| Interviewer: What kind of may be if you can describe some emotional and physical symptoms? |  |
| Interviewee: They keep quiet, they keep on crying they say that we are unable to take this new responsibility yet and umm sometimes they don’t talk to people they don’t take care of that child. Or they are constantly crying or screaming or the amount of pain which they are suffering is not that much umm but the behaviour might be very very different. They might suffer excessive crying behaviour or something like that. | Stay quiet  Crying spells  Less talkative  Do not look after child  High levels of pain |
| Interviewer: Right. To ma’am do you think k there is a lot of burden in the society because of maternal depression? |  |
| Interviewee: Actually, we might not pick up the case exactly so we exactly don’t know what is the burden but there is there is significant volume of such cases but I am still hopeful and I think in my personal opinion that our family set up and a lot of supportive mechanisms by in-laws and the family of the girl and boy as well has left an impact on me at least that it’s not a very big issue but when it happens then it needs to be picked up and we need to be sensitized about these cases. | Cases not diagnosed so cannot pinpoint level of burden  Significant volume of such cases  Appropriate support increases manageability  Sensitization is a must  Learning to diagnosis is important |
| Interviewer: Right. So what do you really understand by prenatal postnatal depression? |  |
| Interviewee: umm we don’t come across prenatal depression but a lot of anxieties in prenatal cases as far as I know in my specialty but we do come across a lot of postnatal depression cases it is a spectrum of diseases which starts from simple blues till depression to psychosis which a woman can suffer that is what I know from my knowledge. | Spectrum of diseases - Baby blues to depression to psychosis  Less cases of prenatal depression  High anxiety levels noticed during prenatal period  More cases of postnatal depression |
| Interviewer: umm so you know spoke about blues till the psychoses path so how long do you think does maternal depression over all last? |  |
| Interviewee: It might last for six to seven weeks usually umm and in cases who are very resistant all who end up in psychoses might end up for a longer period even a year or sometimes even they might reoccur in next pregnancies. | 6 to 7 weeks  Psychosis may last a year  Higher chance of recurrence in next pregnancy patients suffering from psychosis |
| Interviewer: So is this always going to be just after birth or also before birth? |  |
| Interviewee: It can. It might happen before birth. | Can be before birth |
| Interviewer: Right. So what would be the symptoms in maternal depression? |  |
| Interviewee: Usually I think it would be just keeping quiet or umm sleep disturbance, excessive crying or even screaming at times. Sometimes, complaining of excessive pain which is not there, the somatic issues related to psychological issues or unable to eat, unable to concentrate, unable to take care of the new born, unable to talk to the husband on this new arrival. These are the usual behaviors or excessive craving even at times of eating odd things which we never eat and wanting to eat a lot. | Staying quiet  Sleep disturbance  Excessive crying  Screaming  Somatoform disorder - Complaints of excessive pain  Change in eating habits  Unable to look after child  Low concentration  Unable to discuss the baby with the spouse |
| Interviewer: so umm which mothers would be most prone to develop these symptoms? |  |
| Interviewee: Someone who doesn’t have a lot of support I think and someone who has lost a baby, someone who suffered intrapartum complications like aph or pph or blood transfusion or hypertension or diabetes some medical condition which has given a bad impact on her emotional behaviour, a family history, a past history all these perspectives or a overall poor health poor living conditions or someone being very intelligent or very sensitive as a personality. | Little support  Suffered from painful and stressful physical condition adding to emotional turmoil  Family history  Past history  Poor living conditions  Poor Health  Sensitive mothers  Intelligent mothers |
| Interviewer: So you think it's not just one particular factor but a lot of different factors? |  |
| Interviewee: Lot of factors which might combine to bring this problem. | Amalgamation of different factors  No factor works in isolation |
| Interviewer:  So they'll always be associated or can it also be just one particular problem? |  |
| Interviewee: Yes like losing a child, the bad behaviour of the caregiver during childbirth or a complication during childbirth. | Losing a child  Mistreatment by caregivers  Complication during childbirth |
| Interviewer: So am I right in assuming that if it is a major trauma to the mother then she is affected more by just by one factor otherwise it will be more factors? |  |
| Interviewee: Yes. | It can be one factor if it’s a major trauma |
| Interviewer: So um in such cases how can you manage maternal depression? |  |
| Interviewee:  We need to diagnose them first number one we have to pick up the cases from the postnatal wards and we have to really categorize what category are they suffering from if it's just a blue or depression or psychosis and accordingly we have to see what are the causes behind it is it just cause which is resulting or its  just depression on its own which is happening or a hormonal imbalance or a disease which is causing it or a pain we have to relieve the medical condition we have to put her at comfort and then we have to see by involving the family giving her more rest, taking care of the child and  giving her reassurance that she is capable of taking care of this new responsibility and if it all doesn't help then after categorizing them we can refer them for an expert help from a psychologist umm or a psychiatrist. | First step is diagnosis  Categorize them based on where they fall on spectrum  Confirm the cause and effect relationship – hormonal imbalance, depression itself, disease, painful medical condition or psychosis  Counseling family  Letting her rest  Taking care of the child  Reassuring her and making her believe in herself  Lastly by providing psychological or psychiatric sessions |
| Interviewer: So what would let’s suppose if a mother is suffering from maternal depression what would be the short term and long term implications on the mother? |  |
| Interviewee:  Short term implications umm she tends to lose her confidence umm long term obviously she might lose her ties in the family later on, the family might say that you should have taken care of the child that's why the child is suffering with this and that so this can leave an emotional impact in short as well as short term impact. | Short-term: Loss of confidence  Blame game by family if child is neglected  Long-term: Loss of family ties  Negative emotional impact |
| Interviewer: Right so with this short term impact would it be just this impact or will there be something more or a physical impact because of it? |  |
| Interviewee:  There are cases where it has been reported that the mother has been very abusive to the child or she has been beating the child up or bad hurt to the baby affect all of this all this can have its impact long and short term. | Abuse towards child  Beating the child |
| Interviewer:  Basically the Infant is also affected? |  |
| Interviewee: Not only infant but the relationship with husband and with in-laws and with her own family | Infant is affected  Relationship with husband suffers  In-laws and personal family ties suffer |
| Interviewer:  How can the family be generally or the Infant be generally affected by the short term or long term impact? |  |
| Interviewee: It won't be umm everyone would be taking care of the mother with child might not have that attention and the person whose who has to get the attention to the child is herself suffering with the mental or emotional conditions so she might not take care of the child so umm the child might be suffering from poor growth or poor nutrition later on even death off such children and morbidity like diarrhea, pneumonia. | Distributed attention between child and mother  Child deprived of mother’s care  Poor growth and nutrition status of child  Death of child in extreme cases  Morbidity because of diarrhea and pneumonia |
| Interviewer: In the long-term how would it affect the child? |  |
| Interviewee:  If the mother has been treated I don't think it might have a long term impact but if the nourishment has been has suffered a lot then it might have a long term affect | Lack of nourishment for long results in long term affect |
| Interviewer: All right so based on all this do you think that the caregivers other than the associated partners will also be affected? |  |
| Interviewee:  The caregivers might have an impact like umm I remember that case or my other colleagues might be remembering such cases but we do think that what did we do wrong to this lady that this happened to her was it something wrong in my behaviour or my care which has resulted in this we could be judgemental to ourselves otherwise I don’t think it might be affect the caregiver a lot it does affect the family and the woman and the child and the caregiver to the extent that when till the time that woman is in the ward. After that we usually tend to forget as we have new cases coming up new work coming up. | Caregivers (doctor) might question their behavior towards mother  Judgmental attitude remains till woman is not sent home |
| Interviewer: So if I take the caregivers to be a family extended family or not just her husband but family living with her or her mother side of family then would they be affected? |  |
| Interviewee: Obviously, they would also be thinking that what did they do wrong to this lady. | Family questions its behavior |
| Interviewer: So you think it will be a lot of guilt? |  |
| Interviewee: yes. | High intensity of guilt felt by family |
| Interviewer: …and would it just be guilt or there will be emotional or physical symptoms other than that? |  |
| Interviewee: There might be emotional symptoms of aggression against that woman or umm depression in themselves at what did we do umm that’s it or guilt. | Emotional aggression (anger towards women)  Depression (Sadness) within themselves (family) |
| Interviewer: So umm do you think it’s managed in Pakistan maternal depression? |  |
| Interviewee:  In tertiary care centers and in teaching care hospitals especially in private hospitals I think, yes, but I still think that this problem is not identified to the extent that it needs to be identified. | Private hospitals, tertiary care hospitals and teaching hospitals manage it  Identification still remains an issue |
| Interviewer: So based on that do you think that these hospitals you just spoke about in their Gynae/Obs ward do you think that there are hospitals that will use tools to diagnose maternal depression? |  |
| Interviewee: umm when the patient reports then obviously the tools should be used. | Tools to be used when patient reports it |
| Interviewer: But is it ever part of initial screening that is conducted before the patient reports? |  |
| Interviewee: No, not at all, I haven’t witnessed in my practice we don’t put a tool to diagnose a postnatal depression in all the cases only when the symptoms arise or when the thing that the patient is not behaving well then we put up that tool otherwise we don’t use it to screen people. | No tool used to screen postnatal depression  Tool used only when symptoms are evident |
| Interviewer: So when you are doing your first history examination are there any questions related to whether she is going through trauma or if she is depressed in any way of form? |  |
| Interviewee: There is an antenatal care. We do ask questions pertaining to their domestic  living conditions like any violence or stuff or umm her nutritional condition we do ask questions pertaining to that and in her past medical history we do ask any past medical history of this this this or umm any problem in her postnatal period but we usually not tend to ask questions pertaining to psychiatric or emotional behaviour because it taken as a stigma in our society and even if we ask clearly the patient might not be very comfortable in answering such questions. | Antenatal care questions pertain to domestic violence, nutritional condition and past medical and pregnancy history  No questions asked about emotional behavior or psychiatric issues  Stigma in society  Patient may not answer comfortably |
| Interviewer: So did you ever get a chance to see a screening tool or have you ever used a screening tool? | Scales are used by some senior professionals |
| Interviewee: Yes, I do have used some umm what’s the name umm it’s about a place jis ke upar wo tool ko name kia tha. |  |
| Interviewer: HTP use karte hain aap log? |  |
| Interviewee: Nahin nahin Birmingham or something. |  |
| Interviewer: Edinburg scale hai? |  |
| Interviewee: kuch aesa hai kisi jaga ke naam pe hai jo hum ne use kia tha. |  |
| Interviewer: so is it like a dedicated scale for maternal depression or basic depression? |  |
| Interviewee: it’s for basic depression not for maternal depression. | A basic depression scale |
| Interviewer: Do you believe that it can be treated? |  |
| Interviewee: yes. | Treatment is possible |
| Interviewer: so umm what different forms of treatment have you ever come across? |  |
| Interviewee: Umm supported treatment, behavioral treatment and umm and medical treatment in the form of anxiolytics, giving her rest, taking care of her nourishment. | Supportive & behavioral treatments and anti-anxiety medicines can help  Rest and good nourishment can be helpful |
| Interviewer: Can you explain a little about supportive and behavioral treatment? |  |
| Interviewee: Supportive and behavioral like we can involve the family we can ask the partner and the in-laws and the mother of the girl so that she can take care of the new born baby and she is given time to sleep and umm they are given under supervision the baby and mother are seen that how does she cope up with that baby how she is taking care of that baby and is she able to take care of herself and the child properly otherwise we can take the baby from her for few days if it’s helpful umm giving her time to sleep and giving her medication if needed would be needed I think. | Other family members can look after baby  Mother gets maximum rest  Baby is giving to mother under supervision  If mother is unable to cope with baby, she can be giving rest and medicines |
| Interviewer:  Right so umm based on the fact you know you just told you know these treatments do you think that mothers or families seek help? |  |
| Interviewee:  The mother herself? |  |
| Interviewer: Yeah and the family for the mother will they ever seek help? |  |
| Interviewee:  They do when the problem is really severe they do. Usually if it’s a mild case it might just go unnoticed but if it’s a severe case then they do seek help I think that is what we have seen even in umm underprivileged families they do come for help to health care providers. | Help is seeked only in severe cases  Mild cases go unnoticed  Such is the case with underprivileged houses too |
| Interviewer: So how would you define a mild and a severe case? |  |
| Interviewee:  By putting them on the scale for assessing depression and looking if only if supportive treatment helps it could be helpful in mild case and she is herself ready to take care of herself. But if she needs a medical treatment or an admission for that thing then it might be a moderate or a severe case. | Scale can differentiate between mild and severe depression  Mild is when mother is able to take care of herself  If admission is needed then it is severe or moderate |
| Interviewer: So how do you suppose you let family define that she needs treatment now? What would make her family come to a hospital for such a treatment? |  |
| Interviewee:  I think once they have started their own umm modified behaviours like giving the support, taking care of her, talking to her but she is not responding then they do come for the patients. | If mother doesn’t respond to modification of behavior  Modification can be providing support, talking to mother, taking care of her |
| Interviewer: So umm what approaches or what combination of approaches do you think can be used to best engage these mothers who are depressed? |  |
| Interviewee:  Once they are diagnosed with depression? |  |
| Interviewer: Yeah. |  |
| Interviewee:  Giving them the realization of their condition and telling them that yes, you are suffering with a medical condition. It’s not a psychiatrist illness but it is a medical recognized condition you are suffering from that might give them some supportive help and say that okay, yes this is a recognized condition which I am suffering and a lot of people do have this. We can tell them that this percentage of cases suffer with this so they might be reassured by this and moreover we can say that it’s not a long lasting condition you will be helped by taking this medication or rest or support you can be helped. So all this can be helpful for her. | Creating self-realization about condition  Validation that it is a medical condition  Belief that many people suffer from it  Quoting stats of affected mothers  Confirming that its temporary  Ensuring she understands importance of rest and medicines |
| Interviewer: So umm do you think that those mothers who are depressed they don’t know that they are depressed? Can we engage them in some form? How can we make them more aware? |  |
| Interviewee: I don’t have an idea. There might be given some tools to access their condition that how are you feeling are you sleeping well or something like that. | Not sure of forms of engaging mothers |
| Interviewer:  What are your perceptions regarding barriers for seeking health care for such a crucial issue? |  |
| Interviewee:  I think people might think that they will be labeled as mental people or psychologically ill people or weak people all this might be a hindrance or make people hesitant to seek help in such cases. | People are hesitant to seek help  Seeking help is a hindrance  People fear being labeled as mental, psychologically ill or weak |
| Interviewer: any other thing? |  |
| Interviewee:  and lack of awareness I guess. | Lack of awareness also stops people |
| Interviewer: Anything else that you can tell? Umm do you think that society does not place enough importance on it? And that is a problem? |  |
| Interviewee:  That could be the issue. | Maybe society doesn’t place enough importance on it |
| Interviewer: Umm what about cultural or religious beliefs? |  |
| Interviewee:  Religious beliefs I don’t think have any hindrance. Cultural, obviously I said that society might think that people might think that she is suffering with a mental illness might put a stigma on her. | Religious beliefs have no effect on it  Culturally, stigma affects negatively |
| Interviewer: Umm and do you think that socio-economic issues and education or language would have an impact? |  |
| Interviewee:  Language also doesn’t have an impact I guess but social issue obviously, when someone has to put up with living with it then they might not be umm very comfortable in taking care of the condition which is not making them suffer a lot which they think. | Language has no effect  It is a social issue  Economically, people might not invest since they believe it is not a major suffering |
| Interviewer: Umm would education or the fact that we have… |  |
| Interviewee:  ..Obviously awareness does but education level doesn’t  make a person aware but if someone is aware and educated and aware as well then they might seek help earlier and. | Education does not add to awareness levels  Special awareness is important  Awareness coupled with education can lead to quick health seeking behavior |
| Interviewer: Um and what about the fact that do you think that we have enough human resources to look after this condition if it is explicitly mentioned by all mothers? |  |
| Interviewee:  We do have psychiatry specialists and psychologists but the counselors and caregivers in our maternity units are usually lacking because they are usually busy units. | Maternity wards are busy units  Psychiatrists or psychologists are lacking |
| Interviewer: Right so do you think that a maternity unit should have a unit for counseling? |  |
| Interviewee:  Yes. Not only for this but for a lot of other things as well. | Maternity ward should have a counselor  Other issues should be looked at by a counselor too |
| Interviewer: Right. So what other things? |  |
| Interviewee:  About their contraception, about their best feeling all these things suffer because of not having a counselor all the times. | Feelings regarding breast feeding, contraception should also be discussed with counselor |
| Interviewer: Right umm so how would a patient perceive maternal depression? |  |
| Interviewee:  I don’t have an idea but I think she perceives that she is suffering or that she might feel sad or she might feel guilty anything could come to her mind. | Perceives herself to be a sufferer  Can think of different things  Can be feel sad or guilty |
| Interviewer: Right. So do you think that our educational framework Pakistan’s educational framework has a role to play in the management of maternal depression? |  |
| Interviewee:  If it’s a medical school obviously otherwise for a general school. | Medical framework plays a part |
| Interviewer: No, a particular medical school. |  |
| Interviewee:  A medical school should put some importance and it is usually taught in medical schools. |  |
| Interviewer: So, it’s part of our curriculum? |  |
| Interviewee:  Yes, I think not only part of medical curriculum but for post-graduate training. It has some marks I guess. | Counts towards all marks in post-graduate training |
| Interviewer: And does it have a lot of importance associated to it? |  |
| Interviewee:  They do put questions that’s why I think that it is considered as important by the at least by the examination body and by the training body. | There are questions in the final exam  Examination and training body does lay some emphasis |
| Interviewer: Are there ever any on job trainings, workshops? |  |
| Interviewee:  We have come across workshops I don’t know about the other institutes. | Has come across workshops |
| Interviewer:  So in your experience while you are heading the department in a way do you ever come across trainings that are there for the students or the way they are completing the… |  |
| Interviewee:  There was umm a workshop and lecture conducted and a public awareness seminar conducted by our psychiatry department for all the health care providers and at that time they did put maternal depression as well but I don’t know about a general trainee whether they went to attend that or not. | Workshops and lectures for students are present  Psychiatry ward of institute conducts such workshops  Maternal depression is part of workshop |
| Interviewer: So it was not a mandatory training? |  |
| Interviewee:  No, it wasn’t a mandatory training. | Workshops are not mandatory |
| Interviewer: …and any CMEs that you might know about that take place for this particular issue? |  |
| Interviewee:  No. | No CMEs |
| Interviewer: Do you think that - earlier you mentioned you know that the caretaker the caregiver might feel that you know it’s their responsibility or they did something wrong so do you think that self-efficacy is related to the rate of diagnosis and the management of maternal depression? |  |
| Interviewee:  Obviously, if someone is well aware with what is this condition and how does the patient behave what are the symptoms and signs of this condition then it the sign the rate of picking it up would increase. | Self-efficacy plays an important role  Rate of diagnosis is proportional to HCPs knowledge |
| Interviewer: So how responsible is a person working in genecology ward in terms of diagnosing maternal depression? |  |
| Interviewee:  That is the prime responsibility he would be the - or she would be the first person to diagnose and then they would be able to transform. | Maternity ward HCP is primarily responsible  First point of contact  Can transform mother’s life |
| Interviewer: So do you think that they can feel compassionate and they can show their empathy to the patient considering there are always so many patients around? |  |
| Interviewee:  Usually they are very empathetic because women as such are not very privileged part of our society especially the groups who are coming from a poor background or a low socio-economic groups and the doctors and the nurses and especially the junior doctors are very very empathetic usually to take care of the stuff and the blood arrangements and for medications and they usually may be very empathetic if they find out that the patients is suffering from psychoses or depression. | Women are not privileged lot in our society  Poverty makes matters worse  HCPs are empathetic  Junior doctors are more empathetic than nurses and senior doctors  Usually more empathetic if patient is diagnosed with MD |
| Interviewer: So you mentioned the junior doctors do you think that as we go up the levels the level of empathy sort of reduces? |  |
| Interviewee:  it’s not the empathy which reduces I guess it’s a lot of other work. | Empathy does not reduce over time but work load increases |
| Interviewer: Right |  |
| Interviewee:  The administrative work on a senior doctor increases she has to take care of the whole ward rather than junior doctor would be taking care of those ten beds that she is responsible for so that would be one reason otherwise empathy never reduces. | Administrative work counts for senior doctors  Junior doctors are assigned just 10 beds  Senior doctor heads whole ward  Empathy never reduces |
| Interviewer: …and what about being sensitized do you think it’s just time or do you think people also get sensitized… |  |
| Interviewee:  Sensitization can occur all through your life and with any kind of session or any CME or any such activity can sensitize people | Sensitization is not a one-off process  Can occur all through life  Session, CME, activities can sensitixe |
| Interviewer: Do you think that infrequent follow ups which lead to weak doctor-patient relationship have an impact on a mother and it might reduce the chances of diagnosis or it increase the chances of diagnosis? |  |
| Interviewee:  Yes, that could be one reason. | Infrequent follow-ups can be one reason |
| Interviewer: Do you think that general practitioner should be first line of support for a person to be diagnosed? |  |
| Interviewee: It could the general practitioner system in our society is still not the way it has to be. People do see their general practitioners but even there they don’t come up with a lot of personal problems but still as compared to a tertiary care hospital they might be comfortable in discussing this with their general practitioner so they definitely need to be trained and sensitized about this issue. | GP system is flawed in society  People don’t discuss personal issues with GP  People are more comfortable with personal GP  In comparison, people discuss more with GP than TCH doctor  GPs need to be sensitized and trained |
| Interviewer: So what can a Gynecologist do from her end? |  |
| Interviewee: Gynecologist usually come across in their public practice obviously we come across such patients not very often but in private practices yes, they do come across such patients very often and their follow ups and they should put up a few questions in antenatal and postpartum mothers especially pertaining and directly asking about the symptoms of postnatal depression and so it might increase the rates of picking up these cases. | Less chances of coming across MD patients in public practice  Private practitioners should ask specific MD questions during prenatal and postpartum period  Rate of FU is high in private patients |
| Interviewer: ….umm right so in a hospital setting what factors do you think one you mentioned right that would increase the chances of diagnosis so in a hospital set up how they may play a role in the diagnosis and management of maternal depression? |  |
| Interviewee: Would you like to make your question clearer? |  |
| Interviewer: I am trying to understand that within a hospital setting what would be the reasons for which it might not be diagnosed and managed or it might be diagnosed and managed? |  |
| Interviewee:  It’s the number of cases, the number of staff it could be not appropriate for the care of lot of patients if there is the number of the staff taking care is lesser then they would obviously be taking care of the vital signs and their medications and their cleaning and vaccination of the baby and breast feeding and other signs rather than coming to the emotional thing. | High number of cases  Shortage of staff  More important is vital check, medicines, cleaning, vaccination, breastfeeding. |
| Interviewer: Do you think that physical aspect is a lot more important at that given rather than the emotional aspect? |  |
| Interviewee:  That is what I think. | Emotional aspect is not as important as physical symptoms |
| Interviewer: That is what you think…. Hmm…Okay |  |
| Interviewee:  That’s why it could be a very important factor because we have so many other things in her physical examination to look for that we don’t go for the emotional examination. | Physical examination is long  No time for emotional examination |
| Interviewer: So do you think that we have pro-active tools even if we want to if a gynecologist wants to do it? Do you think we have pro-active tools to monitor the mother? |  |
| Interviewee:  We don’t have them at practice. | No pro-active monitoring tools are present |
| Interviewer: So how do referrals generally work? |  |
| Interviewee:  Referrals if they are done properly and responsibly they do work a lot and if you follow up with those specialists then they do work otherwise if the referral has not been very responsible it has been very casual then the behavior from the person that you have referred might not be responsible as well. | Proper, responsible referrals work  FU with specialist is the key  Mother may act irresponsibly if referred casually |
| Interviewer: So let’s say if you have a referral and the patient does have depression, what will happen next? |  |
| Interviewee: The specialist will treat that patient. | Specialist treats patient after referral |
| Interviewer: What specialist should be responsible? |  |
| Interviewee:  Psychiatrist. | Psychiatrist is responsible for treatment |
| Interviewer: So she would be directly referred to a psychiatrist? |  |
| Interviewee:  Or a psychologist. | Can be a psychologist |
| Interviewer: …and would you follow up again with a psychiatrist or a psychologist? |  |
| Interviewee:  Obviously we will write it on referral that send this patient back or when the patient will come for that medical follow up we will ask what did the doctor say what medicines did they put you on. | Patient slip has notice of referral  On FU visit, Gynecologist inquires about meeting with counselor |
| Interviewer: If in case the patient doesn’t come back… |  |
| Interviewee:  Then we might not follow if we don’t have the contact number of that patient. | No FU if patient does not return  No contact directory of suffering patients |
| Interviewer: So coming to the last question umm what are the factors that can help protect a mother from maternal depression? |  |
| Interviewee: This is making them aware and making the healthcare providers aware of that condition and picking them early and treating them early. | Awareness for mother  HCPs awareness |
| Interviewer: So do you think that early identification helps? |  |
| Interviewee:  Yes, yes. | Early identification can help |
| Interviewer: Any therapies that you can name that might help a mother? |  |
| Interviewee:   They might practice exercises regular physical exercises might be helpful. | Physical exercise helps |
| Interviewer: Right and do you think that groups or peer support something like that would help mother? Do you think that it should be a norm? |  |
| Interviewee:  That can be helpful but umm it depends upon would they go for such group support therapy but it’s not a practice basically if we do practice and then the patients don’t utilize them we might blame them but as we don’t have a lot of support groups practices in our pregnant population that could be one thing to put in the practice. | No support groups  Peer Groups is not a common practice  Patient cannot be blamed  Should be added to practice |
| Interviewer: All right. So any other comments? |  |
| Interviewee:  Umm I think that’s it. |  |
| Interviewer: Thank you so much ma’am. |  |

| Dr Sana  *Briefing provided - verbal and written consent obtained* | |
| --- | --- |
| Interviewer: Can you please share your understanding of health problems that are usually faced by mothers during maternal depression? |  |
| Interviewee: Yaar, there are patients coming to us like suffering from this not in the antenatal period but maybe known cases of depression and psychosis or any other diseases related to like this field. But are you looking for the reasons why? | Early identification is difficult since patients only visit when case is severe |
| Interviewer: Just generally what is your understanding? Is there is not there happening? |  |
| Interviewee: It is there. It is there, because since I'm working in a government hospital, usually patients coming to me are from a very low social economic group and women usually come to us with not for the antenatal checkup but so some complains like suffering from domestic abuse and some, they are like they have some fights and they're suffering from bad outcomes. We actually when inquire them and ask them keh what's their reason so there are patients which turn out they are suffering from depression as well and so for them we usually refer them to a psychiatrist. | It exists  Women don’t visit when pregnant  LES women often visit because of domestic violence  Depression is diagnosed through inquiry of social background |
| Interviewer: A case that left an impact on you? |  |
| Interviewee: Not much, but not really to maternal depression like not exactly that, but more of a domestic violence if you're asking for, but not of maternal depression exactly | No case of maternal depression has been impactful enough |
| Interviewer: Theek hai. So what's your understanding about maternal depression? |  |
| Interviewee: My understanding is basically is that a woman if she's suffering in her since the time of conception till the time of the partum she's suffering from, symptoms like she's not taking interest in herself aswell as in her baby and also  not paying attention towards the, the family and she's not taking proper medication or she's like her appetite and all with weakness and fatigue and tiredness and lack of sleep, so she is a patient of, suffering from maternal depression. | Women can suffer from conception to postpartum  Lack of self interest  Lack of motivation to look after family  Changed eating and sleeping habits  Weakness, fatigue, tiredness |
| Interviewer: So do you, you do believe it exists then? |  |
| Interviewee: Yes. |  |
| Interviewer: Alright, and what kind of a burden does it have on the society? |  |
| Interviewee: Well, if a woman is suffering specially in a pregnancy, obviously she will not take care of the baby and also this will affect the family, and if there are more patients like this…. The society will not be as it should be | Adds burden to society  Child rearing falls on shoulders of family  If too many patients suffer, society is affected negatively |
| Interviewer: So, what do you understand by prenatal and postnatal depression? |  |
| Interviewee: Prenatal depression is that a woman is suffering before conception. It's prenatal. And postnatal is the, when patient is suffering after the delivery of the baby. She doesnot have any symptoms in the pregnancy, like in the nine month time but after delivery, its postnatal, till partum | Prenatal refers to before conception  Postnatal is after delivery |
| Interviewer: Okay, and what would be the symptoms of this? |  |
| Interviewee: The symptoms of depression would be the same. Whether it is a normal adult man or woman, the symptoms would be the same. But in this, the women would have the tendency to kill the child as well… | MD and Depression effect the mother similarly  MD mothers can kill child as well |
| Interviewer: hmmmm |  |
| Interviewee: So that is the only difference aside from tiredness fatigue and lack of interest in daily activities around her and weakness, and that's it. |  |
| Interviewer: And what would be the time duration for maternal depression? |  |
| (Short pause) |  |
| Interviewee: It could start anywhere, anytime, but mostly it's post-partum if it’s started after 24, within 24 hours and that could turn into postnatal depression if the symptoms persist for two weeks | MD can occur any time  Postpartum is more common  Postpartum occurs within or after 24 hours |
| Interviewer: Alright, okay. So who's most at risk for developing maternal depression amongst mothers? |  |
| Interviewee: A woman who has a known history, family history who's already effected or her mother has the same symptoms in her peegnancy or if she has a bad like obstructive history like miscarriages or bad obstructive outcomes like repeated intrauterine issues or fetal demises so such patients are already very sensitive and the patient suffering belongs to a very low social economic group doesnt have any proper financial supports, doesnt have a supportive husband or family, there's no one to look after them so such women are very likely.. prone to develop this | Known personal history  Family history  Bad child birth history – miscarriages, obstructions, fetal demises, intrauterine issues  Sensitive mothers  LES mothers with no financial support  Lack of supportive husband and family  Women not well cared for |
| Interviewer: Alright, so how can it be managed? Maternal depression |  |
| Interviewee: It can be managed in a multi-disciplinary clinic apart from OBGYN, a proper psychiatrist or well-trained nurse should be there and there should be proper screening tools to assess the severity and manage it accordingly | Multi-disciplinary clinic needed to manage it  Nurse or psychiatrist present at all times  Renowned screenings tools usage  Assessment of severity  Knowledge and awareness of appropriate management techniques |
| Interviewer: Alright, so what are the short term and long term implications of maternal depression? |  |
| Interviewee: The short term implications will be that the women will be suffering and that she will not take care of the kid, the baby and the long term that she can go into psychosis or she can go into such a state of depression that she cannot, like be cured well enough. She might need high doses of anti-depressants throughout her life and obviously if she cannot take care of her kid properly, that kid would not have a proper upbringing and that phase of the life which baby needs the mother at most that would be like deficient in her life, in his life and which could affect his entire life as well, the life of the individual depends at proper period of infantile period till 5 years of age. If the baby doesn't have the proper parents, she'll be like…what do you expect them to be like! | Mother will suffer  Lack of care for child  Psychosis  A state that cannot be cured easily , leaving a long term effect  Requires high doses of anti-depressants for mother to get well  Baby loses out on infantile period of life |
| Interviewer: Theek hai. And do you think family members are affected? |  |
| Interviewee: If the woman is effected in the family, its effects on family… yes it does because if you have a patient suffering from depression at home, and she is not in her proper state, she can not take care of herself, all the time she is, she is weeping, she's not eating properly, she's not sleeping, so the family would be effected aswell because they feel so helpless, she cannot help her. Just taking to a doctors and hospitals, and it does effect them economically aswell and mentally aswell, so it does | Mother is unable to look after herself  Weepy  Family feels helpless  Increases financial liability  Lack of mental peace at home |
| Interviewer: And what about other caregivers and associated partners? How would it directly or indirectly affect her husband? |  |
| Interviewee: Yes, it does because the women would not, when she's not taking interest in herself how will she take care in interests of her husband's life or she will not be paying proper attention to him aswell. | Husbands needs are not met  Husband is deprived of wife’s attention |
| Interviewer: Alright, and in your opinion, how is maternal depression managed in Pakistan? |  |
| Interviewee: See, I don’t have the exact thing, because I haven’t treated patients myself. What we do is that we refer them to psychiatrist because it's the better field. Because we give the antenatal care and the post natal care, but for this matter, it’s better that she should be managed by a trained individual and which is a psychiatrist. So it’s better they do it, usually by counseling, medication, and follow ups, and all and that's it. | Psychiatric referrals are used  Never been in a situation to diagnose or manage MD  Gynecologists are only expected to provide postnatal and prenatal physical care  Specialists are held responsible for psychiatric care  Counseling, medication and follow-ups can help |
| Interviewer: So do you think hospitals use tools to diagnose maternal depression? |  |
| Interviewee: Yea, there are certain tools but not practiced in our department, because we're not dealing with it exactly, but yes, there could be Edinburgh Scale I guess, so yea they are practicing but in tertiary care but also in a private setup, like AKU, Liaquat National. But if you're expecting this to be in a government setup, then I'll say no. | Tools are present  Tools are not used in Gynae ward  Not Gynecologist’s job to deal with MD  Tools are used by specialists in private hospitals not government ones |
| Interviewer: Alright, so do you think it is a part of the initial screening, conducted with a mother when she reaches the hospital? |  |
| Interviewee: Yes it should be. | Screening should be part of initial screening form |
| Interviewer: It should be, but it's not at the moment? |  |
| Interviewee: Yes |  |
| Interviewer: Alright, and in your experience have you ever used or seen a Scale? | No scales used by Gynecologists in hospitals |
| Interviewee: No |  |
| Interviewer: Do you think that maternal depression can be treated or managed? |  |
| Interviewee: Yes | MD is treatable and manageable |
| Interviewer: How? |  |
| Interviewee: Again, first stage is the counseling and then medications and there are not certain investigations. In this case, in depression, you dont need to have the CT scans or any investigations, its just you diagnose the patient by just taking the history and looking at the patients and again the prescribing the medications | Counseling is first stage  Medications  Scales are needed since it diagnosed based on history and patient’s psychological evaluation, not physical examinations |
| Interviewer: Okay. So if effective treatments do exist, do you think mothers and families are seeking help? |  |
| Interviewee: Not on a large scale, because they find it a bit difficult to admit they're suffering from this disease. They usually report it again at a very late stage not in the initial phases because if they report it initially, its better and the results would be much much better | Lack of acceptance by sufferers about their condition  Early identification is difficult since not reported in initial phases |
| Interviewer: Okay. So what combination of approaches or what specific approach do you think can best engage mothers that are depressed? |  |
| Interviewee: (short pause) |  |
| Interviewee: Social support groups. There should be certain workshops organized on mental health awareness and there should be certain activities for engaging them so that they can get out of it and see the positive aspects of life and get out of their negativity. | Social Support Groups  Awareness workshops on forms of engagement  Promoting positivity via awareness sessions  Mental health awareness sessions |
| Interviewer: So, based on all this what are your perceptions regarding barriers? Why can they not seek this help? |  |
| Interviewee: Probably their family, their in-laws. Because to them, like mental, mental depression or any mental illness in her family, like they're least bothered to deal with it. If like unless they're suffering from a cardiac emergency, they will definitely take her to the hospital. Like they see its life threatening, but mental illness, it's very slow. It's very slow in progress and they see it like initially. They see that she's maybe she's behaving like this to avoid the daily, daily household activities and she's just making excuses and she's just portraying it and that's it. They don’t see it she's actually suffering from a very, devil disease which can affect her life and the baby's as well and the family | Family and in-laws neglect the issue at hand  Lack of importance since it has no immediate physical symptoms  Mental illness is slow  It is not as life threatening as some physical conditions  Believed to be mothers way of avoiding her responsibilities  It’s a devilish disease – slow and unseen  Affects the baby negatively  Affects the family negatively |
| Interviewer: So do you think except for importance of social acceptance, stigma plays a role in this? |  |
| Interviewee: Yea sort of | Stigma plays a role |
| Interviewer: How? |  |
| Interviewee: Actually I don’t think so that it does |  |
| Interviewer: It doesn’t play a role. So why would you say it doesn't play a role? |  |
| Interviewee: No, I don’t think so |  |
| Interviewer: So you, you say that stigma does not play a role. So how do you think it does not play a role or why would you say that it does not play a role? | Unable to comprehend the term social stigma |
| Interviewee: Not exactly anything, it's just that I don’t think so |  |
| Interviewer: So do you think cultural aspects of society play a role in it? |  |
| Interviewee: The cultural aspects in a woman suffering from mental depression? Very least likely | Cultural aspects play no role |
| Interviewer: Okay |  |
| Interviewee: Because, like the culture we're facing, it could like if a woman who is working, who is employed and who is facing the hardships and she is like a single mother and all toh probably the social load on her or some trauma she's suffering from that, some circumstances which she had faced in the past could lead to it. That's it, but apart from that, not much. | Working mothers may be culturally affected due to work-load  Past history  Emotional trauma |
| Interviewer: Okay, what about religious beliefs? |  |
| Interviewee: Religious beliefs. There are certain diseases, like for instance, epilepsy. When a patient come to us with epilepsy, her family member, jesay like she's suffering from like you know, jinn agaya hai uskay upar like iss tarhan keh jo beliefs hotay hain, so there are people because they don’t have any awareness. So they think something has possessed her or, especially in the villages, they see it. Because people when go to the graveyards, it’s a myth that they might encounter something or something happens to it, toh yes religious beliefs | Religious beliefs play a role  People believe it to be some supernatural power’s affect |
| Interviewer: Okay, and what do you think about supply side determinants? Do you think hamaray paas itnay resources hain keh agar hum provide karna chahen toh kar sakengay? |  |
| Interviewee: Yes, why not. If you're asking me keh we can incorporate this component in our antenatal care, and we can also have this in the clinics, we can. It will hardly take, if there is a proper scale, like I think there's even book ki bhi scale whatever's the rating in which asking four to five questions hardly from ten questions. You can assess a patient, she's suffering from depression toh that would be far far far much better | Component can easily be incorporated in antenatal care and clinics  Easy and less time consuming scales can help develop quick insights  A better way is to ask 4 to 5 questions than asking nothing |
| Interviewer: Theek hai, and how do you think that a patient will perceive maternal depression? How does a mother perceive maternal depression? |  |
| Interviewee: She might find it like she cant be cured and like she will suffer it for eternity and she will feel very helpless, and very lonely and like nobody's there to look after her, so that's it. | Mother might believe it is incurable for life  Helplessness and loneliness |
| Interviewer: And what role does the medical framework has to play in this entire diagnosis, management and the treatment if maternal depression? |  |
| Interviewee: See the medical education thing is, if there are proper antenatal classes  and patients attend those classes, they can get aware because of this thing, that yes there is depression. The thing is depression, that women, they are not well educated they doesnot know about this they're actually suffering from. So if they have the proper awareness they could address it to the doctor initially like this this symptom is significant. If they are continuously feeling low, they have low moods or they are not feeling well or having mood swings and they feel tired, they can report it address it to the doctors and they might find it significant so they can treat her accordingly in the initial state. | No antenatal awareness classes incorporating MD for patients  Lack of education leads to lack of understanding  Mother can discuss symptoms with doctor if she has pre-existing understanding of situation  Continuous feelings of being low  Mood swings  Timely treatment can be provided if mother is able to pick symptoms herself |
| Interviewer: Alright, so do you think that doctors know how to diagnose maternal depression? |  |
| Interviewee: Yes |  |
| Interviewer: Specifically…. |  |
| Interviewee: But they dont want to. They know but they're not actually practicing it. Because in routine antenatal care we really look for past risk factors that affected her obstructive outcome, any gynecological issues, any medical issues, any past significant surgical history but we actually omit this part- the mental health thing. | Gynecologists have the knowledge required for diagnosis  Gynecologists are more focused on gathering physical history related to pregnancy  Mental health part is omitted |
| Interviewer: Alright. So you said the medical issues and the rest of the factors you mentioned are all physical in nature. So do you think when a gynae says medical issues, it is just physical then? |  |
| Interviewee: Medical issues are never physical. Medical issues by means if she said medical issues suffering from hypertension or asthma or thyroid disorders or thalassemia, because these are those disorders which are very common in our country | Gynecologists need to be careful of common medical issues such as asthma, hypertension and other such disorders |
|  |  |
| Interviewer: So do you think is there anything in your medical or nursing curriculum when people are joining the gynae department that they are taught about maternal depression? |  |
| Interviewee: No. | No training on MD when joining Gynae ward |
| Interviewer: And there's no on-job training or there is on-job training? |  |
| Interviewee: No, there's no on-job training. |  |
| Interviewer: Any workshops and trainings? |  |
| Interviewee: No. | No workshops or training |
| Interviewer: Any practical experiences? |  |
| Interviewee: I just attended a workshop that had seminar on depression, but that's it. Not more than that. | Workshops target depression sometimes but not MD |
| Interviewer: Okay, so you went there because you wanted to, or you were asked to do it? |  |
| Interviewee: I wanted to, that's why. | Workshops are not mandatory |
| Interviewer: Alright, so it was not a mandatory thing? |  |
| Interviewee: No it was not mandatory. |  |
| Interviewer: Alright, are there any CMEs that you might've heard of? |  |
| Interviewee: For…no, not yet. | Never heard of CMEs for MD |
| Interviewer: Not yet, okay. Do you think self-efficacy plays a role in diagnosing or treating or managing maternal depression? |  |
| Interviewee: What you do mean exactly by self-efficacy? |  |
| Interviewer: Do you think that health care provider herself needs to realize the importance, needs to know if she can do it, needs to know it’s her responsibility? |  |
| Interviewee: To screen the patient? Yes, she should. | HCP is responsible for screening patient for all ailments |
| Interviewer: Okay, so is it their personal responsibility? |  |
| Interviewee: No, they're not actually doing it. We are not actually doing it. | HCPs are not expected to screen the patient for MD |
| Interviewer: So, you think they should be? |  |
| Interviewee: They should be doing it. |  |
| Interviewer: Alright, and do you think there's lack of compassion and empathy? |  |
| Interviewee: Not exactly lack of compassion or empathy, but the thing is, the work load we're facing like we have four hundred to five hundred patients. In that, you, you seek the important things in the patient and we usually skip this part. Like, domestic violence is an important part, we should screen for that aswell but we dont have proper time for that. And for this reason, this thing, there should be a proper antenatal thing which should include these components aswell. I'll suggest this. | Excessive workload  400-500 patients daily  Importance is provided to physical issues over emotional issues  Domestic violence should always be screened for as safety measure but time doesn’t allow for it  Antenatal screening should include mental wellbeing questions |
| Interviewer: And what about comfort and confidence? Like, you feel very comfortable and confident talking to a mother about maternal depression? |  |
| Interviewee: Yes, ofcourse | Talking to mothers is not an issue for Gynecologist |
| Interviewer: So that will never be an issue? Talking to a mother? |  |
| Interviewee: No |  |
| Interviewer: Alright, so do you think there's a problem with, let's suppose you referred to a patient, do you think there'll be a follow up from your end or the patient's end? |  |
| Interviewee: Yup, follow up is a problem, because there are patients that we really lost to follow up and the phone numbers that are provided by them, they usually dont respond to them, the numbers belong to the husband, they like dont connect to their wives and all that. But, if in a private hospital, there is a proper system follow up as well. They, because the same CR number, on which the patient's registered, its circulated in different departments, like if she has a visit, she had a visit with the psychiatrist, or if she had a visit to the medically hour and then she's coming to the OBGY and the clinic, the entire record will be on that thing. So it's all electronic, you can also see what the past visits she had and, cause if she comes to you and names that or mentions to you that she had a visit to the doctor or the psychiatrist, then you can look into it. So there's a proper follow up, so you can again ask for the patient to again mention you or again come back. But in this, in government setup, the patients are usually lost to follow ups. | Follow-up is a problem  Phone calls are not received even if doctors want to follow-up  Contact numbers are not personal numbers and belong to male family members  CR numbers are used in private hospitals to follow-up  Patient data is centralized and can be accessed by all physicians of the patient in private setups only  Patients are lost to follow-up in government hospitals |
| Interviewer: So if, let’s suppose, there is a follow up, will the gynecologist of the patient be able to help? |  |
| Interviewee: The gynecologist will be able to help on a certain extent. Because this thing should be managed in a proper psychiatry department or multi-disciplinary clinics, like there should be combined clinics for that. One day in a week, once a week, like, the psychiatrist can come to our clinic and can have two hours for such patients and discuss with patients and we can have our antenatal care component, and he or she can have the mental assessment thing. | Gynecologist can only help to a certain level  Multi-disciplinary clinics are needed  Only psychiatrist can overlook MD  Antenatal care sessions can have sessions of psychiatrists incorporated within them |
| Interviewer: Alright, so do you think that the general practitioners should be the first line of support? |  |
| Interviewee: Yes of course | GP should be first point of care |
| Interviewer: Since the mother will generally always visit the gynecologist first. |  |
| Interviewee: Yes, because the people or the women come to our clinics or for registration not at early weeks. Like she usually came to us in the third or she usually come to us when she's about to deliver. We don’t know whether she's suffering from depression, before or during the pregnancy. So it's better they, the general practitioners should be the first line of investigators or the | Women visit Gynecologist in third trimester or right before delivery  It is difficult to assess mother’s vulnerability to MD in earlier months  Mothers visit GP even before Gynecologist |
| Interviewer: So what do you understand by the term general practitioner? |  |
| Interviewee: The local GPS that we usually have in the neighborhood | Local Neighborhood GPs should be well-versed |
| Interviewer: Alright, so what factors play a role in the diagnosis and management of maternal depression in a hospital setup? |  |
| Interviewee: Proper history, examinations. By this you can diagnose the case, and after you, once you've diagnosed it, then you start with the severity- whether she needs medication, whether she just needs counseling and whether the patient can be managed by just innovative management or she needs hospital admission, you need to admit the patient, isolate it from the family or further accordingly. | History and examination  Diagnosis  Assessing the severity and taking appropriate treatment measures – counseling, innovative management, hospital admission |
| Interviewer: Alright, do you think that overcrowding of patients plays a role in them not being diagnosed or managed properly? |  |
| Interviewee: Overcrowding, no I don’t think so, but yes overcrowding could lead to late diagnosis because the patients cannot get like, they reach us very late. I won't be lying, like there's a huge line patients has to like stand in those lines and have to OPD clinics and just then they're closed and patients have to go to the ER where a junior doctor assess who do not have proper know-how how to diagnose the case. But sooner or later, they are diagnosed. | Overcrowding does not lead to no diagnosis  Overcrowding hinders diagnosis process and slows it down  Patients are first assessed by junior doctors who lack knowledge about MD  Patients consult senior doctors after a long wait and senior doctors are short on time |
| Interviewer: And do you think there's a section for the assessment of mental illness or history taking form? |  |
| Interviewee: Not in OBGYN | Absence of screening protocols in Gynae ward admission form |
| Interviewer: Should it be there? |  |
| Interviewee: Yes, ofcourse |  |
| Interviewer: And, any monitoring protocols present at the moment? | No pro-active monitoring tools used |
| Interviewee: No |  |
| Interviewer: Should they be there? |  |
| Interviewee: Yes |  |
| Interviewer: What about referrals? |  |
| Interviewee: Referrals… We do refer for patients | Patients are referred to specialists |
| Interviewer: Alright, and how does it work? How does the referral system work? |  |
| Interviewee: The referral system in our hospital is very simple. Like on patient basis, you have to write it down and send it to the respected department and if the respected department has the, like, found that the patient it significant for admission they admit , and they mention on the antenatal card or they just like attach to it the case summary. The medication she's usually taking and they let the patients know, like they have to mention it. They give awareness to patient. | Referral is not centralized  Case histories are managed manually and all documents are provided to patient  Loss of data |
| Interviewer: And what are the factors that can help protect mothers from maternal depression? |  |
| Interviewee: The factors should be she should have a supportive family, she should have a husband who can actually take care of her and again, the lack of, no not the lack of, but not burdening her. And again, others like unwanted pregnancies. Well, when you ask me about the depression thing, the risk factors na, unwanted pregnancy is one of the things that patients could go into maternal depression because of the repeated pregnancies they have. | Supportive family  Caring husband  Less burden  Unwanted pregnancies can lead to MD |
| Interviewer: Alright… |  |
| Interviewee: Like, she's gravida ten, she has already nine kids at home, maybe because the gender thing like she has all females and the husband want a male thing, unwanted pregnancy would lead to complications and the women can go into this, depression thing, because its not easy to bear with all the kids and take care of the family, take care of the husband and the society and the social gatherings and then patient is suffering from this thing so | Pressure to provide male offspring can lead to MD  Complications due to continuous pregnancies can result in MD  Unwanted pregnancies effect patients ability to deal with day to day requirements and responsibilities, enhancing depression |
| Interviewer: Alright, so do you think, so you mentioned that we can counsel the patient, talk to the patient, there can be support groups, so what, early identification is important for mothers? |  |
| Interviewee: Early identification, yes. But again, that could be achieved if you ask the patients or she mentions to you… | Patients can be identified early if they are aware of their situation themselves |
| Interviewer: Alright, so then that means there should be screening for maternal depression? |  |
| Interviewee: Yes, there should be screening. | Screening is a must |
| Interviewer: And what kind of early treatment or what kind of early interventions do you think can be taken care of once she's diagnosed? |  |
| Interviewee: Counseling, Counseling sessions. | Counseling |
| Interviewer: And have you ever heard of therapies that are used for maternal depression? |  |
| Interviewee: For maternal depression, apart from medical therapy, there is electrocution therapy I guess, but not when she is pregnant, after delivery | ECT after baby is delivered  Medicines – antidepressants |
| Interviewer: Theek hai. And what kind of medical therapies? |  |
| Interviewee: Anti-depressants, I've heard. That's it. |  |
| Interviewer: Okay, and any other comments you'd like to give? |  |
| Interviewee: No, that's it |  |
| Interviewer: Alright, thank you so much for your time. |  |

| Dr.Shafaq  *Briefing provided - verbal and written consent obtained* | |
| --- | --- |
| Interviewer: So ma'm my first question to you is - can u please share your understanding of the health problems usually faced by mothers during maternal depression? |  |
| Interviewee: Well they have a lack of support specially if there are from a very lower social economic status that we deal with mothers here at JPMC, specially , lack of support at home for the kid , for themselves and also social economic support for them in rearing up the of the child | Lack of support in underprivileged communities  Lack of social economic support for child and mother |
| Interviewer: What kind of specific health problems do they face because of this? |  |
| Interviewee: They have a lack of availability of healthcare professional support to them specially after they deliver , if they are fortunate enough to deliver in a hospital, a majority of the women they don’t deliver in a hospital , they go to some untrained skilled or untrained or skilled work attendance but they are not proper doctors, so they are not equipped to deal with these problems, so if they have an access to hospitals they will mainly go there for their bleeding issues or something related to their partum period but not for their mental health state. So they somehow cannot put a finger where they really need help and also the health care set up in our society that do not regard mental health as a very major health issue so that's how they are missed….and that's how we don’t see the real need of providing them with something that they really need at that time. | Lack of availability of HCPs within underprivileged areas  It’s a blessing to have means to deliver in a hospital  Most LES women give birth under the supervision on unskilled attendants  Unskilled attendants are not equipped to deal with MD  LES women only visit hospital when physical ailments become life threatening  Healthcare system doesn’t regard mental health as an important issue    Need for MD counseling is not very prominently displayed by sufferers |
| Interviewer: So can you maybe describe a case that left an impact on you? Someone suffering from maternal depression....it can be before the baby was born and can be after the baby was born? |  |
| Interviewee: Well there are many cases, I can’t put a finger on any one of them but there are many cases because of the gender of the child, sometimes there's a lot of society pressure to have a male kid  to have a male offspring due to many issues some time they have female issues dont they have a male issue they are under pressure husbands going to marry again or husbands going to leave her and also the more children they have the lack of bonding they have with their children so thats they feel alienated with there off springs so maybe that’s a contributing factor but there have been where they have left their children abandoned their children or failed to take ownership of their children once there was a woman who was ready to sell her child because she had a female issue with a person and that happened when i was in New York, and the child was still in the uterus and not born and she determined the sex through ultra sound and she came to know it was a female kid and she was very depressed upon hearing that news and there was a woman sitting there and she offered she can buy the kid from her because she was childless and that woman agreed to sell her unborn child to that person because she was a female child. | Societal pressure due to child’s gender can lead to depression  Divorce threats for not delivery male offspring from husbands can lead to MD  Lack of bonding amongst mother and child due to large number of children can result in MD  Failure to take ownership of child or abandoning her child, due to gender, can lead to MD |
| Interviewer: Okay, so urmm can you define your understanding regarding maternal depression? |  |
| Interviewee: It's something which urmm see there are three kinds, there's daily blues which is normal after baby is born, the mother is depressed sad, sometimes she cries, but the bonding's effect, she doesn't want to harm the baby. Then there's maternal depression in which she has no bonding with the child, she's crying she's depressed she'd careful she's not taking care of her and her child like she should've been. And then there's maternal psychosis where she harms the child, she'll even throw the child kill the child. So yea.. | Three kinds of MD  Baby blues are normal after baby’s birth  Baby blues: Mother cries, bonds with baby and does not feel the need to harm her child when experiencing baby blues  MD: Lack of bonding with child, Crying spells, Lack of self-care  Psychosis is when mother harms the child and tries to kill the child |
| Interviewer: So do you think it exists? |  |
| Interviewee: Yea of course. | MD exists |
| Interviewer: And what amount of burden does society have on because of it? |  |
| Interviewee: As in urmm what perimeter do you want me to judge? |  |
| Interviewer: How much burden does this have on our society overall? |  |
| Interviewee: Maternal depression has a significant impact on society because urmm the woman is not participating in the day to day activities, so due to her lack of participation the child has to be looked after by the other parent the father, any other member of the family which will affect their day to day activities so you know it'll interfere | Significant impact on society  Mother is unable to participate in daily activities  Family needs to look after child, affecting their daily routines |
| Interviewer: So considering it has such a huge impact, what do you exactly understand by prenatal and post natal depression? |  |
| Interviewee: Prenatal depression, I don’t have such an understanding of it, but post natal depression, yes, because postnatally when the child is born and when she sees her kid being there and if she has a lack of bonding with the kid and that is leading to abandoning the kid, so kid naturally, or the responsibility of the kid naturally falls on the family members so you know there's a certain urmm, if, if she has the support at home well and good, but if she doesn't, then eventually the part of society, where she's living in gets involved. So urmm, I think urmm, since there are no special child security ervices available in Pakistan, so naturally the kid gets neglected. But if there was in case the kid would've gotten a better chance in anything. Also there have been urmm cases where urm the kid has been abandoned by their parents at some orphanages due to that, and that the kids are not really orphans so that increases their burden as well | Lack of understating for prenatal depression  Abandonment of child  Lack of support by family leads to issues for child  No special child-rearing helping services available  Child is neglected  Society is burdened when children are left at orphanages by families due to lack of resources to look after them |
| Interviewer: Alright. So what exactly are the symptoms of maternal depression? |  |
| Interviewee: Urmm like I've mentioned earlier,  the mother would be very urmm there would be lack of bonding with the child, she'll be really depressed, she'll feel sad, she'll be tearful and she'll be insomniac, she wont be looking after her kid like she should've been, she wont be feeding the kid properly, she would be neglecting herself aswell, and she'll be guilty for not being able to bond with the child aswell | Lack of bonding with child  Sadness, teary, insomnia  Lack of self-care  Increased guilt due to inability to bond with child  Child’s dietary intake is affected |
| Interviewer: Alright, so what is the time duration for maternal depression? |  |
| Interviewee: It could last for post-partum; it could last for three months. Yea three months, postpartum | Post-partum MD can last for three months |
| Interviewer: And, any idea about prenatal? |  |
| Interviewee: No. I dont think there would be a prenatal, urm prenatal means before conception so before conception urm the depression wont be because of the kid, it would be because of the other circumstances and it would be time to conceive for some time, it could be due to infertility, it could be due to other things, so urm I'm not really clear about the prenatal | Prenatal depression doesn’t exist - Before conception, mother cannot be depressed because of child  Lack of clarity about prenatal depression  Infertility can lead to general depression, putting women at risk if she conceives |
| Interviewer: Alright. So you do you think a mother can be possibly depressed while she is pregnant and is not giving birth? |  |
| Interviewee: Yea she can be, ofcourse | Mother can be depressed anytime during pregnancy |
| Interviewer: Because of the birth? |  |
| Interviewee: Urmm no not because of the birth, because of the other factors aswell |  |
| Interviewer: Okay… |  |
| Interviewee: Like major, urm like many things causing her depression in urmm, in when she's not pregnant. Could be circumstances, social circumstances, could be economic circumstances, it could be anything | Social and economic circumstances can depress mothers during pregnancy |
| Interviewer: Okay. Let's suppose a mother did get pregnant, do you think during the nine month period she's pregnant can she possibly be depressed because of the birth? Because of the child within her? |  |
| Interviewer: Yes ofcourse. |  |
| Interviewer: What would really make that happen? |  |
| Interviewee: In our society, it could be the gender of the kid. It's major factor for determining her depression, also because of the lack of support she has and the coming economic requirements coming from the kid, also maybe urmm she didnt urmm it was an unplanned pregnancy and she didnt want to have that kid and you know | Child’s gender can lead to depression during pregnancy  Economic variants, unplanned pregnancy, lack of support can result in depression during pregnancy |
| Interviewer: Alright |  |
| Interviewee: So yea |  |
| Interviewer: Alright. Amongst mother, who is at most risk for developing maternal depression? |  |
| Interviewee: Urmm people who are addicts, like who are really young, really young mothers who have had a history of depression amongst themselves urmm before they were pregnant. Also umm people who donot have a urmm support system basically or somebody who can, not not family person but even friends, urmm also working mothers I guess they feel very guilty leaving the child and going to work so | Addicts are at high risk amongst mothers  Young mothers with a history of general depression  Guilt leads working mothers into depression  Unfavorable support system |
| Interviewer: Alright, so what can be the certain risk factors? If I continue to ask you these are three major factors but what other factors would there be leading to maternal depression? What if she has the social support, she's not a working mother, urm what would still be sort of trigger her and get her to be depressed? |  |
| Interviewee: Maybe, also because urm see its something natural because its something we fear to recognize as a medical problem aswell. People do have such tendencies like they do have pressure. If they are depressed before they can be depressed after the child is born. So yea, that can be the background stimulating factors | We fear to recognize MD as a medical problem  Past history  Inability to deal with stress and pressure |
| Interviewer: Alright, so how do you think we can manage maternal depression? |  |
| Interviewee: Well there should be some peer support groups like they have all over…also hospital based anti-partum classes should be held for mothers in what we expect and how to deal with it and how what servies are available. There should be urm from the psych services so utmm counseling talking | Peer support groups  Hospital based anti-partum classes  Classes regarding awareness for mothers about expectations and available services  Counseling |
| Interviewer: Counseling and talking, right. So what are the short term and long term implications of maternal depression? On mother the society the children whoever you think will be impacted by it? |  |
| Interviewee: Urm on society per se ofcourse the mother is depressed and wont be able to raise up her child accordingly like his education urm his or her education will suffer, his or her upbringing will suffer and the connection that he or she will have with the family will suffer. The mother in hereelf as a person would probably be not be as productive as she could've been and urm eventually this all adds upto feeling of alienation from urm society from the family and everyone around so that may lead depression you know to the child aswell | Child’s education and upbringing can suffer  Child will be unable to bond with family  Mother’s productivity levels will fall  Feelings of alienation affects suffering mothers |
| Interviewer: Alright. So do you think, you mentioned that the schooling of the child will affect how the child is brought up. But do you think as an infant the child would be effected? |  |
| Interviewee: Yes, ofcourse |  |
| Interviewer: How? |  |
| Interviewee: Health like if he's not been taken care of properly and is neglected, there could be a danger to his life, he even maybe not like you know urm urm if he's not been cared properly taken care of, his health would suffer, he would require multiple hospital visits, multiple admissions sometimes, so that would be another burden on hospital and society aswell | Child’s life can be in danger due to neglect  Child may require multiple hospital visits and admission if not taken proper care of  Societal burden can increase when preventable higher levels of care are required |
| Interviewer: So could you please shed some light on what did you mean by the child taken care of properly?  What does the word properly mean for you? |  |
| Interviewee: Urmm like, like you know, if urmm if he's not been fed on time, if he's not been given attention and you know his milestones are not being reached on time and is being neglected, so that's how it is | Inability to reach child’s milestones on time can affect child’s health |
| Interviewer: Okay, will the family members be affected? |  |
| Interviewee: Ofcourse |  |
| Interviewer: How? |  |
| Interviewee: They will be affected because child is basically a part of the family, its part of the functional unit they have. So you know, upon urmm on the father he wont be happy seeing his child, you know, being neglected like that. Also they would be concerned about the mother aswell, so that would again lead to their urmm being nonproductive in certain areas because of the urmmm, because they have to spend some additional time with the urmm mother and the child aswell, so... | Family unit will not remain functional  Father will not be happy since the child will be neglected  Family can fear for mother’s health  Accountability to spend more time with mother and child increases |
| Interviewer: So how are care givers and associated partners responsible for the mothers affected? |  |
| Interviewee: The care givers are affected because of maternal depression. Urmm see, they'll, they'll have multiple risks from the mother aswell. She'll probably blame her depression on her physical symptoms which are normal for any urmm mother for post partum. So she'll be coming back to hospital and it'll be a burden for the hospitals aswell. Also, the time of the doctors. And the caregivers, probably the care giver would be, urmm see, apart from being mentally disturbed, he'd had to bring the mother back to the hospital, he or she who ever's taking care of the mother and urmm you know the utmm not all hospitals are free so yea thay would be a financial burden on them aswell. And urm taking time out from their work and taking the woman out, and also if, if they're bringing the woman back to the hospital, thinking what's wrong with her and why she's not feeling well and urm they'll probably try to blame the hospital and the doctor for not treating her well, when when you know it should've been depression that should've been look after in the first place. | Mother continues to get physical checkups wasting financial resources and time of doctors and her spouse  Blame falls on doctor and hospital when women continues to suffer after multiple checkups for physical issues  Financial burden on pro-bono hospitals  A mother is treated for depression when no other ailment justifies her wavering condition, worsening her condition |
| Interviewer: Alright. So in your opinion, how is maternal depression managed in Pakistan? |  |
| Interviewee: In Pakistan there's no focus on mental health, per se. Let alone maternal depression, we've been failed to recognize mental health which is very essential part of health program that we have. But yes, there are centres urmm urmm and also patients fail to report, they they fail to consider its a mental issue because its not in our society, be it maternal or nonmaternal. But if they do report to us even as health care professionals, we just shrug it off, we dont give it much thought. Sometimes we send them to a psychiatrist that they're hallucinating or they're at the end stage if their urm depression, or if they're suicidal. Urm but in the initial stages where they should've been really taken care of, nobody really picks it up at that point. So I think there should be, we should have some collaboration maybe in the psych department for this, or maybe there should be more input from them in our department regarding this so that we pick up the signs of depression being present in the mother earlier than you know | No focus on mental health in Pakistan  MD is not even counted as an issue  Patients do not report MD  HCPs usually shrug off symptoms when mothers report it  Referrals are used in severe cases when mother starts to hallucinate or becomes suicidal  Not diagnosed or treated in its initial stages  A working relationship should be established between the gynae and psychiatric ward to provide holistic care |
| Interviewer: Alright, so do you think hospitals use tools to diagnose maternal depression at all? |  |
| Interviewee: They do, yes |  |
| Interviewer: What kind of tools? |  |
| Interviewee: Well, there's urmm very little score but that's not just for maternal depression, there's a New York, no, Minnesota I don't remember Minnesota scale but it's not being used regularly by the hospitals, uhh specially in the hospitals I have worked in be it AKU, be it JPMC, uh but yes from the, from the OBGYN perspective that is, I don't know if psych team, if they're fortunate enough to get referred to a psych but not here. | Sometimes hospitals use scales  Most private and public hospitals in Pakistan don’t use any scales  Most scales are used only in psychiatric ward  Patients are assessed via scales only if they are fortunate enough to be referred to psych ward |
| Interviewer: Is it ever a part of the initial screening process when a mother comes in telling you that she's pregnant? |  |
| Interviewee: No | MD screening is not conducted with mothers |
| Interviewer: Nothing about, let alone depression, generally her mental condition? |  |
| Interviewee: No | No screening for mental health is conducted with mothers |
| Interviewer: Have you recently felt the need to explore that? |  |
| Interviewee: Yes |  |
| Interviewer: Do you ever explore it? |  |
| Interviewee : Uh yes uh, when, see, yes I do but I don't employ a scale in it, uh I do try to hear I out, I do try to refer her to a psychiatrist but then they can't follow up and with the burden that we have in the hospital we usually don't get the time to, you know check if the patient is doing well or, you know follow-up with her. | Hearing the patient out is something that doctors do  No scales are employed during screening  Referrals to psychiatrists are made if needed after talking to patient  No follow-ups are carried out |
| Interviewer: So you've never used any screening to? |  |
| Interviewee: No, for the maternal depression, no |  |
| Interviewer: And when the mother comes in, how much time do you give per patient? |  |
| Interviewee: Uhh, ten minutes uh, the most fifteen | At most gynecologists can only give 10 minutes to a patient |
| Interviewer: So, do you think it can be treated or managed? |  |
| Interviewee: Yes, of course | It is manageable and treatable |
| Interviewer: What would be the different forms of treating it or managing it? |  |
| Interviewee: Counseling, educating it about her and educating the people around her about it, educating doctors especially about it, educating the health-care staff and the personnel about it and, well they should be more social supported for the mothers like as such and there should be a number so that we can call and you know…and involving more people so that she knows that people are there to help And specially for her to recognize that this is a problem which is normal and can be sorted it's not something which is going to be a taboo or a stigma or anything | Counseling, educating mother, educating family and other caregivers, educating HCPs  Hotline numbers should be provided to mothers  Creating of a strong support group  Normalizing the problem  Destigmatizing the phenomenon |
| Interviewer: So, if you think there are treatments that exist, do you think they're in Pakistan? |  |
| Interviewee: Yes |  |
| Interviewer: Where and how? |  |
| Interviewee: AKU has a very good, there's Doctor Esaani I guess, he's really good but there's the availability of the psychiatrists dealing with maternal depression is sort of towards the lower side , they are available but people do not about them, the awareness of, their availability is no as such. | Treatments exist privately  Only a few private specialists deal with MD  People do not know how to reach out to the specialists |
| Interviewer: And if this treatment exists and people do know about it, do they seek help? |  |
| Interviewee: No |  |
| Interviewer: Any reasons that you can think of? |  |
| Interviewee : Because, see mostly people who seek help are there because either they are the in stage or either their health care professional like us will be referring them to another health care specialist also because of the stigmas attached to mental health. | Due to stigma, people only seek help when referred to a psychiatrist and if case is severe |
| Interviewer: So what can be the best approaches or the best combination of approaches that can make sure that we can engage mothers with depression or sort of help them out? |  |
| Interviewee : We can have classes with them, we do teach them exercises how to give bath and what to expect, this should be part of those classes also there should be availability of such screening tools, when they're being seen postnatally specially and also there should be availability of mental health services in the opg 1 clinics as well, so that you know they don't have to travel to other departments and you know yeh bhi hota haina nafsiyati hospital jaa rahi hai woh waala stigma naa attach ho uss seh So help is there available if they need it. Also support groups, yes, support groups should be formed… | Classes for mothers should incorporate a psychological component  Exercises to prepare mothers to deal with the new responsibilities  Mental health services should be available in Gynae ward to help patient avoid stigma  Support groups can help |
| Interviewer: Since there are all these available treatments and as you tell me that they're still there in Pakistan in some places alright, so what are the barriers that are stopping these mothers from seeking care? |  |
| Interviewee: The stigma attached to it, the resources, the lack of appreciation for the mental state and also there are, what they don’t think of mental state as their priority, you know until and unless it's significantly affecting their lives like they're going suicidal or depressive enough to harm their babies, nobody is going to report them. | People don’t seek help cause of Stigma  Acceptability and tolerance towards mental health issues  Lack of reporting unless its significantly affecting lives; self-harm or harm to others  Lack of resources stop people from reaching for help |
| Interviewer: So do you think religious beliefs have anything to do with them? |  |
| Interviewee With maternal depression? I don't think so, personally I don't think so. | Religious beliefs play no role |
| Interviewer: How does a mother or a patient perceive maternal depression? |  |
| Interviewee: She will probably be not walking, she would probably be very guilty in not being able to take her child, she will be depressed because she's not taking care of the child as she should've been and there would be a feeling remorse and harm and that will be effecting her day to day activities | Guilt  Inability to take care of child  Lack of motivation to walk or carry out daily tasks  Feelings of remorse  Thoughts of self-harm |
| Interviewer: Okay so what do you role think the medical educational framework of Pakistan plays in the management of depression? |  |
| Interviewee: Medical educational, urm well, if you talk about urm being aware of this thing since our college education then there's a zero lack of awareness there then there if you talk about then urm their house jobs the residencies, then again there's a lack of awareness so there should be framework for the postpartum depression and it can be anywhere in the medical department in the surgical department and of course she will be in the OBGYN department as well and even if she goes to psych department they'll give her moves and all they will not locate the exact cause of it so that's just because you're not aware of it and they don’t regard it as a serious entity in itself | Zero awareness about MD during medical education and residencies  A framework should be established for MD  Framework should be available to all departments which a mother can potentially visit  MD is not diagnosed for its not regarded as a serious ailment |
| Interviewer: So am I right and understanding that the medical department and nursing has not shed any light on it and is not.. |  |
| Interviewee: No it has not, yes you're right | Awareness about MD has not been part of curriculum specifically |
| Interviewer: Alright and as you said earlier there's no on job training either? |  |
| Interviewee: No | No on job training |
| Interviewer: Okay, and have you ever attended workshops and any kind of trainings which talk about it? |  |
| Interviewee: No | No workshops |
| Interviewer: Your practical experience where you were maybe forced into it? |  |
| Interviewee: Yes |  |
| Interviewer: Urm if you could tell me a bit about it? |  |
| Interviewee: There are patients who are aware of it and they come to us and we have a collaboration and we get to know this exist and we are told, there's been cases she was suicidal, she threw her baby out, ee had a collaboration but that was when we were forced to do it, see it wasnt coming, we didnt identify her in the coming stages so that was us I guess | Some patients are aware of it  When patients complain of symptoms, some doctors are forced into paying attention and learning about it  Lack of knowledge leads to late diagnosis |
| Interviewer: So you think self-efficacy is an associated factor to this day? So you, as a person think - self-efficacy plays a role when it comes to maternal depression? |  |
| Interviewee: Yes | Self-efficacy plays a major role |
| Interviewer: How? |  |
| Interviewee: See, if I I would lack the skills or the knowledge or the awareness of recognizing it, then how will I manage a patient or leave a patient where she should be? So if I'm the one giving empowerment or a tool or a thing to deal with this then probably I can do better with the patient | Skills, knowledge and awareness of HCPs help in diagnosis and treatment  Empowered HCPs can use tools to deal with MD |
| Interviewer: Alright, so do you think urm personal responsibility plays a role in this? |  |
| Interviewee: Yes |  |
| Interviewer: How? |  |
| Interviewee: Well, um taking care of a patient is itself physically mentally emotionally however, it entails our profession, it entails my job, so yes it my responsibility to take care, to be able to take care of her, and urmm yea | Call of duty entails that doctors look after patient physically, mentally and emotionally |
| Interviewer: And you think compassion and empathy plays a role in this? |  |
| Interviewee: Yes, it does, ofcourse. |  |
| Interviewer: How? |  |
| Interviewee: Because see, urm at the end of the day, she's a mother she's urm helpless soul you know. She's responsible for the kid if you take it from the kid's point of view, he or she deserves the best because you know, every kid is precious to us. Also the mother herself you know there's not muc we need to give to her, its just a few sessions of talking counseling telling her its going to be normal. That's going to bring a change in her life improve her life and her mental health, she'll feel mentally well and you know she can enjoy the experience she has in her motherhood and obviously it'll be better for the kid aswell | MD makes mothers helpless souls  Kids lose out on getting the best care  Talking and counseling can do wonders  Motherhood is an experience that should be enjoyed |
| Interviewer: And do you think that urm you'll be comfortable talking to a mother about it and urm feel confident to talk about it? |  |
| Interviewee: Yes urmm urm not exactly but yes to an extent. Because see, right now I'm at the end of my training, I've seen cases but you ask me this question initially and the initial period of my training, maybe not. But since I've seen cases, dealt with them referred them, studied for them so maybe now yes i can say that. | Senior doctors are more comfortable in talking to mothers about all their problems  Practice helps build strength to discuss all issues with sufferers |
| Interviewer: So earlier you mentioned that there's a loss of follow up so even if it is diagnosed, have you ever felt that you want a follow up or you followed up and made sure that the patient has gotten the kind of treatment required for her? |  |
| Interviewee: Yes, I have |  |
| Interviewee: And what happened in those cases? |  |
| Interviewee: They were fine because they used to come up to us specially at AKU because there's a written document and thing going on and you know the stations are sort of fixed so we get to see the patients again and again and you know they're easy to follow up there soo yes they were doing fine they were identified as being blues not in state of depression and when they used to come after six weeks for post natal depression i mean they should, usually they were better, so.. | Decentralized systems lead to a flawed follow-up system  Most times if taken care of, it ends at the stage of baby blues |
| Interviewer: Do you think the general practitioners should be the first line of support? |  |
| Interviewee: Yes, I do | GPs should be first line of support |
| Interviewer: And you think it is a possibility given the routine to gynecologist and people usually have? |  |
| Interviewee: No | Routine for gynecologists don’t allow time for diagnosis |
| Interviewer: So do you think, urm what would be the practical solution to this? |  |
| Interviewee: Teaching, um see the practical solution to this would be implementing in the antenatal checkups, visits, the health care professionals dealing with her at the time of birth the health care professionals dealing with her when she's coming to them for a checkup of postnatal. This should be a part of the routine examination. And also, if she's going to the urm, she should be offered brochures or numbers to contact if she feels she needs to talk about her, you know. Social support groups should be there | Teaching the masses  Incorporating it within antenatal checkups and regular checkup visits  Information brochures should be provided to mothers  Social support  Hotline numbers |
| Interviewer: So who should be generally responsible for doing this? The classes, the education support teaching her, telling her about it? |  |
| Interviewee: Urmm it should be a combined effort from the hospital, from the Ngos. Because you know hospital cannot partake in social support, its not coming from a society | Different forms of awareness with support from NGOs |
| Interviewer: Alright. So do you think the gynecologist the mother is visiting in most cases we see she visits her doctor whom she really trusts? Do you think the gynecologist should be responsible for doing this? |  |
| Interviewee: Ofcourse. The hospital entails  the gynecologist and psychiatrist and you know the health care profession it has | Gynecologist and all other doctors that a mother visits are responsible for her diagnosis |
| Interviewer: So would I be right in understanding that they should all be in the same place in one unit where the mother goes through different ones? Or should it always be one person she talks to and then be referred? |  |
| Interviewee: No, these facilities should be available to her in one place and she should be able to access them whenever at her free will but if that entails her in going round the city, it would sort of make it more difficult and probably she would not take help | All facilities should be available to mother in one place to increase rate of uptake |
| Interviewer: Alright. So should it be mandatory for her to visit each urmm.. |  |
| Interviewee: No not mandatory, not mandatory |  |
| Interviewer: Alright |  |
| Interviewee: But you know she should know there is an option available. Right now they dont even know there's an option available or if there is an option. | Different treatment options should be made available  Not all things can be mandatory  Currently there no options available |
| Interviewer: Alright, so what factors play a role in the diagnosis of management of maternal depression in a hospital setup? |  |
| Interviewee: There should be screening tools, there should be enough time given to a patient for a successful visit. If a patient is complaining about, like not being able to sleep well or not taking care of a child well, or feeling depressed, those should be taken very seriously because we rush them off keh nhi aisa hota hai theek hojayega so these toh you know should be noted you know maybe.. | Screening tools should be used  Time should be sufficiently provided to patient  Patients complaints should be paid heed to  Patients problems should not be shrugged off and neglected |
| Interviewer: So if I'm right and understanding, you people have a history form used for each patient, |  |
| Interviewee: Yea |  |
| Interviewer: Right, so urmm |  |
| Interviewee: Yea there should be incorporation of those for OBGYN or postnatal depressions | History form should incorporate a component of MD |
| Interviewer: So at the moment there's nothing in that? |  |
| Interviewee: No |  |
| Interviewer: Okay and are there any monitoring protocols that are used to see if a mother is going through depression? |  |
| Interviewee: No | No pro-active monitoring tools are available |
| Interviewer: What happens when it comes down to referral? How would you refer a patient to psychiatrist if you know she needs to.. |  |
| Interviewee: Urm we will just write it down for her that needs to see a certain doctor at a certain department and she can go and follow up her doctor there and get an appointment from him or her | Patient has to visit the doctor she is referred to  No systematic way is present to ensure referrals are used |
| Interviewer: So there's no specific referral to see if she's following up or.. |  |
| Interviewee: No |  |
| Interviewer: Or come back to you? |  |
| Interviewee: No no no, there's no services available as such | No system to check if follow-up is carried out |
| Interviewer: Alright, so what what are the factors that can help protect the mothers from maternal depression? |  |
| Interviewee: Urmm talking about it knowing that urmm ofcourse there's something they cant avoid it specially because they are depressed prenatally and urmm also not avoid it but they should brace themselves for it and they should be aware of it. Basically awareness and knowledge to her and health care professionals to the people in her family | Talking to mothers  Confirming that it is not avoidable without help  Equipping them with means to deal with it  Awareness and knowledge amongst society and HCPs |
| Interviewer: Alright. So do you know any therapies that can help mothers during this phase? |  |
| Interviewee: Yea, counseling talking and urmmm goo health probably. | Counseling  Talking |
| Interviewer: Any specific therapies that you've been a part of or heard about? |  |
| Interviewee: No I've not been a part of this | No idea about specific therapies |
| Interviewer: Alright, urm any specific comments that you'd like to give? |  |
| Interviewee: Urmm, regarding this? Yes |  |
| Interviewer: Regarding the overall topic we discussed about. Anything you think I didnt ask but is very important for me to know as a researcher? |  |
| Interviewee: Not really, I think it was very comprehensive |  |
| Interviewer: Alright |  |
| Interviewee: And maybe you, urm the suggestions based on so it, it made me realized that I should make it a habit of asking her general status, her mental health because whatever we do is focused on the physical health of the mother on the physical symptoms she's coming up with | More focus is on physical health |
| Interviewer: Thanks. Thankyou so much for your time. |  |

| Dr.Tanzila  *Briefing provided - verbal and written consent obtained* | |
| --- | --- |
| Interviewer: Can you please share your understanding of the health problems that are usually faced by mother during maternal depression? |  |
| Interviewee: Like, regarding depression? Like they have some issues personal and family issues and social like financial burdens that you know, trapped them to depression. | Personal issues  Family issues  Social issues such as financial constraints |
| Interviewer: So what kind of health issues will they be facing? |  |
| Interviewee: Like if they are financially compromised they are unable to have proper meals, they are anemic or maybe they have grand multiple children that results in less intake of good food, good meals, healthy diets, and like they become weak. | Poor - No proper meals leads to Anemia  Poor - Distribution of food amongst too many mouths  Weakness |
| Interviewer: Right! So can you describe a case that might have left an impact on you regarding maternal depression? |  |
| Interviewee: Ah! One of the case was postpartum depression but, like we come across with patients usually those who are very sick, and ill with low social economic status they have anemia or some multiple disorders, so their health and their present status or specially the death of the child if the current pregnancy lead to the IUD baby so this all dragged them to depression and psychosis. | Case of postpartum depression encountered  LSES, severe sickness, anemia, multiple disorders, death of child, IUD babies are some factors  Major trauma results in depression or psychosis |
| Interviewer: So in that case do you agree that it exists in our society? |  |
| Interviewee: Yes it exists but sometime the perception is not true like the most of the families don’t believe that its depression and don’t care and so it is neglected. | Depression is neglected  No associated importance with pregnancy  Existence of depression is denied |
| Interviewer: So um what kind of burden does it have on the society? |  |
| Interviewee: If the mother is not well she may not be able to take care of the baby, so like ultimately the family needs a maid or they you know they help her doing things and stuff and suppose that if she is very sick, the husband may need to get over with the working so ultimately lead to burden on the society. | Lack of care towards baby  Family needs to look after child  Maid needs to be hired  Husband may need to leave work in severe cases |
| Interviewer: Right so can you please tell me what do you understand by the term pre nature and post nature depression? |  |
| Interviewee: Prenatal depression is like blues, baby blues, it usually occurs in the females like lasting for seven days, it’s just, it is basically the fear of delivery and taking care of the baby | Baby blues is prenatal  Lasts 7 days  Triggered by fear of delivery and new responsibility |
| Interviewer: So it happens before or after the delivery? |  |
| Interviewee: It happens after delivery but sometimes you know it starts before you are going into labor and you worried about what happened so and so. | Occurs after delivery  Sometimes triggered by fear of labor |
| Interviewer: So this is prenatal depression? |  |
| Interviewee: It is, and sometimes it is, if the maternal condition is not well if the like there are certain complications like if the mother is hypertensive or diabetic and she has to change the routine like i am really diagnosed with diabetes and i am pregnant aswell so this lead to depression you know when you cut off things which you were regularly eating and you have like ah, it’s a good thing that i am pregnant but still this diabetes the recently diagnosed diabetes lead me to depression and i feel like okay this is the bad thing, so like my pregnancy is not a good thing, so all this lead to depression, prenatal depression. | Complicated conditions lead to early depression  Diabetes, hypertension trigger depression  Changing eating habits because of complications results in depression  Fear of something bad happening because of medical condition activates depression |
| Interviewer: So i am right to be assuming that you are pregnant right now? |  |
| Interviewee: Yes |  |
| Interviewer: right, so what are the symptoms of maternal depression that you feel - that you have been feeling it yourself? So what are the symptoms? |  |
| Interviewee: Symptoms are the feeling of loneliness, tiredness, fatigue sometimes like crying for no reason these are the things i am come across | Crying spells  Loneliness, tiredness, fatigue |
| Interviewer: right. So what is this time duration of maternal depression? |  |
| Interviewee: Both? |  |
| Interviewer: Both. |  |
| Interviewee: Postpartum depends on the severity like blue baby blues like occur for seven days or so and it usually mostly occurs in all females or so 90 percent or 80 percent but sometimes it relieves in few weeks it takes few weeks, to adjust like one of my friends was in postpartum depression only because of caesarian section, feeding her baby, so she took around one and a half week or so to adjust that, okay now, this happened and i am unable to deliver the baby vaginally, so I need to have a caesarian section - than she moved on | Postpartum is baby blues  Baby blues can last 7 days to two weeks  80-90% females suffer  Caesarian, breast feeding can be a reason |
| Interviewer: Right um so as you mentioned earlier - just trying to understand it further more, so period apka wo hota hy jab apka baby deliver hony wala hota hy and the mother is in the delivery room or is it the ninth month |  |
| Interviewee: Nai….antenatal depression can occur at any time like before the baby born | Antenatal can be any time before birth |
| Interviewer: Okay, so it can be during the nine months of pregnancy |  |
| Interviewee: Yes… and specially it occurs in the first trimester. | Occurrence of antenatal is high in 1^st^ trimester |
| Interviewer: First trimester? |  |
| Interviewee: First trimester main bohut zada hota hai, mood swings and changes and alternations in the body, physiology but the second trimester is the safe trimester and again in the third trimester when the baby’s weight is gaining more changes are more or the mother is near to delivery so depression may occur more, commonly what i feel like is more common in first trimester | Mood swings, alterations in body, physiology increases chances of depression  Common in 1^st^ trimester  2^nd^ trimester is safe  Can occur in 3^rd^ trimester as more changes occur |
| Interviewer: What are the risk factors of maternal depressions? |  |
| Interviewee: Previous history of depression or some antidepressant intake or some family history | Past history of depression, Past family history  Intake of antidepressants |
| Interviewer: What kind of family history |  |
| Interviewee: What like in my like background, my friend she was a victim of postpartum depression, her sister is now facing postpartum depression and her one the sister i guess in antenatal depression as well even matlab she attempted suicide. | Women can attempt suicide when depressed  Runs in families sometimes |
| Interviewer: Alright... |  |
| Interviewee: matlab she attempted suicide although she did not commit it but she was like in a very very severe depression, what i feel like, okay maybe it is related to some family issues |  |
| Interviewer: So it can be family history or personal history…. |  |
| Interviewee: Hmm. |  |
| Interviewer: Right so how can it be managed? |  |
| Interviewee: With the good behavior and sympathy, and like providing good environment to the mother and comforting words… all these can help her out | Good environment for mother  Strong support  Sympathy for mother’s condition  Comforting words and good behavior |
| Interviewer: Right! So what are the short term and long term implications of maternal depression? |  |
| Interviewee: Short term implications like if the mother is in depressive phase she may not be be to perform her daily activities and you know when i am in depression, i am unable to come on the next day | Inability to perform daily activities |
| Interviewer: Right |  |
| Interviewee: because i can not sleep well, so this may disturb the daily activity so if like unlike me a mother is not working she may be unable to do the daily routine work, like the daily routine family work, like if she is having some other babies aswell she may not be able to take care of them easily and properly. | Lack of sleep  Inability to do household chores and office work  Incapability of looking after child properly and easily |
| Interviewer: Right |  |
| Interviewee: Tou negligence hojai gi unky side pr | Negligence towards life |
| Interviewer: Sai theek hy so ah, you know that you mentioned that there are days that you don’t want to come, so what really happens, why can’t you come to the work? |  |
| Interviewee: So my depression is related to my newly diagnosed disease, the main point, the second point is that when i got pregnant, all of a sudden my husband has to move, so when he was not here i just got to know that i am pregnant, these were the two most terrible things that happened, my diabetes and the absence of my husband and you know it was making me depressed a lot and secondly working on emergency, i have to do my calls and i work alone so absence of surrounding friends and my colleagues you know add up to the depression as well | Major emotional stressors  Increased physical distance between spouses  Hectic work schedules  Absence of support from family and friends due to different schedules |
| Interviewer: Right so, just coming to the earlier question a little bit more, you cant come to work because of what reasons? What are the symptoms that don’t allow you to come to work? |  |
| Interviewee: I feel nauseating and tired because of no reason, i don’t have a call like i’d have regular routine and i’d sleep well and i have nothing to do at home, like i want to stay at home despite the fact that i to check my fbs, i don’t get up from my bed for no reason, i feel like tired, so tiring and… | Nausea  Tiredness  No motivation to leave bed |
| Interviewer: So is it k dil nahi krta ya thaky hoy rehty hein? |  |
| Interviewee: Sometimes its like dil nahi krta, you know sometimes i have regular normal routines of day, and you know i am used to of work even harder than i am doing these days but eventually i don’t want to do like my books are nearby and on the table and just don’t want to open them and read like… so bad | Lack of enthusiasm to do daily tasks |
| Interviewer: So do you think it affects the infant that the mother is depressed | Infant is affected |
| Interviewee: Yes. |  |
| Interviewer: How? |  |
| Interviewee: Like if you are not feeling well, I don’t know if these are the myths or so like my mama says that what you feel in the antenatal period it gives an impression to the baby aswell so what i have seen in my niece is that the antenatal period of my sister, presentation of their antenatal period is the representation of their baby as well, like if my sister was in scared mood, my nana was ill while my sister was preganant, she was very depressed because of nana, so like her baby was really irritative to interact, tou meri amma yeh kheti thein k shayad yeh is waja sy istarhan hoi hay.. | Older women preach that child feels what mother feels  Baby’s personality is representation of mother’s antenatal period |
| Interviewer: Right tou apko lagta hy k jo mother k antenatal period mein dekhein gi wohi cheezein apko baby ki personality mein dekhi gein and in the long run kia hoga baby k sath? |  |
| Interviewee: In the long run if like the definitely his or her personality changes because of the intial growth and initial behavior of the baby but but not in long run, your parenting is more important than these behavior | No effect on baby in long run  Initially growth and personality suffer  Parenting plays a role in long run |
| Interviewer: If the mother is clinically depressed, if she is going through maternal depression will it affect the upbringing of the child in the long run? |  |
| Interviewee: I don’t think so because like postnatal period depression is not so long to affect the long run of the baby if she will be depressed for weeks and months maximum, so when she is well tou she may able to take good care of the baby and give good points or whatever she has in upbringing the baby. | Depression does not last so long  Maximum is weeks or months  Mother can catch up with lost years of infant once she is well |
| Interviewer: Right, so are family members affected? |  |
| Interviewee: Yes, as i have earlier told you that if you are in a depressed mood you are unable to do anything, you are unable to sit and talk about it, it is like you don’t want to sit with the family members or talk about their affairs their problems, you are basically in a mute phase, you are not able to share their problem as well. | Inability to interact affects family  Mothers may become mute  Unable to share personal problems either |
| Interviewer: Right so will all care givers be affected, whoever is associated |  |
| Interviewee: hmmm |  |
| Interviewer: Will the husband be particularly affected |  |
| Interviewee: Husband is more important definitely but care from some other family members like those who are living with her help her out but husband definitely is the most important part. | All family is affected  Husband is affected most |
| Interviewer: How is he affected? How will it affect their relationship in the short and long term? |  |
| Interviewee: Like if you are in a depressive mood or you are having some mood swings so if your husband is caring and if he is taking care of your problem and issues and he is considering that yes you are in depression and you need something to…to change your mind like go for a walk or shopping whatever you like, it will make some soft corner in your heart for short term and for long term as well, so like you consider that in my hard time my husband helped me a lot um matlab you should have, um you should consider that you have to help him aswell whenever he is in bad… | Brings spouses close if husband is understanding  Makes wife want to be there for husband in his bad times |
| Interviewer: So you are saying that the implications would be that if the husband is supportive it would impact the relation in positive way? |  |
| Interviewee: Yes |  |
| Interviewer: Alright, will the husband affected in terms of health or in any such form? |  |
| Interviewee: Han, definitely if he is like ahh, financially strong so he may be able to take her anywhere she wants, like this matters a lot, like if you are staying at home and if you are not going anywhere despite the fact that you want to move, what happens on low socio ecnomics status, they can not, they don’t have any entertainment points, they don’t have any chances of entertainment, so like in middle class or higher classes we can go somewhere out or we can… | Financial stability can help create more pleasant distraction |
| Interviewer: Coming back to the point will the husband be affected in a health ah |  |
| Interviewee: This may eventually improve the health, like i feel… | Health improves when one has means to enjoy |
| Interviewer: In your opinion, how is maternal depression managed in Pakistan? |  |
| Interviewee: Very badly, we don’t consider it ahhh usually ahh yeah we don’t consider it… | MD is managed really badly  Not given importance at all |
| Interviewer: So do you think hospitals use tools to diagnose it? |  |
| Interviewee: I don’t think so… | No tools used by hospitals to diagnose it |
| Interviewer: Is it the part of the initial screening process? |  |
| Interviewee: No | No questioning done when mother first visits doctor |
| Interviewer: It is not so um have you ever used a screening tool? |  |
| Interviewee: Never! | Never used a tool |
| Interviewer: Never, have you have felt the need to use one? |  |
| Interviewee: Hmmm maybe, we should have some um um my nephew is doing chartered accounting and when I was in depression he gave me one flow chart and asked me to give the answers to check my score of depression, this could be like this could be in our set up as well but because of low literacy level in our um set up, i guess unable to do this | Low literacy hinders screening due to lack of awareness  Quick self-assessment questionnaires can be useful if importance is established |
| Interviewer: Yeah, so do you think that it can be treated? |  |
| Interviewee: Han han it can be like if, like using score what my nephew was used to of doing, we can do this as well | Can be definitely treated  Laymen can also help in first assessment if awareness is present  Online mediums can be used |
| Interviewer: What different forms of treatment can be used to diagnose and manage maternal depression? |  |
| Interviewee: In antenatal, we usually don’t give any antidepressants as it can have some genetic affect but postnatal… if a patient is in depression so we can give her antidepressants | No antidepressants for antenatal period  Can affect baby if medicines are used  Postnatal mothers can use antidepressants |
| Interviewer: Right…any other forms of treatment? |  |
| Interviewee: Some like woi, good environment, healthy environment, cleanliness, help her out | Healthy environment can be a therapy too  Cleanliness  Strong support system |
| Interviewer: Anything else? |  |
| Interviewee: I think that’s enough |  |
| Interviewer: If effective treatments are provided tou do you think families would seek help? |  |
| Interviewee: Hmm definitely | Families would seek help if effective treatment are provided |
| Interviewer: Are they seeking help at the moment? |  |
| Interviewee: Actually we are not providing any good help to them so, I feel like if we provide them, they will definitely get help. | No effective treatments in place currently  People seek help when means are provided |
| Interviewer: So what approaches or combination of approaches can best engage mothers who are depressed? |  |
| Interviewee: ….ummm good activities | Engaging activities are needed |
| Interviewer: such as? |  |
| Interviewee: Like ahh what my amma tells me recite Quran, like i was used to of, initially i was at so much depression, i used to listen to Allah k name 99 and Surah Rehman daily, so it helped me a lot , helped me a lot, so what i heard from my friend as well she was used to hearing it every time, every day when she was pregnant, so his ah her son is used to reciting it very fast and that’s true, he didn’t learn it yet, the 99 names of Allah but he was used to reciting it very fast like he has learnt it somewhere else, so this is a very good thing and i think this will help us | Activities can be religious or social  Mothers feelings and emotions transfer into child’s personality  Recitation of Quran can help |
| Interviewer: Right…so any other activities that can be done to engage? |  |
| Interviewee: Listening to music | Music and tv can make mother happy |
| Interviewer: Right |  |
| Interviewee: Watching television… that’s what I am doing. |  |
| Interviewer: So what are your perceptions regarding the barriers for seeking help for such a crucial issue? |  |
| Interviewee: Illiteracy, negligence like we all are not considering it, actually the problem like we take for granted | Negligence, Illiteracy, Lack of importance act as barriers |
| Interviewer: Right, anything else? Do you think there is stigma around it? |  |
| Interviewee: Sometimes | It is stigmatized sometimes |
| Interviewer: In what cases? |  |
| Interviewee: In cases like ahh, it gives a really bad impression of the in laws that ahh the, sister in law or the daughter inlaw in suffering from depression give a bad impression like so people are usually not like you know no i am not in depression, this gives a negative impression | Society talks if women suffer  Families feel ashamed to admit that their women are suffering from MD  MD is perceived negatively by society |
| Interviewer: So do you think cultural or religious beliefs have anything to do with it? |  |
| Interviewee: Religious tou nahi cultural hay | No religious associations |
| Interviewer: Cultural kaisy? |  |
| Interviewee: Culture mein aisa na k, family myths hein k inko nahi hoa tha tumhein kaisy hogaya, they are used to of giving their own examples specially mother in laws that we have gone through with this and we have delivered 7 kids or 8 kids and this and this so we were doing all the household work and taking care of the baby and despite we were very healthy and so and so, and you are the new generation you have everything you need, you search Google, you read some article so this is all like you know you are self-creating the things. | Family myths exist  If older women didn’t suffer, younger possibly cannot  Termed as a new generation phenomenon  Perceived as a self-created disease |
| Interviewer: Alright you umm do you think we have enough resources to help this social issue? |  |
| Interviewee: At certain level we don’t have enough resources… | Lack of resources makes matters worse |
| Interviewer: Which levels? |  |
| Interviewee: Again in low educated status | Economic status allows for access to resources |
| Interviewer: Why don’t we have the resources? |  |
| Interviewee: We are unable to diagnose it… we are unable to know that um this is the real problem that we need to worry about and we need to see.. | Lack of importance  Lack of knowledge and specialization |
| Interviewer: So in what cases will we be unable to understand this? |  |
| Interviewee: Sometimes even the mother is unable to understand to you know judge that yes she needs some help and this is not only because she is pregnant and this is the real reason she needs to worry for | Mother is unaware of her own needs  Mother does not give importance to her emotions |
| Interviewer: So how do you think a mother perceives maternal depression? You being a mother, how do you perceive it? |  |
| Interviewee: I have read a lot about it so i feel like I have the symptoms, okay i had those, what my husband say is that whenever i was like initially i was ah, i had PMS, for those who have PMS, those who have premenstrual syndrome you know in, they can be in depression, what my husband initially went, actually he has no sister so he was unable to understand the female psyche, so whenever i was in premenstrual syndrome he told that you have read a lot that is that reason you are considering yourself that you in PMS but eventually like many cycles he realized okay she is in PMS don’t disturb her, this is that your husband’s cooperation is very important and.. | Reading material to create awareness  Awareness session for men who are about to become fathers  Some men lack understanding of female psyche so awareness sessions can help  Men need time to learn the ways of their wives  Husband’s cooperation can makes a huge positive difference |
| Interviewer: How do you think maternal depression affects you as an individual? |  |
| Interviewee: Your feeling of wellbeing, your activities , your personal as well as you family, like what i was doing taking rest again and again, and hyper because i had some sugar cookie like ah that is another case but if you are depressive than you are not able to concentrate on what you are doing…. | Loss of feeling of wellbeing  Lack of motivation to socialize with family  Lack of concentration |
| Interviewer: So what is the medical education frame work of Pakistan and how does it play role in management of depression? |  |
| Interviewee: Yeah different institute, if we talk about over all depression rather than antenatal or post natal, different institutes but usually because of some cultural issues some social issues they don’t seek help they don’t go to the doctor and they think what will the doctor do, they will give you anti-depressants and they just calm and relax your nerves and sleep nothing else, so what they think that the anti-depressants are just to block your mind and make you sleep for longer time…. | Generally, perception is that doctors only prescribe antidepressants  Sleeping pills are often prescribed to relax depressed souls |
| Interviewer: But you think that our medical and nursing curriculum has a component of maternal depression, especially for gynecologists? |  |
| Interviewee: They are not doing much. | Educational framework is inadequate |
| Interviewer: Okay and there is online training or workshops or CMEs or anything as such? |  |
| Interviewee: No, I don’t think so. | No workshops, CMEs or online trainings |
| Interviewer: Have you been part of any? |  |
| Interviewee: No. | Never attended any such thing |
| Interviewer: Do you think that self-efficacy is an associated factor to the rate of diagnosis and management? |  |
| Interviewee: Hmm, definitely. |  |
| Interviewer: How? |  |
| Interviewee: Like you compare your previous efficacy and your present efficacy that i used be doing, what the hell am i doing | MD affects mothers efficacy |
| Interviewer: No…but if you compare it in the form your efficacy towards a patient who is going through depression… |  |
| Interviewee: My efficacy to the patient is ah like being a doctor and being ahhh, like we have read about it so like can consider it and but sometimes i am like nothing is happening and you just will be fine so we just reassure the patient , reassurance is I guess the first step in treating depression. | Reassurance is the first step of treatment  Doctors efficiency affects patients depression |
| Interviewer: So you’ll give that empathy and comfort or compassion will matter? |  |
| Interviewee: Yeah these points do. | Empathy and compassion can help patient |
| Interviewer: So do you think it is your responsibility to diagnose a patient? |  |
| Interviewee: Hmm definitely. | Gynecologist is responsible for her patient in all ways |
| Interviewer: It is your responsibility… so do you think that you are going to have that comfort and confidence required to tell the mother that she is going through this? |  |
| Interviewee: We should make them aware and make them realize that it is a normal thing and that it happen in any women so this is not the unusual thing and hormonal imbalance and changes in pregnancy can lead her to this, this is a normal thing and we guide her that how we’ll help her out of this. | Awareness should be spread  It should be normalized  Hormonal imbalances and pregnancy changes can be a cause  Guidance for help should be preached |
| Interviewer: Do you think that a weak patient doctor relationship because of infrequent flow ups may affect a mother like this? |  |
| Interviewee: Yes |  |
| Interviewer: How |  |
| Interviewee: Like if you visit the doctor frequently or at time than your doctor will not be able to diagnose it and even um like you’ll not be able to be in touch with the doctor, good surrounding and good communication is very necessary, like if you talk to the doctor and you talk about problems, you feel relaxed. | Weak doctor patient relationship reduces rate of diagnosis  Loss of touch with doctor affects mothers  Communication and surrounding is key for healthy mother  Mothers can talk to their doctors  Doctor’s reassurance can have a positive effect |
| Interviewer: So if it diagnosed how can gynecologists help? Only through good words? |  |
| Interviewee: Initially with good words than by you know umm taking the family as well and making them realize that she needs help, initially with a good environment and whatever good maximum good whatever they can give. | Reassurance for mother  Family counseling  Supportive environment |
| Interviewer: Right…so what factors would play a role in the diagnosis and management of the maternal depression….. What would increase or what would reduce level or rate of diagnosis in a hospital setup? |  |
| Interviewee: Like in ah, increase flow of the patients like the saturation of the patients like we are used to of ah not listening to the patient like in OBS, checking 450 or 500, 400 to 500 patients per day so one doctor, so one to one care is not available, so if one to one care is not available we are not able to properly look into their matter like you know patients used to of coming and i am this pain and headache and so and so and we just say okay this normally happens in pregnancy, we are used to of saying that… | Unrealistic flow of patients  400-500 patients per day  Inability to provide one to one care  Patient complaints are ignored  Complaints are believed to be too normal for attention  Mother’s problems are not given precedence unless severe in nature |
| Interviewer: Ahhh… |  |
| Interviewee: So… |  |
| Interviewer: So do you think that the history taking form that you guys normally take… |  |
| Interviewee: The history should be detailed | History is not detailed |
| Interviewer: It is the moment? |  |
| Interviewee: No its not because of shortage of time its not but the history taking and the issues the actual issues of the patients should be listened carefully and so we may help or be able to help them… | Time shortages lead to incomplete history  Issues are not completely heard  Issues are not sorted |
| Interviewer: Are there any proactive monitoring protocol that can help diagnose or manage the patient? |  |
| Interviewee: No | No availability of pro-active tools |
| Interviewer: How do referrals take place? Do they take place? |  |
| Interviewee: Like whenever we have come across any patient with depression or psychosis we are used to referring them to psychiatric ward…. | Severe cases are referred to psychiatric ward |
| Interviewer: Right, is there ever a follow up after the patient has shifted? |  |
| Interviewee: Patient is used to retaining with us, they come the psychiatric, they are used to of coming and checking the patients and they add up some anti-depressants or so | Psychiatrists prescribe anti-depressants whilst patient is admitted |
| Interviewer: So the psychiatrist come to your unit to check? |  |
| Interviewee: Usually, mostly… |  |
| Interviewer: Right so what are the factors that protect the mother from maternal depression? |  |
| Interviewee: Initially the good and healthy environment , planned pregnancy without any complication | Planning, No complications and healthy environment can protect mothers |
| Interviewer: Do you think screening would help, screening of the mother? |  |
| Interviewee: Screening of the mother, it can only help us to predict that okay this woman will have a depression. What I feel like those who are really intelligent or sharp go through with depression a lot | Intelligence can lead to depression  Screening can only help with diagnosis, not management |
| Interviewer: So are there any kind of behaviors, ahh therapies that can help the mother and protect her from… |  |
| Interviewee: Yeah i really want to get head massage from… | Therapies are individualistic |
| Interviewer: So you think this might work individually? |  |
| Interviewee: Yeah individually. |  |
| Interviewer: ….and you think support groups or peer group can help? | Support or peer groups can help |
| Interviewee: Yes, definitely. |  |
| Interviewer: How? |  |
| Interviewee: Like what i have earlier told, like good words, good time if very important, so if we have some support groups, we may be able to you know refer or send the patient or mother to them, they can talk bout he problem thy can give her more time rather than us so she may feel better. | More time to discuss personal issues  People who pay attention to her problems |
| Interviewer: Other comments? |  |
| Interviewee: That’s it |  |
| Interviewer: Alright. Thankyou so much. |  |

| Josephine – Head Nurse *Briefing provided - verbal and written consent obtained* | |
| --- | --- |
| Interviewer: Zachki depression se maaoun ko kia maslihat aaty hain? unko kia health problem face hoty hai? |  |
| Interviewee: Zachki main, matlab keh |  |
| Interviewer: Zachki depression se aik maa ko kia maslay paish aatay hain uski health keh hawalay se? |  |
| Interviewee: Wo mentally disturb hojaty hai wo matlab keh jis tarhan se agar koi usmay hai toh apnay ghar ko tarf se bhi depression main hoty hai kuch apni family ki waja se hoty hai jesay bari family se hai ya choty family se hain kuch khawateen chahty hain keh hum alag rehna chahwngay phir wo chahty hain keh alag rehtay hain toh hamaray sath acha behave ho matlab hamain jo hai har cheez provide honi chaiye | Women become depressed due to many reasons  Women become depressed because of family   Pregnant women feel depressed when misbehaved with   Women become depressed if their needs are not met |
| Interviewer: Sahi |  |
| Interviewee: Han uski waja se jab nahi milty toh uss waja se phir aik ye hai keh kuch larkyan jo hain apnay gharon main matlab keh maikhi ki tarf se achay ussme hoty hain well khanay peenay main pehen ne, orhnay main jab unko apnay susral main nai milta toh uss waja se depression main hojaty hain | Women become more depressed when in-laws provide less love |
| Interviewer: Toh sehat keh kia masail hain |  |
| Interviewee: Sehat se ye hai keh uska jo hai zehni tension jo hai wo zada barhty hai usme jo hai phir unko blood pressure hota hai wo iski tarf se aty hain phir kuch aur internal bataty nai hain toh andar hi andar jo hai wo ghun ki tarhan jo hai kuch  khata rehta hai toh phir beemar hojaty hain toh jab bataty hain toh phir bohat kuch hochuka hota hai toh phir wo apnay health ka khayal nhi rakhteen khaaty nai hain peety nai hain matlab unko apnay ghar walon ko disturb karnay keh lye kuch khana peena nai hai patanai jesay hum unpay bara ahsaan kar rahay hain wo apnay apko andar se kharab kar rahy hain. | Depression enhances tension & blood pressure  Depressed women lack health seeking behavior   Attention seeking behavior is displayed – they stop eating   Depression destroys mother internally |
| Interviewer: Sahi. Aur aisa koi case zachki depression ka jis se aap pe bohat asar para ho? |  |
| Interviewee: Nai aisay toh atay rehtay hain kis kis ka banda sochay aur phir wo karay na. Liken aaj kal yehy chal raha hai keh matlab keh zada jo hai wo khawateen isliye aaty hain keh matlab keh meray susral acha nai hai mujhay ye mila ye nhi milrha wo mujhsay chupa keh rakhty hain main kuch khaa nahi sakty. Aisa hi hai bas, yehy zada tar history hoty hai. Kuch matlab keh shohar jo hain wo acha behave nai krtay unkay sath | Health professionals lack empathy for they see cases frequently  Most common reason for depression is in-laws and husband’s behavior |
| Interviewer: Sahi. Zachki depression keh baray main apko kia samaj hai keh zachki depression kia hota hai? |  |
| Interviewee: Samaj nai aayi meri |  |
| Interviewer: Zachki depression kia hota hai? |  |
| (Long pause by the interviewee) |  |
| Interviewer: Acha toh zachki depression ko aap kis tarhan samajhty hain? Zachki depression kia hota hai? |  |
| Interviewee: Zachki depression toh acha toh nai hai. Usme bachay pe bhi asar hota hai, maa pe bhi hota hai aur phir jo hai mother sick hojaty hai apnay apko matlab dekh nai sakty. Bachay ko bhi zada time nahi de sakty iss waja se | Not a good sign   Effects mother and child   Depressed mother is unable to give time to child |
| Interviewer: Sahi. Toh apko lagta hai zachki depression hai? Hota hai? |  |
| Interviewee: Zachki depression hota hai liken phir wo apnay ghar walon ko bhi itna time nai de sakteen toh usko kuch samaj main nai aata keh main abhi karungy kia, kia kar sakty hun main | Depression is real   Depressed mother is unable to look after family |
| Interviewer: Aur maashray pe iska kitna bojh hota hai? |  |
| Interviewee: Maashrah peh, mashray pe ye bojh hai keh zahir hai k doosray families walay itna support nai kartay toh wo khud hi pareshan ho ker khud hi beemar ho ker bas. Phir aakhri aik din ye hota hai keh wo depression main khatam hi hojaty hai - apnay apko. | Lack of family support leads to depression   Mother’s functionality is affected and so society is affected   Mother is destroyed internally |
| Interviewer: Sahi. Prenatal, postnatal depression kia hota hai? |  |
| Interviewee: Prenatal jo hai wo matlab keh jesay zachki main shuru keh jo din hain unhi main depression wohi jo khanay peenay ka hai khanay peenay ka mujhay khayal nai hai, mujhay aisay hai, mujhay koi parwa nahi karta main pregnant hun har wakt kaam karty rehty hun. Mujhay matlab keh koi poochnay wala nahi hai. Liken sab rakhtay hain wo apnay aap main, wo apnay aapko samajhty hai keh shayad main bohat zada karrhy hun main zada pareshan hun mujhay koi dekhnay wala nai hai uss waja se woh depress rehty hai keh matlab keh meray sath koi help karnay wala nahi hai. | Prenatal refers to starting days of pregnancy   Prenatal: not eating and displaying lack of self care, feelings of being not looked after   Women feel that they are undergoing something no one understands |
| Interviewer: Sahi. Aur zachki depression keh symptoms ki alamaat kia hoty hain? |  |
| Interviewee: Usme patient jo hai woh matlab keh uskay samajh main nahi aaty mainay karna kia hai. Jo kaam karna hota hai wo karty nhi  hain matlab keh kuch patients jo hain wo kaam karna chah rahy hoty hain uska ulta hi karty hain jesay matlab keh koi kaam karnay bethy hain toh wo sochrhy hain mainay shayad ye karna tha toh mainay nhi kia ab meray husband ayengay mujhsay naraaz hongay, meri saas ayegy mujhay bura bhala bolegy | Patients are unable to complete daily functions   Fear of being apprehended by husband and family makes it worse |
| Interviewer: Sahi |  |
| Interviewee: Issi tarhan se jo hai wo, wo pareshaan hi rehty hai matlab ab main iska kia karungy. Kar kuch nhi paaty ab uskay zehen main bohaat saray khayalaat aaty hain keh ab mujhay ab wo kuch bhi nai hai, wo apnay aapsay hi pareshan rehty hai | Patients become agitated of themselves |
| Interviewer: Toh alaamat bas pareshaani hoty hai? |  |
| Interviewee: Nahi aur bhi hongy, liken aik zada tar ye bataty hain keh wo apnay husband ki tarf se pareshaan thy apni saas ki tarf se pareshaan thy. Jesay patient aatay hain koi unsay poochay toh yehy bataty hain | Family plays an important role in triggering depression |
| Interviewer: Ye symptoms hain? |  |
| Interviewee: Haan zada tar yehy hotay hain |  |
| Interviewer: Aur time kitna hoskta hai jab aapko zachki depression hoskta hai? Kabsay kab tak aik maa ko zachki depression hoskta hai? |  |
| Interviewee: Ye toh nai pata liken ye hai keh wo apni poori pregnancy main yehy sochrhy ho | Women remains stressed throughout pregnancy |
| Interviewer: Sahi |  |
| Interviewee: Kuch yehy sochrhy ho keh meray sath aisa hota hai patanahi aisa hi hota rahega main patanahi isliye hi aayi hun patanahi kuch toh bohat kuch sochlety hain liken phir bhi unko kuch samjhanay wala wo kisi se share nahi karteen | Women are unable to share their feelings with others |
| Interviewer: Sahi. Toh aisi konsi maayen hoty hain jinka sabse zada khatra hota hai keh unko zachki depression hoga? |  |
| Interviewee: Jinka blood pressure zada hoga. Uss wakt blood pressure zada hojata hai wo kuch toh bataty hi hain kuch apnay andar hi andar karlety hain phir kabhi behosh hogyi toh kabhi saas le aayi husband le aaye keh iska blood pressure zada hogaya hai toh pata chala keh jee ye toh kaafi depression hai jesay sixth seventh month pregnant hain. Aisay patients atay hain toh issi main.. | Symptoms include high bp   Women faint frequently because of stress   Sometimes they do not know they are pregnant even |
| Interviewer: Sahi. Toh kis tarhan manage hosakta hai zachki depression? Aap kesay uska khayaal kar saktay ho ya usko behtar kar saktay ho? |  |
| Interviewee: Jo uskay paas hai usko ye boltay hain keh jesay abhi isko daantna nahi hai pyar se baat karni hai issay thora samajh ajayegy ahista ahista recover hojayegy ye masla hal hojayega delivery hojayegy sab ye blood pressure bhi normal hojayega aur phir baad main ghar main jo maslay masaail unkay apnay hotay hain toh wo toh chaltau hi rehtay hain. Phir next usme bhi ajaty hain jo aik dafa clamtic hai patient toh ussay phir boltay hain keh dubara jab bacha ho shiru keh dinon main hi ana takay aapka ilaaj hosakay. Issi waja se  ziada ziada se abortion hotay hain depression ki waja se kyunkay ussay samaj nai aaty na keh karna kia hai phir unko batatay  hain khush raha karo ye kia karo husband keh sath khush raha karo unko khush rakhnay ki koshish kia karo khud bhi khush raha karo | Management of Maternal Depression:   Talk to them nicely   Donot scold them   After delivery, bp will come under control   If women suffers from clamatia, she is aksed to visit doctor at start of pregnancy   Most abortions (might be referring to miscarriages) occur when women are stressed and not happy |
| Interviewer: Sahi. Short term main matlab fori tor pe aur long term main, aik lambay arsay tak maa pe kia asar parta hai zachki depression ka? |  |
| (Long pause by the interviewee) |  |
| Interviewer: Agar aik maa ko abhi zachki depression hai toh fori tor pe maa pe kia asar parega? |  |
| Interviewee: Uska abortion hojayega | Maternal depression can result in an abortion (might be refeering to miscarriage) |
| Interviewer: Acha. Uska abortion hojayega |  |
| Interviewee: Abortion hojayega to relief hojayega toh blood pressure bhi theek hojayega wo khud bhi thori der baad mentally theek hojayegy aur phir jab pata chalega ussay ye hogya hai toh hoskta hai woh shock main bhi jaaskty hai | If women miscarries, her BP normalizes, relieving her stress and helping her find mental peace   When she discovers the loss of child, she might go into shock |
| Interviewer: Sahi. Aur lambay arsay main maa pe iss sabka kia asar parega? Aik lambay arsay baad? |  |
| Interviewee: Iska koi jawab nahi hai |  |
| Interviewer: Sahi. Aur bachay pe kia fark parega? |  |
| (Pause by the interviewee) |  |
| Interviewer: Koi asar nai parega bachay pe? |  |
| Interviewee: Bachay pe bhi parega liken jesay hum kehtay hain ye keh maa keh pait se hi bacha bohat sara kuch seekhleta hai karleta  hai liken yehy keh wo kuch bol nhi sakta na. Ye toh natural hai na toh maybe keh wo bacha zada chirchira ho matlab jab bacha bara hoga toh chirchira hoga aur kia | Child learns when in mother’s womb   Maybe the child will be irritable by nature |
| Interviewer: Aur ghar walon pe koi asar parta hai? |  |
| Interviewee: Gharwalon ko kia asar parna bacha jiska hai paal lega bas | Family is not affected |
| Interviewer: Sahi. Aur shohar pe ya baaki bachon pe ya jo uska khayal rakh rahay houn unko koi fark parega? |  |
| Interviewee: Bas wo next pregnancy pe bolengay khayal rakhna pichlay wakt main ye hua tha wesa hua tha tum apna khayal nai rakhty. Wo balkay usko aur zada depression dedengay | Husband and family will depresss her more by blaming her for not taking care of herself |
| Interviewer: Sahi. Kia Pakistan main kisi tarhan ka koi hal nikala jata hai, depression manage kia jata hai? |  |
| Interviewee: Mushkil hai wesay toh bohat saray wo hain women  keh hain  phir bhi koi hal jo hai wo itna khaas nai hai | Many ways to manage it but difficult to do so Pakistan |
| Interviewer: Sahi. Toh hospitals koi tool istimaal kartay hain dekhnay keh lye keh kisi maa ko depression ho? |  |
| Interviewee: Psychiatric main kartay hongay liken yahan toh  nhi dekha | No tools used by hospitals in Gynae ward |
| Interviewer: Kabhi maa aaty hai toh aap log uss se sawalat kartay ho jis se pata chalay usaay zachki depression hai ya nai hai? |  |
| Interviewee: Nai filhaal toh wesay hi agar koi patient aayi toh uss se poochtay hain blood pressure kia hai toh wo bataty hai wo pehlay bachay main bhi hua tha doosray main bhi phir kuch shy feel karty hain apni ghar ki baaten batanay keh lye toh phir  toh jo koi uskay sath hota hai wo bata deta hai isko ye hua tha ye hua tha iski saas ne isko aisa kia hai uskay husband ne aisa kia matlab keh maa ki tarf se pareshaan hai | No questions are asked about mother’s mental status   Sometimes mothers feel shy and sometimes they tell   Family members shed light on the stressors in some instances |
| Interviewer: Apnay koi sawalat ya kuch kisi se poochwn houn zachki depression keh hawalay se? |  |
| Interviewee: Ham bhi yehy poochtay hain insay keh matlab keh kia hua tha ya ye kyun hogaya aap khayal nai rakhtay toh wo phir kuch toh bata dety hain | Only ask reasons if woman claims to have suffered from symptoms in earlier pregnancies |
| Interviewer: Sahi. Aur apko lagta hai koi treatment koi tareeqa hota hai jis se zachki depression sahi hosakay? |  |
| Interviewee: Zachki toh nai wesay wo jo ward 20 main ho hai therapy kartay hain usmay hai doctor, kia naam hai yaad naheen | No treatment in our ward |
| Interviewer: Psychiatry department main? |  |
| Interviewee: Haan usmay jo hai wo bhejtay hain wo kartay hain | Treatment available only in psychiatry ward |
| Interviewer: Aur maayen jinko hota hai wo jaaty hain doctor keh paas agar unko pata bhi ho unko zachki depression hai? |  |
| Interviewee: Haan aik baar jab bhejdya jaye toh chaly jaty hain uskay baad jo hai phir jayengay phir ghar se fursat nhi hai janay keh lye keh time hi nhi mila time nikal gya tha iss tarhan se. | Women only visit the counselor when referred to due to lack of family support   No followups are done because of lack of time |
| Interviewer: Sahi. Aur kia wajoohat hain? Wo sirf time ki waja se nahi jaateen ya aur bhi koi wajoohat hain jiski waja se nhi jaateen? |  |
| Interviewee: Time ki waja se bhi nai jaty kuch ghar ki waja se ghar walay kehtay hain chordo kia kuch bhi nai hai tum aisay hi time waste karty ho idher udher jaty ho | Sometimes family doesnot support followups |
| Interviewer: Sahi. Kia ismay apkay kuch religion ka ya education ka koi role hota hai? |  |
| Interviewee: Nahi | Religion and education play no role |
| Interviewer: Aik maa jisay zachki depression wo apnay baray main khudsay kia sochegy? |  |
| (Pause by interviewee) |  |
| Interviewee: Jab hoga tab sochegy liken jab farig hogy kuch nai sochegy har cheez bhool jayegy keh kuch bhi nhi tha bas | Mother will remain stressed only when pregnant |
| Interviewer: Sahi. Apko lagta hai keh Pakistan ka jo medical framework hai, medical curriculum jo aap parhtay ho usmay zachki depression ka kuchh bataya jata hai ye management ka kuch bataya jata hai? |  |
| Interviewee: Itnay sawal jawab kiye sab main yehy batatay hain keh aisa karna hai aisa krna hai |  |
| Interviewer: Toh apko zachki depression keh baray main sikhaya jatay hai apkay 4 saalon main. Kia sikhaya jata hai? |  |
| Interviewee: Bas yehy keh patient keh sath politely baat karni hai usko achi tarhan se samjhana hai uss se pyar se baat karni hai ussay jo hai wo apni purani baaten khatam karke jo bhi uskay sath hui | Medical framework only emphasizes on empathetic listening, talking politely and with ove |
| Interviewer: Job pe training hoty hai inn cheezon ki? |  |
| Interviewee: Job pe hoty hai matlab keh physchiatric pe hoty hai. Kuch log kartay hain kuch nahi kartay. | The training is not mandatory |
| Interviewer: Kia apko lagta hai keh ye apko responsibility hai keh agar aik maa ko zachki depression hai toh aap uski madad kro? |  |
| Interviewee: Ye toh sab main keh har shakhs ki karni chaiye liken ye hai keh in me jo hai zada hota hai kyunkay inko phsycho therapy bohat zaroori hoty hai madad keh lye | We should look after all patients   Depressed mothers have a high need for psychotherapy |
| Interviewer: Sahi. Aur apko lagta hai keh aap baray confidently bohat aitamaad keh sath uss se ye baat kar sakty hain? |  |
| Interviewee: Karsakty hun. | She can confidently talk to patients about it |
| Interviewer: Sahi. Aksar jo hai patient follow up nahi karta toh apko lagta hai uss se asar parta hai uskay dimagi tawazun pe? |  |
| Interviewee: Hmm kyunkay ussay jo treatment di hoty hai patani usnay li bhi hai ya nhi aur li hai toh matlab time se li hai ya galat hi khaty rahy hai ya to phir khaai hi nhi hai khareedi hi nhi hai. Kuch patients aisa bhi kartay hain keh lia hi nai hai keh hamaray pas financial problems hain keh hum ye nai kar saktay afford nai kar saktay thay. | Women donot followup and it affects their health negatively   Women may not buy meds, take them on time or eat the wrong pills   Some patients don’t buy any meds because of financial constraints |
| Interviewer: Aur agar kisi patient ko hum bataden keh bhui diagnose hogaya hai keh zachki depression hai toh apko lagta hai gynaecologist kuch kar sakty hai? |  |
| Interviewee: Gynochologist toh sure hi kar sakty hai keh ye ye problem hai apnay ye karna hai aap ye karo aap aisa kia karo ye department hai aapnay udher ana hai wahaan pe ye matlab iss cheez ki therapy hoty hai wahan medicine milty hai ye ye baten bataty hain aur wo yehy kar saktay hain. Zahir hai itnay log hotay hain toh kisi keh pas toh nhi jaa saktay har aik ko toh nhi kar saktay | Gynecologist can definitely help   She can create awareness   She can provide referral for therapy and medicine   Due to lack of time, she cannot help all patients |
| Interviewer: Sahi. Aur aapko lagta hai general practioner first line of support ban saktay hain matlab pehla banda jis se baat karty hai aik maa, wo aik general practioner hota hai - gp hota hai. Apko lagta hai unhay koi knowledge hony chaiye zachki depression keh baray main? |  |
| Interviewee: Han knowledge hogy toh woh sab kuch batayengay na unko keh ye karna hai ye karna hai | General paractioners should be well versed |
| Interviewer: Aik hospital main aisi kia wajoohat hain jiski waja se zachki depression jo hai wo diagnose bhi na ho aur patta bhi na chalay aur maa aisay hi chali jaye? |  |
| Interviewee: Nahi aisa nahi hota | It doesnot go undiagnosed |
| Interviewer: Kabhi aisa hota hai keh bohat saray log kharay hogaye aa ker aur jaldi jaldi history leli aur patient ko agay kardya? |  |
| Interviewee: Nahi | Did not think that time is a contributing factor |
| Interviewer: Kia aisa hai keh apka jo assessment hota hai jab maa aaty hai uss se sawalat hotay hain, usmay kaheen zachki depression keh hawalay se koi sawalat hotay hain? |  |
| Interviewee: Hotay toh nhi hain liken ye hai keh kuch patient hain jo khud hi matlab ke keh dety hain ke jesay keh aik dafa Sheereen doctor unko kuch nai bataya toh unho ne poocha apnay goli nai khayi ye nai kia toh wo phir apna thora bohat boldety hai kia masla tha acha chalo mjhy batao main tumhary help karty hun | Questions not part of assessment   Patients tell themselves  Doctors probe if patients say something and help accordingly |
| Interviewer: Aur agar aap refer kartay ho patient ko toh aisa hota hai kabhi keh zachki depression hai toh refer kardya patient ko psychiatry ward ya kuch? |  |
| Interviewee: Haan ye toh kartay hain | Patients are referred to psychiatry ward if doctors suspect anything |
| Interviewer: Sahi. Aisi kia cheezen hain jis se aap aik maa ko madad kar saktay ho keh wo zachki depression ka shikaar na banay? |  |
| Interviewee: Usko ye kehtay hain keh dubara jab aogy, jab dubara pregnant hogy toh ab shuru se ana phir apko treatment hogy apko dekha jayega sab matlab keh apka follow up hoga aapko medicine matlab keh ye ye karna agar zada hai toh phir unko psychiatric ward kehtay hain keh unko wahan bhi dikha ya jaye aik dafa wahan follow up lelena wahan kia keh dahay hain | If needed, they are sent for followup and medicines to psychiatry department |
| Interviewer: Sahi. Aur apko lagta hai keh koi khaas qism ki therapy hai jis se hum ye rok saktay hain ya madad kar saktay hain ya khatam kar saktay hain? |  |
| Interviewee: Hmm hai | There are therapies to manage and treat it |
| Interviewer: Kia therapy hai? |  |
| Interviewee: Psychiatric ka bataya na pyschiatrist bethay hotay hain na unki help kar sakay hain | Psychiatrists help them |
| Interviewer: Sahi. Koi aur baat jo aap batana chahen? |  |
| Interviewee: Nahi bas khatam hogaya? |  |
| Interviewer: Jee, bohat bohat shukrya apka |  |

| Nasreen – Nurse *Briefing provided - verbal and written consent obtained* | |
| --- | --- |
| Interviewer: Apnay kitnay saalon tak gyny main kaam kia hai? |  |
|  |  |
| Interviewee: 7 years |  |
|  |  |
| Interviewer: Theek hai jee. Apki kia samaj hai un health problems ki jo keh maaon ko paish atay hain jab unko zachki depression hota hai? |  |
|  |  |
| Interviewee: Mostly jo hamaray paas aty hain wo ghar keh problems hotay hain husband compromise nahi kartay aur convence problems hotay hain aur doosra second ye keh unki diet imbalanced hoty hai diet achi nhi milty jiski waja se mothers yahan per aaty hain aur mostly jo patients hoty hain unki yehy khwahish hoty hai keh wo apna pregnancy ka time period jo hai wo yaheen pe guzaar keh jayen unhay ye hota hai keh yahan pe doctors aur nurses har cheez available hoty hai. Toh isliye abhi bhi madam upar hamari yehy discussion chal rhy thy keh kuch patients ko bol rahay thay aap weekly aa ker apna check up karwao toh wo keh rhy theen hum dur se atay hain hamaray problems hotay hain hum yaheen pee reh kar karwayengay | Factors such as non-compromising husbands, mobility issues and imbalanced diet lead to maternal depression  Most females want to stay in the hospital amongst doctors and nurses throughout pregnancy  Most pregnant women like to stay at the hospital for long time periods  Females like to stay in hospitals for long time periods since all their needs are taken care of  Mothers donot visit the hospital regularly |
|  |  |
| Interviewer: Liken aik maa ko kia problem ata hai health keh hawalay se jab ussay zachki depression hota hai? |  |
|  |  |
| Interviewee: Yehy hota hai keh ghar pe unkay khanay peenay ka jo hai unkay husband jinki income zada nai hoty job achi nai hoty wisaayal achay nai hotay ya kabhi aisa hota hai keh ghar main agar wesay ussay husband ki tarf se koi problem nai ho toh inlaws ki tarf se masaayal hojatay hain ya phir aisa hota hai keh patients keh khud hb waghera ya koi internal usko apna koi problem nhi ho. Aksar patients aisay atay hain jinko stomach problems keh sath aty hain, cardiac patients bhi aty hain toh jinka intake acha nahi hota, aur kuch toh aisi hoty hain jinko continue during pregnancy main vomiting hoty hai toh ye sabsay unke lye major problem hota hai keh wo kuch intake le nahy sakty hain jiski waja se wo four five six month tak bhi issi problem main rehty hain | Women are not looked after because of finnacial issues  Inlaws make life difficult for pregnant women  Sometimes women suffer internally for 4 – 5 months with cardiac issues, continuous vomiting or stomach issues, leading to stress |
|  |  |
| Interviewer: Kia ye cheez unhay zachki depression ki waja se horhy hoty hai? |  |
|  |  |
| Interviewee: Nahi, zachki depression ki waja se nhi ye hormonal changes ki waja se horhy hoty hain | Hormonal changes act as stressors causing internal issues |
|  |  |
| Interviewer: Sahi. Kia aisi koi beemari hai jo aik maa ko hoty hai zachki depression ki waja se? |  |
|  |  |
| Interviewee: Nai aisa koi masla nhi hai | No issues occur due to maternal depression |
|  |  |
| Interviewer: Acha, aisa koi case ya aisi koi khatoon ya koi maa jiski waja se apkay upar koi asar huwa ho? |  |
|  |  |
| Interviewee: No |  |
|  |  |
| Interviewer: Sahi. Umm, jab main kehty hun zachki depression toh uss se apko kia samajh ata hai main kia baat kar rahy hun? |  |
|  |  |
| Interviewee: Yaanay keh jo pregnant aurten hoty hain aap unka bol re ho na jesay unko batao keh kia problem hoskte hain pregnancy keh doraan- aap ye kehna chah rahay ho na ya aap ye kehna chah rahay ho keh jesay pehlay se.. | Issues with pregnant women equates to maternal depression |
|  |  |
| Interviewer: Main apsay pooch rahy hun keh zachki depression ka lafz istimaal karty hun.. |  |
|  |  |
| Interviewee: Jee |  |
|  |  |
| Interviewer: Toh apko kia samajh ata hai? |  |
|  |  |
| Interviewee: Yehy keh during pregnancy main kia problem hoskty hai | Understands Maternal Depression as all issues during pregnancy |
|  |  |
| Interviewer:Sahi. Kis hawalay se kia problem hoskty hai? |  |
|  |  |
| Interviewee: Pregnancy keh hawalay se aap keh rahay ho |  |
|  |  |
| Interviewer: Acha, zachki depression bolty hun toh kia samajh ata hai? |  |
|  |  |
| Interviewee: Depression se tension keh kabhi maa usko koi na koi bhi during pregnancy main jesay pregnancy ki waja se koi masla hai toh woi uski waja se depression hai | Depression is tension  Tension leads to depression |
|  |  |
| Interviewer: Sahi. Apko lagta hai zachki depression hamaray maashray main paya jata hai? |  |
|  |  |
| Interviewee: Jee | MD is present in our society |
|  |  |
| Interviewer: Aur uski waja se hamari society pe kia effect parta hai? Society pe kitna bojh hai uss cheez ka? |  |
|  |  |
| Interviewee: Ye toh explain kariyega please |  |
|  |  |
| Interviewer: Acha, society jo hamari hai uspe kia bojh hota hai jab aik maa zachki depression se guzarty hai. Kia hamaray maashray pe koi bojh parta hai iss cheez ka? |  |
|  |  |
| Interviewee: Wo toh mothers pe hi hai na uski uspe hai wo apni life pe apni pe hota hai otherwise baaki uskay lye apni family keh lye hota hai uska husband, specially husband keh lye hota hai | Mother’s sickness affects mother and family, specially husband |
|  |  |
| Interviewer: Theek hai |  |
|  |  |
| Interviewee: Toh aur uskay jo apnay bachay hotay hain unke lye hota hai keh wo apnay bachon ko attend nhi kr paaty. Otherwise society pe toh itna nahi hota agar aap dekhen | MD affects children  Mother is unable to look after children  It doesnot affect society overall |
|  |  |
| Interviewer: Sahi. Jab main prenatal aur postnatal depression ki baat karty hun toh apko kia samajh ata hai? |  |
|  |  |
| Interviewee: Prenatal ka ye hai keh during pregnancy aur postnatal hai jo hai wo after | Prenatal is during pregnancy  Postnatal is after pregnancy |
|  |  |
| Interviewer: Sahi. Aur depression keh baray kabhi kuch suna kuch parha? |  |
|  |  |
| Interviewee: Depression keh baray main yehy hai keh insaan ki life disturbs hojaty hai bilkul | Depression disturbs ones life |
|  |  |
| Interviewer: Koi alaamat jo apko zachki depression keh baray main pata houn? |  |
|  |  |
| Interviewee: Urmmm, blood pressure hojata hai unko, blood pressures ki hojaty hain aur wesay hi hai keh unkay bolnay ke rawaiye main thori si changes ajaty hain yanay keh disturbance ajata hai aur bas yehy hai keh | BP, disturbed behavior are signs of MD |
|  |  |
| (Short pause) |  |
|  |  |
| Interviewer: Aur kia time hota hai jab zachki depression hota hai aik maa ko? |  |
|  |  |
| Interviewee: Timing toh koi exact nai hota hai liken pregnancy keh doran jo hota hai jesay jesay pregnancy barhty hai month apkay zada hotay hain toh yanay keh during, starting main ye hota hai keh starting main bhi hota hai keh aksar patients jab meeting karty hain usme bhi ghar walay boltay hain khao peeyo toh banday main chirchira pan sa ajata hai aur last main ye hota hai keh last main jo hoty hain jesay seventh eighth aur nineth usmay ye hota hai keh period zada hojatay hain toh usmay bhi insaan apnay apse hojata hai keh pregnancy main bohat mushkil hota hai apnay apko karna toh usmay bhi disturbance hojaty hai | No exact timeline  Starting months of pregnancy, mothers become irritable  MD can occur throughout pregnancy  Mothers donot take care of diet |
|  |  |
| Interviewer: Seventh eight month main periods zada hojatay hain? |  |
|  |  |
| Interviewee: Periods nai zada hotay. Pregnancy ki agar aap baat kar rahay ho toh period pregnancy ka |  |
|  |  |
| Interviewer: Toh jesay jesay seventh eight month lagta hai aap keh rahay ho |  |
|  |  |
| Interviewee: Jee |  |
|  |  |
| Interviewer: Usmay maa zada feel karty hai? |  |
|  |  |
| Interviewee: Jee | Mothers feels more in last months of pregnancy |
|  |  |
| Interviewer: Aur bacha denay ke baad? |  |
|  |  |
| Interviewee: Bacha honay keh baad toh relax hojaty hain | After the baby is born, mother realxes |
|  |  |
| Interviewer: Relax hojaty hain, theek hai |  |
|  |  |
| (Short pause) |  |
|  |  |
| Interviewer: Maaon keh andar sabse zada khatra kissay hota hai zachki depression honay ka? |  |
|  |  |
| Interviewee: Maa bachay ko | Mother and child are vulnerable |
|  |  |
| Interviewer: Liken kesi maa ko khatra hota hai sabsay zada honay ka? |  |
|  |  |
| Interviewee: Jesay ko eclamtic patient hojaty hain blood pressure keh zada honay ki waja se wo zada hojaty hain | Mothers with clamatia and bp are at high risk |
|  |  |
| Interviewer: Sahi. Aur kesi maaon ko hoskta hai? |  |
|  |  |
| (Long pause by interviewee) |  |
|  |  |
| Interviewee: Pata nahi |  |
|  |  |
| Interviewer: Aur agar kisi ko hai toh aap manage kesay karogay zachki depression? |  |
|  |  |
| Interviewee: Uskay saath baat waat karke ussay relax karengay ussay bolengay keh tabyat kharab na rakho ye apkay lye bhi aur bachay keh lye bhi faidamand hai theek hai, aur doosra ye keh unko phir tablets waghera detay hain thora sa relax hojayen patient toh ye hai according to doctor advice | Talking helps  Telling mothers that taking care of their health will affect their child’s health positively  By giving medications according to doc’s advice |
|  |  |
| Interviewer: Acha, aur short term main matlab fori tor pe aur long term lambay arsay tak kia asraat hotay hain zachki depression keh? Maa pe kia asraat hotay hain? |  |
|  |  |
| Interviewee: Depression keh? Haan ye hai keh aap dekhogay jesay wo depression ka shikaari hai hyper hojaty hain diabetic hojaty hain kuch ki eyes pe honay lag jata hai theek hai aur iskay elawa jo aik routine main depression ka shikaar hoty hain uski habital life hojaty hai keh wo aik routine main keh usko jo cheez achi lagty hai wo phir jo hai zara si kisi cheez se pe ho to wo tension main hyper hojaty hain | Depression makes mothers hyper  They become diabetic  Their eyes are affected  Daily routine is affected  She remains tensed |
|  |  |
| Interviewer: Sahi. Aik bachay pe kia fark parta hai? |  |
|  |  |
| Interviewee: Bachay pe nai parta | MD doesnot affect child |
|  |  |
| Interviewer: Koi fark nahi parta? Sahi. Gharwalon pe kia fark parta hai? |  |
|  |  |
| Interviewee: Life disturb hoty hai | Family’s life is disturbed |
|  |  |
| Interviewer: Kesay disturb hoty hai? |  |
|  |  |
| Interviewee: Bachay jo hai wo tension main rehtay hain ghar ka mahol bilkul setup nhi ho pata | Children stay tensed  House environment is not settled |
|  |  |
| Interviewer: Aur tamaam jo care givers hotay hain jo maa ka khayal rakh rahay hotay hain kia unpay koi asar hota hai? |  |
|  |  |
| Interviewee: Nhi | Cargivers are not affected |
|  |  |
| Interviewer: Unpay asar nhi hota? Sahi. Aur unkay jo husband hotay hain kia unpay koi asar hota hai? |  |
|  |  |
| Interviewee: Jee unpar toh hota hai. Baaki family iss cheez pe itna matter bhi nahi lety nahi joty yanay keh wo iss cheez ko itna wo hi nahi karty liken husband aur bachay jo hain wo suffer kartay hain | Husband suffers  Children suffer  Family doesnot take it into consideration much |
|  |  |
| Interviewer: Sahi. |  |
|  |  |
| Interviewee: kyunkay wo unko spend karna hota hai mother keh sath toh jo bhi maa jesa bhi uska rawaya hota hai bachon keh sath father keh sath toh wo face kartay hain iss cheez ko | Husband and children spend most time with mother  Husband and children suffer the most |
|  |  |
| Interviewer: Sahi. Pakistan main kesay zachki depression ko manage kia jata hai? |  |
|  |  |
| (Short pause by interviewee) |  |
|  |  |
| Interviewer: Ya nahi kia jata? |  |
|  |  |
| Interviewee: Kia jata hai |  |
|  |  |
| Interviewer: Kesay kia jata hai? |  |
|  |  |
| Interviewee: Jesay yahan pe sgpo walay apnay dekha hoga iss tareekay keh bohat saray institute madam hamari Dr. Halima jo hain wo itna zada zachki pe karty hain wo programs karatay hain mothers ko batatay hain aur aksar aisa hota hai jesay unki help bhi ki jaty hai mothers ke sath keh hum apko ye cheez provide karengay apko apna khayal rakhna hai iss tarhan hota hai | During pregnancy: Organizations work for mothers, Programs for women are arranged, Mothers are helped by provision of items needed during pregnancy |
|  |  |
| Interviewer: Depression ki baat hoty hai program main? |  |
|  |  |
| Interviewee: Nahi depression ki nhi yanay unko aik tareekay se bataya jata hai |  |
|  |  |
| Interviewer: Zachki ki baat hoty hai keh aik maa ko kia hota hai? |  |
|  |  |
| Interviewee: Jee jee | No specific discussion on depression takes place in programs |
|  |  |
| Interviewer: Apko lagta hai keh hospitals koi tool istimaal kartay hain zachki depression diagnose karnay keh lye? |  |
|  |  |
| Interviewee: Tool means keh? |  |
|  |  |
| Interviewer: Koi jesay apka hota hai na history taking kartay ho |  |
|  |  |
| Interviewee: Jee jee jee |  |
|  |  |
| Interviewer: Iss tarhan ki koi cheez? |  |
|  |  |
| Interviewee: Jee jesay doctors hamari hoty hain wo sara matlab keh dekhty hain jo patient depression main, jo patient depression main hota hai toh already uska blood pressure bp waghera pe disturbance hoty hai ya ye hota hai keh neend na poori honay ki waja se bhi patient toh wo usmay wo karleti hain | If patient has high bp, she is disturbed  Doctor checks for MD when patient is disturbed |
|  |  |
| Interviewer: Jab aik maa pehly baar hospital aaty hai unsay sawal kartay hain toh kia zachki depression keh baray main koi sawalat poochtay hain? |  |
|  |  |
| Interviewee: Aisa toh nahi hota | No questions are asked as part of initial screening |
|  |  |
| Interviewer: Apnay kabhi kisi se zachki depression keh hawalay se sawalat kiye houn ya koi cheez? Ya kisi maa se koi sawalat karnay keh lye koi tool istimaal kia ho? |  |
|  |  |
| Interviewee: Bohat sari patient aaty hain aur hoskta hai on the spot jesa lagta hai phir hum manage kartay hain unkay sath | There are a lot of patients  If on spot we feel there is some issue, we manage |
|  |  |
| Interviewer: Kesay manage kartay hain? |  |
|  |  |
| Interviewee: Matlab unko bataty hain keh aap tension nhi lein aur aksar aisa hota hai yahan pe toh zada iss tarhan hota keh unko pata nahi hota toh wo uss chakar main bohat zada disturbance main atay hain keh hamari help ki jaay humain iss cheez ka nhi pata toh phir uss chakkar ham unki help kardetay hain batatay hain aap yahan jayen | We tell them not to stress  Lack of information about what needs to be done during pregnancy stresses mothers  We guide them |
|  |  |
| Interviewer: Sahi. Aapko lagta hai keh zachki depression ko treat kia jaskta hai ya usko manage kia jaa sakta hai? |  |
|  |  |
| Interviewee: Jee | MD can be managed |
|  |  |
| Interviewer: Kesay? Aapnay koi tareekay dekhay hon jis se usko manage kia gya ho ya treat kia gya ho? |  |
|  |  |
| Interviewee: Yehy jesay main aapko pehlay bata chuki hun keh doctors keh patient jab ata blood pressure waghera unka check kia jata hai phir uskay baad unko bataya jata hai keh apka blood pressure zada hai ya bohat zehni tension hai toh aap apnay apko relax kia karo toh issi tarhan se manage kia jata hai | Check BP  High blood pressure equates to high levels of tension  Patient is counseled to relax |
|  |  |
| Interviewer: Kia koi aisi treatement hai – agar hai tou - hamaray maashray main jo aapko lagta hai keh maayen ya ghar walay maaon ko le ker jayengay iss cheez keh lye? |  |
|  |  |
| Interviewee: Jee bilkul jana chaiye le ker |  |
|  |  |
| Interviewer: kia lekai jatai hai? |  |
|  |  |
| Interviewee: Jatay bhi hongay kuch nahi bhi le ke jaatay hongay jatay bhi hain | Some families may take patients for treatment |
|  |  |
| Interviewer: Sahi. Aisi kia cheezen hain jo hum kar saktay hain jis se aik maa depress na ho? |  |
|  |  |
| Interviewee: Mahol usko ghar ka khushgawar milay achay log milen yanay keh uski khwahishen har cheez jo uskay chaiye wo uskay bolnay se pehlay ussay miljayen. Theek hai. Care chaiye ussay jesay keh toh wo meray khayal main nhi depression hoga | Happy home environment  Fulfilling all her wishes  Excessive care |
|  |  |
| Interviewer: Aur agar kisi maa ko zachki depression hai toh aisi kia cheezen hain keh log ya maayen khud doctor keh paas nahi jaateen? |  |
|  |  |
| Interviewee: Matlab keh facilities nahi hoteen unkay pas ya koi lejanay wala nhi hoga jiski waja se wo wahan tak nahi jaa pateen | Lack of facilities  Women are dependent on others to visit hospital |
|  |  |
| Interviewer: Sahi |  |
|  |  |
| Interviewee: Kyunkay aisa hota hai keh unkay pas itni awareness nahi hoty keh wo apnay apko doctor keh paas ya matlab keh wo ismay education ka bhi hota hai keh jo educated nhi hain unko nhi pata hoga keh kahan jana hai phir unkay lye unhay chaiye hoty hai koi | Not enough awareness to be able to visit doctor  Not educated enough to know their way to doctor  Need someone to guide them  Need someone to take them to doctor |
|  |  |
| Interviewer: Sahi. Apko lagta hai keh religion ka iss se lena dena hai keh maayen jayengy ya nahi jayengy? |  |
|  |  |
| Interviewee: Nahi religion ka nhi | Religion plays no role |
|  |  |
| Interviewer: Aur apko lagta hai keh hamaray paas itnay log hain iss treatment keh lye agar koi maa jana chahay? Kia hamaray paas itnay professionals hain? |  |
|  |  |
| (Short pause interviewee) |  |
|  |  |
| Interviewer: Kia hamaray paas itnay doctors hain parhay likhay? |  |
|  |  |
| Interviewee: Jee aap jesay JPMC main itnay hotay hain itnay doctors hain aur itnay log atay hain sab treat hotay hain yahan pe | We have enough professionals |
|  |  |
| Interviewer: Liken kia depression keh lye treat hotay hain log? |  |
|  |  |
| Interviewee: Depression keh lye, uskay lye toh phir alag unit hoga na unka | Depression unit will be different |
|  |  |
| Interviewer: Alag unit hotay hain theek hai, jis maa ko depression hai, wo apnay apko kesay samjhayegy keh depression hai? Kia mehsoos hoga ussay depression keh hawalay se? |  |
|  |  |
| Interviewee: Uss wakt toh mera nahi khayal keh mothers ko itna sense ho keh bas uss wakt ye hota hai keh wo depression main hoty hai toh wo bas jo unka dil hota hai wo karty hain | They do what they feel like doing  They are not in their senses |
|  |  |
| Interviewer: Sahi. Jo hamara medical curriculum hai jismay hum seekhtay hain jo chaar saal ki aap log ki parhai hoty hai, kia aapko lagta hai keh uss se koi lena dena hai keh apko kitna zachki depression keh baray main ata hai ya nahi ata? |  |
|  |  |
| Interviewee: Jee hai, usmay bataty hain depressions ka | Medical Curriculum teaches us about depression |
|  |  |
| Interviewer: Usmay batatay hain.. |  |
|  |  |
| Interviewee: Zachki ka  fourth year main ata hai hamara general nursing ka third year tak hota hai, fourth year main aa ker phir gyny ka hota hai toh bataty hain | Gynae details are taught in Year 4 |
|  |  |
| Interviewer: Toh depression keh baray main alag se bataty hain? |  |
|  |  |
| Interviewee: Depression keh baray main toh phir wo during syllabus hota hai phir wesay patients dekhtay hain phir discussion hota hai phir jesay | Taught about depression during classes  Discussions take place when we see patients |
|  |  |
| Interviewer: Sahi. Toh apko lagta hai keh job pe koi training hoty hai depression keh hawalay se ya koi workshop ho iss tarhan se? |  |
|  |  |
| Interviewee: Hoty reht hain workshops toh | Workshops often take place |
|  |  |
| Interviewer: Depression keh upar? |  |
|  |  |
| Interviewee: Depressions pe toh mainay attend nhi ki liken workshop hoty rehty hain | Not attended any workshop on just depression |
|  |  |
| Interviewer: Sahi. Aur kabhi CME suna hai kia hota hai? |  |
|  |  |
| Interviewee: Nahi iska toh nhi | Don’t knoe what CME is |
|  |  |
| Interviewer: Sahi. Kia apko lagta hai keh apki apni bhi koi responsibility hai patient ki tarf? |  |
|  |  |
| Interviewee: Jee bilkul | I am responsible for patient |
|  |  |
| Interviewer: Apko lagta hai keh aik patient ko kitni jaldi diagnose kia jaaye ya kitni jaldi manage kia jaaye wo apki apni responsibility hai? |  |
|  |  |
| Interviewee: Hamari bhi hoty hai liken humain allowed nahi hota keh hum diagnose kar saken toh uskay lye phir hum doctors se help letay hain | Responsible for managing patient  Not allowed to diagnose without doctor |
|  |  |
| Interviewer: Sahi. Toh agar aapko lagta hai k koi maa zachki depression ka shikaar hai.. |  |
|  |  |
| Interviewee: Jee |  |
|  |  |
| Interviewer: Toh apko lagta hai keh apki zimedaari hai keh aap doctor ko batao? |  |
|  |  |
| Interviewee: Jee bilkul. Jee | Responsible to look after depressed mother |
|  |  |
| Interviewer: Aur apko lagta hai hamdardi ka iss se koi lena dena hai? |  |
|  |  |
| Interviewee: Jee bilkul. Hamdardi bhi aur dekhen keh tawajo ya usko kisi cheez ki need hai toh wo hamain poori karni hai hamari nursing main bhi yehy hai keh hamain patient zahir si baat hai keh patient takleef main hai toh hum log uski problem solve karen bas yehy hai | Empathy and compassion is important  Meeting patient’s needs is important  It is our responsibility  Patient is in pain  We need to solve patient’ issues |
|  |  |
| Interviewer: Aur apko lagta hai keh kitna aap patient ko aitemaady do ya sakoon do ya uss se fark parta hai? |  |
|  |  |
| Interviewee: Jee bilkul | Confidence and comfort can help patient |
|  |  |
| Interviewer: Sahi. Toh aksar ye hota hai keh aapko bhi mehsoos hota hai keh patients jo hain wo sahi se follow ups nahi krtay doctor keh sath? |  |
|  |  |
| Interviewee: Jee, nahi kartay | Patients don’t follow up |
|  |  |
| Interviewer: Nahi krtay, acha toh agar hojaye kisi ka farz karen zachki depression hai pata chal jaye toh kia gynochologists unko help kar sakty hain? |  |
|  |  |
| Interviewee: Wo bhi karty hain liken agar pehlay se hi agar koi problem chal raha ho unka toh doosri ward se wo karty hain jo inke lye hoty hain | Gynaecologist help if patient is diagnosed  Patient is referred to psychiatry ward |
|  |  |
| Interviewer: Toh agar pehlay se masla chal raha ho pehlay se masla na chal raha ho toh mujhay ye bataye keh doctor patient ka jo relationship hota hai doctor kitni aik patient se taawun kar rahy hai kia uss se fark parta hai? |  |
|  |  |
| Interviewee: Jee bilkul fark parta hai uss se bhi | Doctor patient relationship is important |
|  |  |
| Interviewer: Sahi. Kia apko lagta hai general practioner ko aik aisa insaan hona chaiye jis se patient ye saray zachki depression keh sawalat poochay jaayen unsay? |  |
|  |  |
| Interviewee: Jee poochna chaiye jee jee | GP should ask questions |
|  |  |
| Interviewer: Achaa aur aik gynaecologist kia kar sakty hain in cheezon keh lye? |  |
|  |  |
| Interviewee: Depression keh lye? Wo yehy patient ki history waghera lengy poochengy agar toh unko lagta hai keh ye during pregnancy hai to ye ussay solve out karengay otherwise wo jesay keh mainay apko pehlay bataya agar usko pehlay se depression hai toh uskay lye phir wo boltay hain keh aap alag se aap treat karen | Gynae can take history  If MD is suspected, referral can be done |
|  |  |
| Interviewer: Acha, kia hamaray jo forms hotay hain jo patient pehly baar ata hai.. |  |
|  |  |
| Interviewee: Jee |  |
|  |  |
| Interviewer: Jis se aap history letay ho usmay koi aisa jaga hoty hai ya koi aisay sawalat hotay hain jis se zachki depression ki baat karen? |  |
|  |  |
| Interviewee: History pe toh wo likhtay hain history paper hotay hain uspe wo sari history |  |
|  |  |
| Interviewer: History main kia koi aisay khaas sawal hotay hain jo depression keh mutaaliq  hon? |  |
|  |  |
| Interviewee: Hotay hongay liken usme wo mention kardetay hain doctors ye sab | Maybe history taking form has such questions  Doctors mention all answers |
|  |  |
| Interviewer: Sahi hai. Aap aik hospital keh setup keh andar aik hospital main aisi kia wajoohat hain jiski waja se shayad zachki depression dikhayi bhi na de aur ussay diagnose bhi na kia jaaye? |  |
|  |  |
| Interviewee: Dubara bataye please |  |
|  |  |
| Interviewer: Aisi kia cheezen aik hospital keh andar aap kaam kar rahay hon |  |
|  |  |
| Interviewee: Jee |  |
|  |  |
| Interviewer: Aisi kia wajoohat hongy jiski waja se aik patient ki history main zachki depression dekha bhi na jaaye diagnose bhi na ho? |  |
|  |  |
| Interviewee: Aisa koi hota toh nhi hai | No factors contribute to no diagnosis or wrong diagnosis |
|  |  |
| Interviewer: Aisa kabhi hota hai keh history le rahay hain aur 10 patient aur aa ker kharay hogaye hon toh jaldi jaldi bas history lely bas main chaar sawal pooch keh agay barh gaye? |  |
|  |  |
| Interviewee: Nahi aisa nahi hota. Agar farz karen koi junior doctor hai wo history lerhy hain aur unko uspe patient keh thoray se maslay masaayal lag rahay hain to wo foran se senior keh paas jaty hain consult karnay keh lye | Junior doctors consult senior doctors immediately if there is discrepancy |
|  |  |
| Interviewer: Sahi. Aur aisay apko lagta hai koi hamaray pas monitor karnay keh lye - keh jo patient reh rahy hai unko bar bar dekhtay rahen? Iskay lye aisi koi tool hai ya koi aisay hain hamaray paas cheezen jis se hum pata kar saken |  |
|  |  |
| Interviewee: Jee |  |
|  |  |
| Interviewer: Keh zachki depression hai ya nahi? |  |
|  |  |
| Interviewee: Jee bilkul. Doctors hamari jo hain wo matlab keh unko jab lagta hai keh koi depression main hai toh uska wo half half hourly ya two two hourly ki wo unho ne poora paper banaya hua hota hai uspay wo bar bar jaa keh reading krty hain jesay phir unko lagta hai | ½ or 2 hourly chekcups are done |
|  |  |
| Interviewer: Kis cheez ki reading karty hain? |  |
|  |  |
| Interviewee: Jesay blood pressure hogya pulse hogya unka theek hai | NP, pulse reading is done |
|  |  |
| Interviewer: Sahi |  |
|  |  |
| Interviewee: Aur iskay elawa jesay keh patient ko depression hai toh depression keh lye specially nahi hai liken main apko bata rhy hun depression main jab wo hoty hain toh mostly wo hoty hain to unpe effect karta hai blood pressure hota hai ya sugar ka koi patient hota hai ya phir iss tarhan | No special checkup for depression  IF BP or sugar acts up, it is a sign |
|  |  |
| Interviewer: Toh koi referal hai matlab referal hota hai hospitals main |  |
|  |  |
| Interviewee: Jee jee bilkul. Jesay aik ward se doosri jaga doosri ward main toh jee bilkul refer kartay hain | Referrals are in place |
|  |  |
| Interviewer: Achaa, aur agar pata chalay keh bhaai iss maa ko hai depression toh phir kia hota hai? |  |
|  |  |
| Interviewee: Phir uski treat karwatay hain | Patients with depression are treated |
|  |  |
| Interviewer: Kis se treat hoty hai? |  |
|  |  |
| Interviewee: Jesay ya toh patient ko relax ya pehlay toh first of all toh patient ko matlab keh guide kia jata hai keh aap aisay nahi karo iss se aap per bhi aur apkay bachay per bhi asar parega. Theek hai, agar to wo matlab keh matlab aisay tension lerhy hain liken agar usko depression shuru se hi hai matlab keh koi aur wajoohat hain toh uskay lye toh doosri ward main refer kartay hain | Patient is counseled by doctor  If tension is part of her history, she is referred |
|  |  |
| Interviewer: Sahi. Maaon ko zachki depression se bachanay keh lye hum kia kar saktay hain? |  |
|  |  |
| Interviewee: Hum unko yehy guide karengay  keh aap tension nhi lo aap aisi condition main ho aap apna khayal rakho agar in case unko depression hota bhi hai toh ussay aap nazar andaaz karo ya phir uska solution batayengay keh aap keh aap is tarhan nhi karo apnay husband keh sath apnay gharwalon keh sath aap baat karo toh iss tarhan | Guide them not to stress out  Tell them to take care of themselves  Donot pay attention to your depression  Talk to family and husband if not easy to ignore |
|  |  |
| Interviewer: Aapnay kaha nazar andaaz karo toh agar maa nazar andaaz karde keh mujhay depression hai toh aapko lagta hai wo chala jayega? |  |
|  |  |
| Interviewee: Relax karegy to meray khayal main behtar hojayega | If she relaxes, it will get better |
|  |  |
| Interviewer: Behtar hojayega. Sahi. Apko lagta hai keh koi therapy hai jis se hum maaon ki help lr saktay hain? |  |
|  |  |
| Interviewee: Jee bilkul. Jesay wo agar har wakt ghar pe rehty hain to wo unki family main koi aisa hai jo aisa hai matter to aisay wo kisi aur tarf dihaan laga sakty hain keh aap isko choro ye kaam karlo ya aap thoray time keh lye chalay jaao matlab apni ghar family keh paas toh iss tarhan meray khayal main wo relax kar sakty hai | She can busy herself with other work  She can visit family who can take care of her |
|  |  |
| Interviewer: Sahi. Aur apko lagta hai groups bana ne se support group bana ne se koi fark parega? Keh bohat sari maaon ko prenatal period main aik saath bithaya baaten karayeen. Uss se koi fark parega? |  |
|  |  |
| Interviewee: Jee bilkul hota hai aisay. Abhi bataty hun abhi upar madam bol rhy theen keh ward main rush bohat zada hai toh hum inko agay waly ward main shift kardetay hain to jin jin patients ki apas main aik doosray ki achi ban rhy thy wo in main se bolrhy thy keh main iss ward se nahi jaongy yahan pe sabkay sath achi banrhy hai main doosri ward main nhi jaongy | Support groups count  Patients had to be shifted due to lack of space  Patients who had developed bonds refused |
|  |  |
| Interviewer: Sahi |  |
|  |  |
| Interviewee: Toh aisa hota hai keh community pe bhi bara effect parta hai | The community patient is in affects her |
|  |  |
| Interviewer: Sahi. Koi aur baat jo aap batana chahen iss hawalay se? |  |
|  |  |
| Interviewee: Yehy keh patients toh bohat aisay hain jo poor patient hain jinko need hoty hai toh wo hospital main atay hain to unko achi guideline mil jaty hai hum logon se aur ye keh behtri bhi lanay keh lye koi program aisa hona bhi chaiye inke honay chaiyen jin main patients ko relax kar sako. Last time iss tarhan tha aur wo depression pe tha aur infection control pe tha toh patients bhi gaye thay udher hamaray sath | Poor patients should get good guidelines  Programs should be arranged  Program was conducted on depression and infection control  Mothers and staff was invited |
|  |  |
| Interviewer: Sahi |  |
|  |  |
| Interviewee: Patients bhi gaye thay wahan pe aur unko bhi sath sath bataya tha keh aap apnay aapko falaan cheez se bachanay keh lye ye kaam karo | Patients were counseled about pregnancy behaviors |
|  |  |
| Interviewer: Sahi |  |
|  |  |
| Interviewee:Toh iss tarhan hoskta hai |  |
|  |  |
| Interviewer: Theek hai, bohat bohat shukrya aapka |  |
|  |  |
| Interviewee: Thankyou |  |

| Shakila – Nurse  *Briefing provided - verbal and written consent obtained* | |
| --- | --- |
| Interviewer: Mera sabse pehle apse sawal yeh hai k kia aap batasakti hain k apko is baat say kia samajh aata hai k maa ko kia health problems aate hain, maternal depression k doraan? |  |
| Interviewee: Woh saari ajati hain aur uski nutritional pattern change hojati hai jiski wajah say woh anemia main chalijati hain aur uske ilawa depression ki wajah say hypertensation main chalijati hain, aur kuch aise patients hotay hai jo after delivery psychosis main bhi chalejate hain. | Change in nutritional pattern  Enhances anemia, hypertension  Psychosis  Psyhcosis can occur after delivery |
| Interviewer: theek hai iske ilawa kuch? |  |
| Interviewee: iske ilawa bas yhi hai k matlab depression sabse bari baat aaj kal economical bhi hai aur uske ilawa yeh bhi k hai log k zehn main concept hai, male female babies ka to aksar gharon main yhi rojhan hota hai k female baby nhi chaheiy. Ab ladies ko yhi depression hai k agar mujhe female baby hogi to agay ghar wale meray sath kia sulook karengay. | Economic determinants  Fear of producing female offsprings - |
| Interviewer: Bilkul bilkul. To umm koi aisa case jo apko yaad ho jisne aap pay koi impact chora ho, jiski wajah say aap bohot mutasir hoi hon? |  |
| Interviewee: Nahi as such to meray samne nhi hoa lekin yeh zaroor hoa hai aik martaba hamare samne k aik ladies thi jisne female baby ko matlab deliver kia, normal delivery thi to aur aik aur ladies thi usne male baby ko deliver kia, usne apna bacha exchange krdia tha. | Mother exchanged her female child with a male child in ward |
| Interviewer: Acha |  |
| Interviewee: Bacha exchange krlia tha lekin Allah ka shukar hai hamare Jinnah ki security aur saari cheezein record hamara itna acha hai k us baby ko hamne baziab karwalia tha. |  |
| Interviewer: Sahi. Theek. |  |
| Interviewee: Aur aik aur yeh thi aik martaba meray samne yeh waqaya bhi hoa tha k aik ladies thi jisne kabhi bhi baby paida hi nhi kia tha usne aake hamare pay ilzaam lagaya k mera baby yahan hoa hai kabhi falan nurse nay mera bacha lia hai ya kabhi falan doctor nay lia hai, to uspe kaafi media wale bhi aaye thay, shor sharaba bhi hoa tha phir madam nay matlab examine ki to pata chala k woh patient hi barren thi. Phir bhi nhi maane uska civil hospital say examine karaya wahan par bhi yhi kaha k barren hai, na isne bacha paida kia na paida karegi. Phir bhi nhi masne phir Aga Khan say bhi karwya phir uske baad phir hamare probation and police karwake case band karwadia. |  |
| Interviewer: Sahi, acha jee tou zachki depression k baare main apki kia samajh hai thori si wazahat karein? |  |
| Interviewee: Samajh yeh jaise primary gravida jo hotay hain unko sabse ziada yeh depression hoti hai k pata nhi hamare sath kia hoga, labour room main kis trhan bacha paida karwaengay, yeh saari cheezein aik primary gravida main kyunke awareness nhi hoti logon main aur kuch ghar wale batate nhi hai aur baaki hotay hai unke economically yeh k bhai ham jaengay to hospital ka mahol kaisa hoga to yeh blood pressure kisi ka barha hota hai usme bhi woh pareshan hoti hai, blood anemia ki wajah say aati hain to issi soch main bechare pareshan bohot ziada hoti hain. | Lack of awareness scares mothers  Economic issues  Enahnced BP, Anemia and stress levels  Mothers are more prone to depression in 1^st^ pregnancy due to lack of awareness regarding pregnancy procudures |
| Interviewer: Tou mashre main is maa k depression ka kia bhoj hota hai? |  |
| Interviewee: Mashre ka to sabse bara role mashre ka hi hai ... | Society plays the most important role |
| Interviewer: Theek hai. |  |
| Interviewee:Kyunke mashra hi aik aurat ko depression ki taraf lekay jata hai, aksar yhi hota hai k bhai agar kisi k bache nhi horhe ho uspe bhi depression aur woh bacha paida bhi krhe hain to usme economically itna depress hotay hain k agai ham bachon ka k kaise karengay guzara aur pregnancy main during pregnancy unki diet wagairah proper nhi hoti woh diet apna kaise arrange karein, hospitsal wale to sirf medication dedengay, delivery krdengay lekin aana jana yeh sara kuch bohot burden hota hai unpe. | Society is responsible for mother’s depression  Economic determinants such as payment for delivery, diet, transport, child’s needs  Poor diet during pregnancy |
| Interviewer: Sahi.. To mashre pay kia asar hota hai jab maa ko depression hojata hai? |  |
| Interviewee: Maa ko depression hojata hai to mashre main yhi asar hota hai k matlab jab maa hi sahi trhan say proper diet nhi lepaegy woh hamein healthy baby kahan say degy? | Baby’s diet is affected |
| Interviewer: Sahi to healthy baby nhi hoga to kia hoga? |  |
| Interviewee: Healthy baby nhi hoga to hamare mashre main us healthy baby ko survive krna hi mushkil hoga, unhealthy baby ko survive krna hi mushkil hoga na. | An unhealthy baby cannot survive |
| Interviewer: Tou agar baby survive nhi krta  to kia hoga? |  |
| Interviewee: Bohot kuch hoskta hai |  |
| Interviewer: Kia itni ziada maaon ko depression hai kai masla hai k bache hi khatam hojaengay? |  |
| Interviewee: Nahi bache to khair woh khatam nhi hongay woh to allah talah ki hi dein hoti hai lekin yeh hota hai k depression to hota hi haina woh to sabse ziada depression labour ka hi hota hi unko. | Labor stress induces depression |
| Interviewer: sahi to prenatal aur postnatal jo kehte hain depression hota hai uski apki kia samajh hai kia hota hai woh? |  |
| Interviewee: Prenatal aur postnatal. Postnatal yhi hota hai k unko bohot saari aisi aurtein hoti hai jinko matlab female baby kaise deliver hogyi usme hi depress hojate hain aur kbhi yeh hota hai k during pregnancy unka BP high hogaya tha to uske baad bhi usko  maintain krne main pareshan hotay hain k kaise karna hai medicine kaha say lani hai. Sabse ziada role to economical hota hai. | Postanatal is after birth  Stress of following the right delivery procedures |
| Interviewer: Sahi |  |
| Interviewee: Economical aur education. Uneducated hotay hai jinko pata hi nhi hota k ham apni matlab kaise sehat ka khayal karein. | Uneducated mothers donot know how to take care of themselves |
| Interviewer: Hmm |  |
| Interviewee: Jab woh apni sehat ka hi... |  |
| (Long pause) |  |
| Interviewer: Sahi jab woh apni sehat ka hi? |  |
| Interviewee: jab woh apki sehat ka hi khayal nhi rakh sakte woh kaise maintain karengay apne aap ko aur feeding main bhi hota hai bohot sare logon ko yeh hota hai k breastfeeding ham kaise karaein jaise matlab primary gravida hai pehla bacha hai usko samajh hi nhi arhi feeding karane main bhi unko bari mushkil hoti hai. | Mother is unable to look after herself  Lack of knowledge about procedures such as breast feeding |
| Interviewer: To main yeh janna chahongi k apke hisaab say jo yeh zachki depression hota hai prenatal ka kia matlab hai aur postnatal ka kia matlab hai? |  |
| Interviewee: Prenatal ka matlab yeh hai k delivery say pehle aur postnatal ka matlab hai delivery k baad. | Prenatal is before delivery  Postnatal is after delivery |
| Interviewer: To kia prenatal aur postnatal dono main hota hai? |  |
| Interviewee: Ziada tar prenatal main hota hai postnatal main thora sa relax hojate hain patients. | Prenatal period has higher chances of depression as compared to postnatal  Mothers stress levels go down during postnatal period |
| Interviewer: Sahi hai |  |
| Interviewee: Bohot kam aise patient hotay hain jo ziada depress hotay hain ziada wohi hotay hai jo chlamydic hotay hain aur chlamydia ki wajah say phycosis main chalejate hain lekin ziada tar pre hota hai post bohot kam hota hai. | High rish physical ailments can induce depression |
| Interviewer: Acha jee aur iske alamat kia hain zachki depression k? |  |
| Interviewee: Zachki depression k alamat aik to yeh k patient ka behavior hota hai | Mother’s behavior alters in depression |
| Interviewer: Sahi hai |  |
| Interviewee: Aur behavior agar uski change hojati hain usko kuch samajh hi nhi aati aur uske ilawa yeh k... Aur kia hai (laughs) |  |
| Interviewer: Koi baat nahi…koi bhi alamat jo apne dekhein ho jo apko samajh aati hon? |  |
| Interviewee: Maine to yehi dekhi hai aksar matlab behavior ka hi ziada change aati hai aur jab ham _______ krte hain to usme bp bhi barha hoa hota hai. | Enhances BP |
| Interviewer: Tou kis tarhan ka behavior change hota hai? |  |
| Interviewee: Irritate hotay hain na thoray sai jaisay unko kuch samjh nhi aarhee, ghabrahat unko ziyaadah hotee hai, ghabratain keh pata nhi ab kia hoga hamaray saath kaisay hoga aur us doraan agar ham unka bp check kartay hain keh unka bp barha hua hota hai aur kuch tou aisay hotay hain tachycardia bhi hojata hai unko keh pulse rate bhi unki ziyadah hojaati hai. | Irritability, Palpatations during pregnancy  Enhances BP  Can lead to tachycardia |
| Interviewer: Sahi, time duration kitne arse chalta hai yea zachki depression? |  |
| Interviewee: zachki ki depression ka yeh jab woh relax hojate hain baby deliver hojata hain na to adhay say ziada unki depression kam hojati hai jaisi baby deliver hojate hain baby ko dekhte hi maa ka mood set hojata hai. | Depressive symptoms disappear in postnatal period  Mother’s depression is cured once she sees the baby |
| Interviewer: Acha to yeh kitna time frame hota hai ya kitne maheenay hotay hain jab prenatal depression hota hai aur postnatal depression hota hai? |  |
| Interviewee: Prenatal yeh k takreeban jaise term kareeb hotay hain inke to third trimester aata hai to usme uske matlab pareshanian barhti jaengy aur jaise during…delivery k baad relax | Prenatl refers to third trimester |
| Interviewer: Aahi theek hai. Maao main sabse ziada risk kisme hota hai, zachki depression ka aur sabse ziada khatra kisko hota hai? |  |
| Interviewee: Maao ko meray khayal main ziada hota hai. |  |
| Interviewer: Kis trhan ki maa ko? |  |
| Interviewee: Jiska bp high ho. | Mother with high BP is at high risk |
| Interviewer: Acha aur? |  |
| Interviewee: Bp main jiska uric acid barha ho, urinal problems positive ho protein urea agar ho to usme maa ko bhi ziada problem hoti hai. | Mother with complicated pregnancy is at high risk |
| Interviewer: Aisi maaon ko hi hota aisi khatra hota hai aisi depression ka? |  |
| Interviewee: Depression ka unko khatra to hai. |  |
| Interviewer: Aur aisi konsi maa hain jinko ziada khatra hai k unko jaldi zachki depression hoga? |  |
| Interviewee: Jaise cardiac patients aate hain pregnancy main during cardiac to hotay hi hain to unko yeh hota hai k cardiac hain ham hamein pata nhi kia hoga bacha kaise paida hoga ya jaise kisi ka placenta previa pata chaljata hai usme main bhi woh pareshan hoti hain | Mothers who suffer from severe physical illness can fall prey to depression because of a never ending list of fears |
| Interviewer: Sahi hai to yeh sabse ziada jo apke hisaab say ziada tar maa jo hoti hain jinko khatra hota hai woh ziada tar physically sahi nhi hoti hain k kia yeh medical problems hotay hain? |  |
| Interviewee: Medical problems yhi hongay hypertensive hongay ya diabetic hongay yaa unke labs main abnormalities ajati hain. | Mothers with physical complications are at high risk |
| Interviewer: Kia zachki depression ko manage karskte ho? |  |
| Interviewee : Manage krskte hain agar ham unki counseling achi karein to | Counseling can help mange it |
| Interviewer: Aur kia krskte hain counseling k ilawa? |  |
| Interviewee : Counseling krskte hain aur har tarhan say matlab physiotherapy jisse ham kehte hain k matlab agar woh physiotherapy bhi karate hain apni exercise hoti hai usme prenatal ki usme bhi patient kaafi ziada relax hojata hai | Relaxation exercises to help ease pregnancy complications during prenatal period |
| Interviewer: Short term mukhtasir aur long term yani lambe arse tak k jo maslehat aati hain maaon ko zachki depression say apke hisaab say woh kia hoti hai? |  |
| Interviewee: Short term to ziada tar hotay hain long term to utne nhi hotay hamare paas…long term tou itnai naheen hotai | Higher chances of issues during short term |
| Interviewer: Short term main maa k sath kia masle hotay hain? |  |
| Interviewee: Yehhi same masle jo maine apko batadiye hain matlab bp high hona, irritate hona, restless rehna, neend na aana | Sleeping issues  Irritability and restlessness  High BP |
| Interviewer: Aur uski wajah say maa ko kia asar prha hota hai? |  |
| Interviewee: Maa ki health ko hi effect hota hai bache ko proper tareekay say feed bhi nhi karasakegy bache ki sahi care bhi nhi karpaegy | Child misses out on proper feeding regime  Lack of care towards child |
| Interviewer: Yeh to bache pay asar parha hai k feed nhi karayi diet nhi karayi, isme maa pay ki asar parha hai? |  |
| Interviewee: Maa apna bhi khayal nhi krskegi usko infection hojaega, diet apni proper nhi lesakti usko weakness aur anemia ki taraf bhi jaskti hai | Lack of self care  Poor dietary patterns leading to complications such as weakness and anemia |
| Interviewer: Apko lagta hai isse family members par effect hota hai iska? |  |
| Interviewee: Agar unke family members cooperative hain unke sath aur agar sincere hain unke sath to unko zaroor asar hota hai lekin kuch families aise hotay hain k bas woh apni beti ya bahu ignore krletay hain to us family par itna asar nhi hota kyunke hamne aksar logon ko dekha hai k yahan aake relax hojate hain na blood arrange karaengay, medicine proper lake dengay jo cheez available hai woh to dedengay jo nhi hai woh ham unko likh k dedengay magar woh nhi laate woh kehte hain aap log ka kaam hai aap log karo | Cooperative and sincere family memebrs will be affected negatively  Some families donot care about the mother’s health and so remain unaffected  Most families donot even make the necessary arragments needed to save the mother’s life  Most families refuse to take accountability for mother’s condition |
| Interviewer: Tou family pay kia asar prta hai k agar kisi maa ko zachki depression hai to us family pay kia asar paarta hai? |  |
| Interviewee : Family ka yeh k family bhi unke sath sath suffer karega itne din hospital main uski care main lagjaengay baaki jitni dosri duties wale hain woh duties pay nhi japaengy aur economically crisis main bhi ajaengay | Family’s economical condition suffers  Time constraints affect family’s ability to overlook their personal responsibilities  Breadwinners are unable to go to work |
| Interviewer: Sahi to kia tamaam jitne bhi dekhbaal krne wale hotay hain aik maa k ya jitne bhi unke husband ya ghar wale sab pay farq parta hai agar maa ko zachki depression ho? |  |
| Interviewee: Agar uske pehle bache haina sabse pehle un bachon pay asar hoga | Other older children suffer more than other caregivers |
| Interviewer: Sahi |  |
| Interviewee: Uske baad ghar k baaki members pay hota hai agar matlab uske second gravida ya third gravida hai to un bachon pay ziada asar hoga woh bache bhi ignore hongay | Older children are affected the most |
| Interviewer: Sahi |  |
| Interviewee: Na unko proper time milega maa ka na hi unka dehaan education ko taraf hoga aur khud depress hongay k hamari maa ko hoa kia hai | Mothers are unable to overlook the needs of the older children  Education of the older children suffers  Children will remain worried because of lack of understating of their mother’s condition |
| Interviewer: Sahi |  |
| Interviewer: Tou apke hisaab say pakistan main zachki depression ko manage kaise kia jata hai? Ya nhi kia jata? |  |
| Interviewee: Maine koi khas manage maine nhi dekhi depression main yhi hota hai bas counseling yeh agar rakhi ho aur sahi trhan say unko samjhaya jaye aur agay samajhdar logon say samajh jayein to kuch hoskta hai otherwise maine to aisi koi khaas cheez nhi dekhi | Counseling can be helpful only if it works for involved parties |
| Interviewer: Sahi |  |
| Interviewer: Tou koi aise tools hain hospitals main jiski wajah say aap diagnose karsako? |  |
| Interviewee: Diagnose tou matlab iski yhi physical examination hi ham log krletay hain physically hi hamein pata chaljata hai k matlab depression main hain depress hain us tareekay say hamein baat krte hoye pata chaljata hai kitni depress hain | Physical examination helps understand if mother has depressoion  Talking to mother can help assess severity |
| Interviewer: Sahi kia kabhi initial screening hoti hai jis main maaon say poocha jaye, maa pehle pehle aati hai koi parcha bhara jata hai jis main poocha jaye k bhai koi usko masle hain ya nhi hain? |  |
| Interviewee: Antenatal main? |  |
| Interviewer: Antenatal main. |  |
| Interviewee: Antenatal main history to letay hain |  |
| Interviewer: Sahi |  |
| Interviewee: History letay hain lekin itna matlab depression ki taraf koi nhi jata | History and examination doesnot focus on depression particularly |
| Interviewer: Acha aur koi apne tool dekha hai jo k istimaal kia gaya ho? |  |
| Interviewee: Nahi | Have never seen or used tools |
| Interviewer: Kia Pakistan main hum treat krskte hain ya manage krskte hain... |  |
| Interviewee: Bilkul krskte hain | Can be treated in Pakistan |
| Interviewer: Kis trhan say. Kia alag alag tareekay hain jo apne dekhein hain jisse kia jata ho? |  |
| Interviewee: OPD  k lihaaz say bataon ya ward k lihaaz sy? |  |
| Interviewer: Generally aur OPD ward dono k lihaaz sa . |  |
| Interviewee : Generally yh hona chaheiy aik to yeh k hamare Karachi main Jinnah Hospital ki baat karein to rush itna hota hai k aik pregnant aurat rush dekh k hi woh sabse pehle depress hojati hai k bhai mera number pata nhi kab aega. Unko agar proper sahi time say manage kia jaye aur har room main unko bari bari counseling aur achi trhan say.. Sabse bari baat hai unka apna .. Aik to language barrier to hamare paas bohot hi hai, har zubaan k log aate hain agar unko un hi ki zabaan main ham samjhadein k jaise punjabi hai ham punjabi main bolein sindhi k sath sindhi boldein to us cheez say bhi kaafi  farq parta hai aur proper unko har cheez agar sahi trhan say mile dakham dakhi na ho to relax hojaengy aurtein aur uske leye zahir hai log bhi itne hi chaheiy , log bhi short hai hamare paas | Hopsitals are overcrowded  Mothers have to wait for long hours affecting their health  Mothers are not counseled properly due to time constraints during their visits  Counseling is only affective if provided in the mother’s local cultural language  Diagnosis procedure is hurried due to overcrowding  Availability of HCPs is not proportional to the number of pateints |
| Interviewer:Sahi |  |
| Interviewee: Sabse bari baat yeh shortness hai k doctor ki bhi shortness bohot hain, staffon ki bhi hain, paramedics bhi nhi haim hamare pas, jab shortness hogi to kaam nhi hoga | Doctor, staff and paaramedic shortages make the situation worse |
| Interviewer: Tou agar aap kehrhi hain k counseling hoti hai. Agar hoti hai to apko lagta hai k maa ya families jaati hain lenay k leye yeh counseling? |  |
| Interviewee: Dekho counseling lenay k leye to koi nhi jata. Jab apke pas hospitalized hota hai patient us waqt aap uski counseling krskti hain ya OPD main apke pas visit main agye to us waqt usko counseling krskti hain | No one likes to go for counseling  Patienst can be counseled when they visit doctor in OPD or are hospitalized |
| Interviewer: Sahi to aap kaise counsel karogay unhe? |  |
| Interviewee: Sur dosra tareeka yeh hai k agar poore overall Pakistan ki baat ki jaye jaise community midwives hoti hain unko community main jana chaheiy. Jis ghar main matlab is trhan ki ladies hoti hsin unko achi trhan say counseling, unko unki care k baare main, unki vaccination k baare main, bache ki health k baare main , feeding k baare main har lihaaz say agar unko counseling karein to mera khayal hai yeh cheezein bohot kam hongi | Community midwives can play an important role in helping pregnant mothers  If mpthers are counseled well about all the process involved in giving birt, it can help reduce chances of depression |
| Interviewer: Sahi to aisi kia cheezein hain jo aap istimaal karengy aik maa ko counsel krne main? |  |
| Interviewee: Community base pay? |  |
| Interviewer: Jee. |  |
| Interviewee: Community base pay yeh hota hai k jaise aik ilaqay k community unki bakaeda aik team hoti hai un team k paas poore ilaqay ki registration honi chaheiy aur jitni pregnant women hai unki registration honi chaheiy unki agar daily nhi hoskti weekly unki matlab visit honi chaheiy.Patient to nhi aega community main aapke paas. Community k staff, midwive ya doctor uko khud jana hoga door to door | On a community level, all pregnant mothers can be registered with a team of community midwives  Counsneling can be provided, if not on daily then on weekly basis to mother.  Patients donot take the initiative of visiting a doctor or community staff  Sysytems need to be in place to ensure that no mother misses out on being counseled  A door to door outreach system needs to be established |
| Interviewer: Lekin hamare yahan jo silsila to apko lagta hai k community main midwives hoti hain ya jaati hai ya direct hospital ajata hai patient? |  |
| Interviewee: Community midwives to hoti hain lekin yeh k kam tadaad main hoti hain mujhe to nhi lagta hoga k unki posting bhi hoti hogi ya phir koi jata bhi hoga, koi nhi jata | We already have community midwives but the are less in number  Most community midwives are not working on ground |
| Interviewer: Sahi to jab patient hospital ajata hai to usko counsel kaise kia jata hai? |  |
| Interviewer: Hospital aata hai.. Hospital main to wohi matlab starting hi unki gate say hi hojati hai unki depression wohi say start hojati hai jaise rush dek k stress main ajati hain jab tak woh apna bp wagairah check krleti hain us doraan bhi agar jo staff duty pay baithi hain woh unko thora sw counseling krlein aik agar aik jo uski history lerhi hai woh bhi krlein issi trhan baari baari jis jis k paas jaati jarhi hain har koi uski bilkul positive counseling kare to mera khayal hai na ho | Mother should be counseled by all the hospital staff members she meets during her visit |
| Interviewer: Sahi, apke hisaab say aisi kia wajoohaat hain jiski wajah say aik maa itne ahem masle k leye bhi doctor k pass nhi jaati? |  |
| Interviewee: Woh isleye nhi jaati kyunke economical problems hotay hain, unke paise unke paas nhi hotay travel krne k, k gaari nhi hai to kaise jayein aur agar chalein paise bhi agaye lekin unke sath aane wala koi nhi aur bachon ko dekhne wala koi nhi hota isleye phir woh yhi prefer krte hain aur bache ko bhi deliver karwadetay hain | Economic determinants stops mothers from acquiring care  Inaccessibility of transport and child rearing services hinder mother’s ability to seek care |
| Interviewer: Sahi iske ilawa yeh to delivery hogaya na, magar delivery k hisaab say thora samjhayein k zachki depression k leye koi kyun nhi jata? |  |
| Interviewee: Woh nhi jaate kyunke aksar logon ka yeh khayal hai k depression patient jo hain unpe rohani asrat hain ya jinn bhoot k asrat hain aur yeh ispe ziada yaqeen karte hain aur yeh doctor k paas aane k ilawa mualvion k paas jana ziada pasand karte hain, falana jagah say tahweez leni hai falana jagah say yeh woh k ham sahi hojaengay. | People belive it’s a religious issue – supernatural powers are blamed for mother’s condition  Most mothers seek help from religious scholars |
| Interviewer: Apko lagta hai k yeh isleye bhi hai kyunke hamare paas jo hai society main mana hai jana ya society accept nhi karti khatoon ko? |  |
| Interviewee: Nahi society main mana to nhi hota magar yeh apna personal concept hota hai na |  |
| Interviewer: Kia personal concept? |  |
| Interviewee: Personal yhi hota hai k jaise woh choro doctor ko lagta hai yeh jinnat k asrat hain phir woh us taraf jana pasand karengay to woh doctor k paas kam jaengay kyunke unko lagega k doctor sirf taakat ki goliyan likh kar dedengay usse bacha aur mota hojaega tabiyat hamari aur kharab hojaegy. Awwal to woh medicine khaengay nhi aik to woh us wajah say nhi khaengay kyunke bacha healthy hojata hai aur log yeh nhi sochte k hamari nutrition diet achi hogi aur dosri us wajah say k dosron k pas paise nhi hotay, arrange nhi karpate | Mothers prefer to visit the doctor less and a religious scholar more  More money is needed to seek help from a doctor as compared to a religious scholar |
| Interviewer: Apko lagta hai k agar maa jana bhi chahe to itne log hain k saari maaon ko jo hai screen krske unko dawai desakein? |  |
| Interviewee: Nahi hamare pas itni nhi hai | Shortage of HCPs in the gynecology ward |
| Interviewer: Sahi to hamare pass apke hisaab say bohot kam log hain ya bas normal log hain ya...? |  |
| Interviewee: Meray hisaab say matlab gynae/obs k lihaaz say bohot kam hain |  |
| Interviewer: Bohot kam hain…. Hmmm… Tou aik patient jisko zachki depression jo aik maa hai woh kia sochegi apne baare main usse kaisa mehsoos hoga? |  |
| Interviewee: Usko to kuch bhi mehsoos nhi hoga kyunke woh smaajhrhi hai main jo koi bhi behavior changing usme ajati haina to woh samajhrhi hai main normal activity krhi hon lekin uske sath jo rehne wale hain unko uska ziada hota hai k bhai yeh larki pehle aisi nhi thi ab yeh aise kyun krhi hai | Mother doesnot realize that she is not acting like her everyday self  Family is able to catch the difference in the mother’s behavior and is affected |
| Interviewer: Sahi to maa ko kbhi mehsoos nhi hota? |  |
| Interviewee: Maa ko kbhi mehsoos nhi hota han bp barhjata hai unki headache hoti hai unko yhi kahengy hamein nazar nhi arha hai lekin unko yeh nhi pata k yeh hamein kis wajah say hamare sath horha hai yaa jaate hain to sans phoolta hai unko yeh nhi pata hamein anemic hai | Enhanced BP, headaches  Mother’s condition is commonly belived to be a consequence of evil eye  It is belived to be caused by the physical condition |
| Interviewer: Sahi to apko lagta hai k Pakistan main jo medical framework hai jo aap seekhte hain hamlog uska koi uska koi lena dena hai is sab say uski wajah say management pay koi asar prta hai zachki depression k? |  |
| Interviewee: Nahi sawaal nhi samjhi apka |  |
| Interviewer: Hamara jo medical framework hai jo ham medical prhte hain apne bhi prha hoga 4 saal nursing ki hogi. Apko lagta hai k uska koi asar hota hai k bhai k trhan aik maa ko lekay chlna hai jisko zachki depression hai? |  |
| Interviewee: Usme to hamein sikhaya jata haina zachki depression main kaise ham patient ko manage karengay kia karna hai usko bilkul confidence main matlab confidence uska bilkul bahaal karna hai k bhai ham apke sath hain aur yeh saari cheezein medical main hamein sikhayi jaati hain lekin baat yeh hai k usko apply bhi to krna haina | Medical curriculum emphasizes on ensuring that the mother feels confident and supported  Difficult to apply guidelines to real life because of overcrowding |
| Interviewer: To apne jab apna parha tha nursing to apne yeh poori cheez parhi thi k zachki depression kia hota hai kaise hota hai kyun hota hai kaise usse hal krna hai masla? |  |
| Interviewee: Bilkul |  |
| Interviewer: Yeh sab apne parha tha |  |
| Interviewee: Jee |  |
| Interviewer: Acha jee lekin aap batarhi hain k kbhi apne istimal nhi ki yeh saari cheezein? |  |
| Interviewee : Main yeh nhi kehrhi k maine istimal nhi ki hai main overall baat karhi hon |  |
| Interviewer: Sahi |  |
| Interviewee: kyunke patient ka ratio itna ziada hota hai yahan par k aap aik patient ko itna time de hi nhi sakte | Patients cannot be provided with enough time due to overcrowding |
| Interviewer: Sahi to apko lagta hai k job pay apko kbhi sikhaya gaya k bhai zachki depression kia hai kaisa hai kyun hai? |  |
| Interviewee: Job pay to koi nhi sikhata job pay matlab khud banda waisi learn karleta hai dekh k yaa... | No on-job trainings  One can learn through observation |
| Interviewer: Sahi. Koi workshops ya trainings jisme apko sikhaya gaya ho? |  |
| Interviewee: Workshop depression k hisaab say to psychiatric main hamne ki hain lekin gynae main bhi gynae main yeh hota hai k aksar breastfeeding pay hi karwaletay hain aur bohot kam nurson ko involve krte hain | Nurses are usually not aksed to attend workshops  Workshops on depression take place in psychiatry ward only |
| Interviewer: Sahi |  |
| Interviewee: Nurson ko itna involve nhi krte gyny main | Nurses are not involved in Gynecological procedures mostly |
| Interviewer: Sahi to practically apne kabhi seekha ho khud say dekh kar k yaar maa bohot rorahi hai yaar kuch masla hai |  |
| Interviewee: Woh to dekho har insaan - har aik aurat k ander aik maa hoti hai | Each woman has a mother within her |
| Interviewer: Sahi |  |
| Interviewee: jab aik maa roti hai to jitna bhi sangdil banda ho usko to taras aahi jaega na to uski counseling to karletay hain. Meri duty to takreeban ICU main hoti hai mera to is tarhan k mareezon say bohot wasta parta hai woh gyny k lihaaz say bhi hota hi aur uske lihaaz say bhi hota hai ab woh depress hotay hain apne ghar main jo bachein hain unko chor kar aaye phir unko itne pyaar say samjhana parta hai k nhi tumhari tabiyat sahi hogi tum bachon ko dekhogy to unko ham time dedetay hain jaise main apni personally baat krti hon main apne ICU k patients ko time deti hon unke pass baithke main baatein krti hon, unke chotay chotay masle hain unko main hal karti hon unko heh main matlab aitemad deti hon k matlab meray haath main jitne bhi kaam hain main apke sath hon main krongi aur is cheez say bhi woh kaafi relax hotay hain aur aksar yhi hota hai woh mujhe subah main dekhte hi na khush hojate hain k hamare baaji agyi sunne wali koi hai | If a mother cries, any woman can empathize with her  Empathy allows for all women to counsel a crying mother  Hospiatlzied mothers can potentially get depressed for they are kept away from their children  Talking to mothers makes a huge difference  Mothers feel at ease when they are confident of the staff overlooking their case |
| Interviewer: Sahi |  |
| Interviewee: Lekin main yeh cheez har aik k baare main apko confidence say nhi kehskti har aik ka apna personal hota hai | Not all staff members are cooperative with mothers |
| Interviewer: Jee..Tou kia continuous medical education ka apne suna hai kabhi? |  |
| Interviewee: Suna to hai.. |  |
| Interviewer: Kekin kabhi apke sath hoa? |  |
| Interviewee: Nahi | Not attended CMEs |
| Interviewer: Acha. Apko lagta hai k aapki apni .. Aap usko kis trhan dekhti hai ya us baare main kia sochti hain to… Kia apko lagta hai k aap usko kaise dekhti hon usse bohot farq parta hai k aap patient say kaise baat karengy? |  |
| Interviewee: Sabse pehle yeh k hamein apna bhi behavior acha krna hota hai | Staff behavior counts a lot |
| Interviewer: Sahi |  |
| Interviewee: Agar aik patient hai aur depress hai aur depression main kuch bhi bole agar ham uske aagay waisi bolna shuru krengay mazeed uski problem main izafa hoga kam nhi hoga isleye hamein apna tone neechay karna hota hai hamein usko pehle samajhna hota hai usko pyaar say samjhana hota hai | Staff should not react when patients lose their calm  Mothers should be dealth with love and affection |
| Interviewer: Sahi |  |
| Interviewee: Aur usko is tarhan counseling karein k woh is problem say bahir ajayein yeh nhi k uske problem barhe |  |
| Interviewer: Tou khud afadiyat apko lagta hai k uska koi lena dena hai zachki depression say? |  |
| Interviewee: Hamara? |  |
| Interviewer: Aap ko lagta hai k apki responsibility hai yeh cheez k aap maa ko agar usko zachki depression apko lgrha hai k hai to usko screen karo usko sahi karo usko counsel karo? |  |
| Interviewee: bilkul yeh hamari matlab bilkul hamein karna chaheiy | Nurses are responsible for screening mothers |
| Interviewer: Theek hai |  |
| Interviewee: Aur karte bhi hain takreeban lekin yeh k document to naeen hoti hai who cheezein | Lack of documentation leads to loss of data |
| Interviewer: Apko lagta hai k shafqat ya hamdardi say koi farq parta hai ? |  |
| Interviewee: Bohot. Aap aik lafz pyaar ka boldein patient k sath kasam say uski adhi bemarian khatam hojati hain | Empathy plays a major role in curing mothers |
| Interviewer: Apko lagta hai k apko araam say aitemadi k sath sukoon k sath aap patient say yeh saari baatein krskte ho? |  |
| Interviewee: Bilkul | Talking to patients with confidence is not the issue |
| Interviewer: Aap usko bataskte ho k bhai apke sath yeh masla hai aur apko yeh karna hai? |  |
| Interviewee: Dekho kabhi kabar patient irritate hotay hain unko unke masail to ham nhi batasakte lekin yeh k unko ham itna support krskte hain k woh sahi jane wale hain sehatmandi ki taraf ajayein | Irritability  Supportive staff can relive a mother of stress |
| Interviewer: Sahi to kabhi kabar aisa bhi hota haina followup nhi hota doctor mareez ka sik connection nhi banta, apko lagta hai k usse zachki depression ka koi lena dena hai? |  |
| Interviewee: Bilkul bohot hai |  |
| Interviewer : Kia lena dena hai? |  |
| Interviewee : kyunke sabse pehli baat yeh k patient ka followup hi nhi hoga to aapko patient k baare main kuch nhi pata hoga agr aik visit ki phir akhir main delivery main aake usne karayi uske sath ghar main kia crisis hain kisi nay usko samjhaya ya nhi, vaccine usko nhi lagi, iron usko nhi mili proper nutrition k baare main kisi nay usko nhi bataya baby k baare main kisi nay nhi bataya woh to automatic usme to hoti hi hai | Patient follow-up plays a critical crole in diagnosis and management |
| Interviewer: Sahi to apko lagta hai k agar patient aata ho patient followup ho to aik gynecologist usko help krskti hai? |  |
| Interviewee : Problem k sath gynecologist bilkul help krskti hai | If patient knows her problem, a gynecologist can help instantly |
| Interviewer: sahi to agar patient aye aur apko lagrha hai k usse zachki depression hai to apko lagta hai k gynecologist uski counseling krskti hai? |  |
| Interviewee: bilkul krskti hai | Gynecologist can counsel patient |
| Interviewer: Kia general practitioners woh log honay chaheiy jinse sabse ziada pehle jinse rabta hota hai jin doctors say sabse pehla rabta hota hai apko lagta hai k woh log honay chaheiy jinhe pata  hona chaheiy k yeh kyun hain? |  |
| Interviewee:Nahi yeh to sabko pata hona chaheiy Zaroori thori hai k unko hi pata ho | Everyone who can beinvolved with a mother should be well –versed |
| Interviewer: sahi hai aur khud aik gynecologist kia krskti hai? |  |
| Interviewee:: Gynecologist aik patient ko support dai sakti hai us ko education dai sakti hai thori counselling kai zariyay aur agar koi patient admit hai usko kisi cheez ki need hai tou uskay paisay nhi hain cheezain nhi hai tou woh bahar kai kisi bhi fund sai ya donation sai ya zakat wagairah sai kuch bhi hai uski help karsaktay hain usko relax karakti hain | Gynecologist can help a patient to a certain extent |
| Interviewer : Tou yeh jo zachki Ki depression hai uska jo diagnosis ki ko management hai uski hospital main sai konsi cheez hai jissay usko asar horaha hota hai |  |
| Interviewee : Management pai? |  |
| Interviewer : Jee management pai diagnosis pai hospital ki aisi kia cheezain hain kia ... Hain jo tashkees par asar kartay hain? |  |
| Interviewee : Sabsay pehli baat tou yeh keh rush, bohot rush hota hai rush main aisay patient bohot ziyadah irritate hotay hain aisay patients kai liay alag sai matlab rooms hon yahan jo doubling kai patient hotay hain unko ziyaadah hee depression hota hai | Overcrowding  Insufficient number of beds for hospitalized mothers |
| Interviewer : Acha doubling sai matlab? |  |
| Interviewee : Matlab aik bed par do patient kabhi teen bhi hojatay hain ispay unki depression aur hee bharay gee keh bachay ko laitain khud kahaan laito main ghar chalay jaoon ussay acha aur woh apni saari cheezain bhooljaigee keh kitnay infection hain ya hypertensive hain | Disease can spread when mothers abe share bed during birth, deteriorating her health, leading to depression |
| Interviewer : Iskay ilawa aapko lagta hai keh hospital wagairah main patient ka screening hona ya na  hona aik role play karta hai? |  |
| Interviewee : screening tou hona |  |
| Interviewer : Aur pehli baar jo patient aata hai ussay aap sawalaat kartay hain keh kia hua kaisay hua kab hua  aap poochtay hain keh zach ki depression kai mutabiq koi sawaal pooch saktay hain? |  |
| Interviewee : Nahi saktay indirectly pooch saktay hain | Questions related to depression can only be asked indirectly |
| Interviewer: Jaisay kai? |  |
| Interviewee : Ghuma phira kar |  |
| Interviewer: Lekin aap poochtay ho? |  |
| Interviewee: Poochtay hain jaisay uski previous history poochaingain uski ecnomical history poochaingain | History revolves around understanding patient’s financial background and past history |
| Interviewer : Aap ussay especially jab history poochtay ho tou aap usko kehtay hain keh aapki koi dimaaghi masla hai ya kuch |  |
| Interviewee : Dimaaghi masla ham directly nhi kehsaktay | Psychological health is not directly addressed |
| Interviewer: tou aap unsay kis tarhaan poochtay hain ? |  |
| Interviewee: Jaisay unki environment kai baray main pooch saktay hain keh woh kahaan rehti hai garmi kitni percent hai ya ghar koi problem hai uskay baray main pata chal jaiga husband kai baray main aap pooch saktay hain lekin aap ussay directly yeh nhi kehsaktay keh aapko dimaaghi tawazun sahi nhi hai | A mother cannot eb told that she is mentally unfit |
| Interviewer : Aapko aisa lagta hai keh aisay koi tools hai ya aisay koi protocols hain ya nigraani ki jissay hamain pata chal sakta hai keh patient ko zach ki depression hai |  |
| Interviewee : Aisa tou kuch bhi nhi hai | No pro-active tools |
| Interviewer : Aur refer kaisay kartay hain keh agar usko hai hee hai tou usko refer kaisay kartay hain |  |
| Interviewee : Ham log tou refer kartay hee nhi hain balkay ham tou case ko laitay hain jinnah main tou jinnah sai refer nhi hotay |  |
| Interviewer : Ya agar kisi maa ka dikh raha hai keh usko zach ki depression aap uska phir kia karogay ? |  |
| Interviewee : Apni phycatrist ko call kartay hain, apni jinnah kai phycatrist ko call kartay hain woh hamaray patient ko examine kartay hain aur uskay hisaab sai treatment wagairah jo bhi prescribe kartay hain | Severe cases are referred to psychiatry ward |
| Interviewer: iska koi follow up hota hai kia? |  |
| Interviewee: follow up yeh hota hai keh patient jab tak hamaray paas hota hai woh visit karnay aingain doctor aur jaisi hamaray paas sai dc hotay hain tou ham apna dc kaat kai likh daingain tou further aapki treatment kaheen aur sai hogee | Once patient is discharged, no follow-ups take place |
| Interviewer: Maoon ki ham hifazat aur madad kaisay karsaktay hain zachki depression sai ? |  |
| Interviewee:  Ham unko yehi bas hospital kai hisaab sai itna hee karsaktay hain ghar tak tou koi jaa hee nhi sakta |  |
| Interviewer: Iska ilawa hum kia karsaktay hain? |  |
| Interviewee : kuch jagahon kai hisaab sai unkay education program bhi hotay hain aur agar gynee main bhi aisay education program hon pregnent patient ko aur agar ham unka role play bhi karlaingain woh bhool nhi jaingain ya aapnay presentation main rattafication karlee ya english main bol bol kar aagaye ab zaroori nhi hamaray patient saaray english samjhain ya agar ham unka koi program karain jaisain ham diabetic  program kartay hain kidney base ki kartay hain issi tarhaan gynee kai hawaalay sai bhi program hon tou usmain hamaray patient register hain aur jo bahar hai unko enroll karain jo bahar hon aur unkay hisaab sai unki zabaan main program karain unko samjhain aur agar us program main koi ya doctors koi role play karain ya kisi kai baaray main samjhain keh bahi yeh cheez aisay nhi aisay negative ki jagah positive cheezain batain tou meray hisaab woh kaafi hai | Educational programs  Role play activities can be helpful since visual clues are easier to remember  Role play is not dependant on language  Awareness sessions revolving round talks and presentations are not very effective due to language barriers |
| Interviewer: Tou aapko lagta hai aik hee program sai hojaiga kuch |  |
| Interviewee: Nahi aik hi program say to kbhi bhi nhi hota | Contiunuous awareness sessions are needed |
| Interviewer: Phir iske baad kia krna chahiye? |  |
| Interviewee: Iske baad further further matlab yeh continue krna hi chaheiy aur wardon main kahi bhi patient ka aik bakaida counseling room hona chaheiy jaise OPD hai OPD main bhi aik counseling room honi chaheiy | Counseling room should be established inside the gynecology ward and OPD |
| Interviewer: Sahi |  |
| Interviewee: Koi is tarhan ka patient hai aap usko counsel jo doctor krhe hain doctor hai ya nurse hain aap usko paas bhejo uski bilkul counseling karegi speech therapy hojaegy uski aik hisaab say aur coping stress ki taraf woh nhi jaskti | Counseling should help mothers express themselves and develop coping mechanisms |
| Interviewer: Sahi |  |
| Interviewee: Uska jo problem take woh negative ki taraf na jaye positive ki taraf aaye kyunke negative ki taraf jsegy to stress main jaegy |  |
| Interviewer: Apko lagta hai k negative ko positive rkhne k leye ham support group bansakte hain |  |
| Interviewee: Bilkul bilkul | Support groups can be useful |
| Interviewer: Iske ilawa apko koi therapy ka pata ho jo istimaal krskte hain |  |
| Interviewee:Isme coping stress ki hi therapy hoti hai isme alag alag steps hotay hain isme yeh hota hai k log aise hotay hain k jaise agar aap guzar rahi hain apne mujhe ignore krlia main is cheez ko yeh na sochoon k apne mujhe ignore kia hai hoskta hai aap jldi main thi | Stress coping therapy  Theraoy focusing on positive energy |
| Interviewer: Sahi |  |
| Interviewee: Tou is trhan ham agar uski soch main yeh daldein k nhi yeh cheez aisi nhi hai aap is cheez ko aise na sochein k usne apko ignore kia hai aap yeh sochein woh kisi kaam say busy hai to aap kabhi negative ki taraf nhi jaskte lekin yeh k agar main yhi sochongi k madam nay mujhe ignore kia phir to main negative ki sochongi na kabhi bhi positive nhi sochongi |  |
| Interviewer: Acha koi aur comments jo aap dena chahein koi aur baat jo aap batana chahein? |  |
| Interviewee: Main  to yhi kehti hon media k through media main akhbar tv aaj kal log akhbar to bohot kam prhte hain tv k through matlab takreeban further further matlab patients ko ya logon ko awareness di jaye k pregnancy main kaise kaise depression say nikla jata hai aur sath sath unko family planning k baare main bhi bataya jaye take unko zehn main ho k bhai hamne itne bache krne main | Media can be used to create awareness  Family planning issues also cause depression |
| Interviewer: Sahi |  |
| Interviewee: family planning ka to khair batate hi hain media main lekin depression k baare main  bhi batayen to usse bhi kaafi help hojati hai aur school aur colleges main bhi program honay chaheiy school main to bohot kam level main hongi lekin colleges main to honi chaheiy universities main honi chaheiy zaroori thori hai k abhi pregnant woman hai ham unko education dein usme yeh hota hai kam say kam woh future ki maaein to banengy na | Programs in schools and colleges and universities  Students are mothers of the future |
| Interviewer: bilkul bilkul sahi bas aur kuch? |  |
| Interviewee: nhi |  |
| Interviewer: chalein bohot bohot shukria apka |  |
